# Supplementary material for: Description of maternal and neonatal adverse events in pregnant people immunised with COVID-19 vaccines during pregnancy in the CLAP NETWORK of sentinel sites: nested case–control analysis of the immunization-associated risk – a study protocol
Source: BMJ Open. 2024 Jan 29;14(1):e073095. doi: 10.1136/bmjopen-2023-073095 (PMC10826566; doi:10.1136/bmjopen-2023-073095)
Supplement: Supplementary data [file bmjopen-2023-073095supp001.pdf]

This color means **WATCH** (does not necessarily indicate risk or inadequate practices)

PERINATAL CLINICAL RECORD - CLAP/WR-PAHO/WHO

FIRST NAME | LAST NAME
ADDRESS
DISTRICT | PHONE.

DATE OF BIRTH: day, month, year
AGE (years): < 15 > 35
ETHNIA: white, indigenous, mixed, black, other
LITERACY: no, yes
EDUCATION: none, primary, secondary, university; years in highest level
CIVIL STATUS: married, common mar., single, other; lives alone: no, yes
Place of antenatal visits | Place of birth | ID #

HISTORY

FAMILY (no, yes) | PERSONAL (no, yes): TBC, diabetes (I, II, G), hypertension, pre-eclampsia, eclampsia, other severe med. cond.

surgery of UT-UG, infertility, heart dis., kidney dis., violence, HIV+ (no, yes)

OBSTETRIC: prev. gest; abortions; vaginal; livebirths; alive
LAST PREVIOUS: n/a, < 2500g; normal, ≥ 4000g
3 consec. spont. ab.; deliveries; cesar. sections; tubal pregnancy; stillbirths; died 1st week; died after 1st week
History of twins: no, yes

END PREVIOUS PREGNANCY: day, month, year; < 1 year
PLANNED PREGNANCY: no, yes
CONTRACEP. METHOD FAILURE: no method, barrier, IUD, horm., emerg., natural

CURRENT PREGNANCY

PREVIOUS WEIGHT (Kg) | HEIGHT (cm) 1
DLM: day, month, year | EDD: day, month, year
GA BASED on: DLM, Us <20 w. (no, yes)
ACT SMO, PAS SMO, DRUGS, ALCOHOL, VIOLENCE (no, yes): 1st trim, 2nd trim, 3rd trim
VACCINES: no; yes before pregnancy; yes during pregnancy; yes after delivery/abortion; day, month, year; number of doses; gest. age weeks
tetanus diphtheria; tdap; influenza; rubella; hepatitis B; hepatitis A
screening hepatitis B: no, yes

NORMAL EX. (no, yes): DENTAL, BREAST
CERVIX (normal, abnormal, not done): Visual insp, PAP, COLP
GROUP | Rh (–, +) | Inmun (no, yes) | γglobulina anti D (no, yes, n/a)
TOXOPLASMOSIS (–, +, not done): <20 week IgG; ≥20 week IgG; 1 visit IgM
Hb <20 wk: g/dl, <11.0 g/dl
Presciption Fe/Folates: Fe, Folates (no, yes)
Hb ≥20 wk: g/dl, <11.0 g/dl

CHAGAS: –, +, not done
MALARIA: –, +, not done
BACTERIURIA: normal, abnormal, not done; wk <20, ≥20
FASTING BLOOD SUGAR: <20 wk mg/dl; ≥ 30 wk mg/dl
G.B.S: 35-37 weeks; –, +, not done
PREPARING FOR CHILDBIRTH: no, yes
ADVICE ON BREAST-FEEDING: no, yes

HIV - Diag Treatment: <20 weeks test request (yes, no, n/a), result (+, –, n/d, n/a); ART in preg. (yes, no, n/a); ≥20 weeks test request (Si, No, n/a), result (+, –, n/d, n/a); ART in preg. (yes, no, n/a)

SYPHILIS Diagnosis and treatment: Test no treponemic (–, +, n/d), treponemic (–, +, n/d, n/a); <20 wk weeks; Treatment (no, yes, n/d, n/a) weeks; Treat. partner (no, yes, n/d, n/a); Test ≥20 wk no treponemic (–, +, n/d), treponemic (–, +, n/d, n/a); Treatment (no, yes, n/d, n/a) weeks; Treat. partner (no, yes, n/d, n/a)

ANTENATAL VISITS

day	month	year	gest age	weight	BP	uterine height	presentation	FHR (bpm)	fetal movements	proteinuria	place of control	alarm signs, test, treatments	Technician's initials	next visit
				,										
				,										
				,										
				,										
				,										

CHILDBIRTH / ABORTION

DATE OF ADMISSION: day, month, year
CARD: no, yes
PRENATAL VISITS: total
AGE GEST. 1st CONSULT.: weeks
HOSPITAL during PREGNANCY: no, yes; days
ANTENATAL STEROIDS: complete, incompl., no, n/a; week started
ONSET: spontan., induced, elect. ces.
ANTEPARTUM RUPT. OF MEMBRANES: no, yes; day, month, year; hour, min; <37 wk; ≥18 hs.; temp. ≥38°C
GEST AGE AT CHILDBIRTH: weeks, days; by LMP, by US
PRESENTATION SITUATION: cephalic, breech, transverse
FETAL SIZE ADEQUATE: no, yes
COMPANION (labor, del): couple, relative, other, no one

LABOR (yes, no; details in partograph)

hour	min	mother position	BP	pulse	contr./10'	dilatation	height present.	variety posit.	mecon	FHR/dips

DISEASES: Labor Test (–, +, n/d, n/a): Syphilis, HIV; ART: yes, no, n/a

BIRTH

ALIVE | DEAD: antepartum, intrapartum, not known when
hour, min, day, month, year
MULTIPLE: no, yes; order
TERMINATION: spont., c-sect, forceps, vacuum, other
MAIN INDICATION FOR INDUCTION OR SURGERY: code; INDUC., SURG

POSITION LABOR: sitting, recumb, squat
EPISIOTOMY: no, yes
TEARS Grade (1 to 4): no
OXYTOCIC: before expul. plac. (no, yes), after (no, yes)
PLACENTA: complete, retained (no, yes)
EARLY CORD CLAMPING: < 1 min., 1 to 3 min.
MEDICATION RECEIVED (no, yes): oxytocic at labor, ATB, analges, local anesth, region anesth, gral anesth, transfusion, Mg sulfate (preeclam., eclam.), others (code): medic 1, medic 2

NEONATE

SEX: f, m, not defined
BIRTH WEIGHT: g; <2500 g; ≥4000 g
HEAD CIRC cm | LENGTH cm
GESTATIONAL AGE: weeks, days; LMP, US; ESTIMATED
WEIGHT G.A.: adeq., small, large
APGAR (min): 1st, 5th
Early intitiation of breast feeding: no, yes
RESUSCITATION (no, yes): stimul., suction, mask, oxygen, massage, tube, medication
ATTENDED: doctor, obst. nuerse, assist. stud., TBA, other, Name — CHILDBIRTH, NEONATE

CONGENITAL DEFECTS: no, minor, major; code
DISEASES: 1 o more, none; code
NEONATAL SCREENING:
HIV in NN: Exposed (no, yes, s/d), Treated (no, yes, s/d, n/a)
VDRL: –, +, not done; Tx: no, yes, n/a, s/d
Hearing, Chagas disease, Bili rrub, Toxo IgM, Hb pathy, Cardio-vascular disease: –, +, not done
Metabolic: not done, done
Death at site of birth (no, yes): Mother, NN
REFERRAL: room in, neonatol, another hosp.

POSTPARTUM

time	Temperature °C	B.P.	pulse	uterine involution	Bleeding	Responsible
	,					
	,					
	,					
	,					
	,					
	,					
	,					

γglobulin anti D: no, yes, n/a
Responsabile

NEONATAL DISCHARGE: alive, dead; day, month, year; hour, min; transfer; place
dies during or at transfer: no, yes, s/d
AGE AT DISCHARGE complete day: <1 day
FEEDING AT DISCH: excl. breastf, partial, artificial
Supine: no, yes | BCG: no, yes | INMUN. HEPATITIS B: no, yes
Meconium 1st day: no, yes
WEIGHT AT DISCHARGE: g
Id. NN | Name NN

HCP NEAR MISS ANV_ENG 02-08-201087

M O R B I L I T Y

HYPERTENSIVE DISORDERS (no / yes)

	no	yes
Chronic hypertension	○	○
Mild preeclampsia	○	○
Severe preeclampsia	○	○
Eclampsia	○	○
HELLP	○	○
Gestational Hypertension	○	○
Chronic hypertension + preeclampsia	○	○

INFECTIONS (no / yes)

	no	yes		no	yes
Sepsis	○	○	Pyelonephritis	○	○
Endometritis	○	○	Pneumonia	○	○
Chorioamnionitis	○	○	Caesarean section wound infection	○	○
Asymptomatic bacteriuria	○	○	Episiorrhaphy infection	○	○
			Other infections	○	○

HEMORRHAGE

1st TRIMESTER (no / yes) — 2nd TRIMESTER (no / yes) — 3rd TRIMESTER (no / yes)

	no	yes		no	yes
Post abortion	○	○	Uterine rupture	○	○
Hydatidiform Mole	○	○	Postpartum hemorrhage	○	○
Ectopic pregnancy	○	○	Uterine atony	○	○
Previous placenta	○	○	Lacerations	○	○
Accretism	○	○	Ovular remains	○	○
Abruptio placentae	○	○	Coagulation defect	○	○

METABOLIC DISORDERS (no / yes)

MELLITUS DIABETES (no / yes)

	no	yes	not performed
Abnormal oral tolerance to glucose load	○	○	○
Previous M. D. (insulin dependent)	○	○	
Previous M. D. (non insulin dependent)	○	○	
Gestational DM	○	○	
Hyperosmolar state	○	○	
Cetoacidosis	○	○	
Hyperglycemic state	○	○	

THYROID DISORDERS (no / yes)

	no	yes
Hypothyroidism	○	○
Hyperthyroidism	○	○
Thyroid crisis	○	○
Another metabolic disorder	○	○

OTHER DISORDERS (no / yes)

	no	yes		no	yes
Hyperemesis gravidarum	○	○	Oliguria	○	○
Deep vein thrombosis	○	○	Anemia	○	○
Pulmonary embolism	○	○	Sickle cell anemia	○	○
Amniotic fluid embolism	○	○	Renal disease	○	○
Heart disease	○	○	Malignant neoplasia	○	○
Valvulopathy	○	○	Psychiatric disorder	○	○
Seizures	○	○	Cholestasis	○	○
Altered state of consciousness	○	○	Others	○	○

OBSTETRIC COMPLICATIONS (no / yes)

	no	yes		no	yes
Obstructed labor	○	○	Polyhydramnios	○	○
Prolonged rupture of membranes	○	○	Acute fetal distress	○	○
Oligoamnios	○	○	Intrauterine growth restriction	○	○
Other	○	○			

INTERVENTIONS

	no	yes	s/d
Manual removal	○	○	○
Uterotonic to treatment of hemorrhag	○	○	○

Which ones?________

	no	yes	s/d
Central venous catheter	○	○	○
Administration of blood products	○	○	○

Nº of vol. [|]

	no	yes	s/d
Laparotomy (excluding caesarean section)	○	○	○
ICU admission <7 days	○	○	○
Using ATB iv to treatment of infectious complication	○	○	○

days [|]

Which ones?________

	no	yes
No pneumatic shock-suit	○	○
Hydrostatic balls	○	○
B-Lynch suture or similar	○	○
Hemostatic ligature of uterine / hypogastric arteries	○	○
Embolizations	○	○

VARIABLES TO IDENTIFY NEAR MISS CASES (no / yes)

CLINICAL CRITERIA / BODY-SYSTEM DYSFUNCTION

	no	yes
Cardiovascular		
Shock	○	○
Heart attack	○	○
Hepatic		
Jaundice in preeclampsia	○	○
Respiratory		
Acute cyanosis	○	○
Wheezing (gasping)	○	○
Severe tachypnea RF> 40 rpm	○	○
Severe bradypnea RF <6 rpm	○	○
Renal		
Oliguria non responsive to fluids or diuretics	○	○
Hematological/coagulation		
Failure to form clots	○	○
Neurological		
Coma	○	○
Prolonged unconsciousness (lasting >12 hours	○	○
Stroke	○	○
Uncontrollable fits/status epileptics	○	○
Total paralysis	○	○

LAB

Laboratory	no	yes
Platelets/ml < 50.000	○	○
Creatinine ≥ 300 µmol/l or ≥ 3,5 mg/dl	○	○
Bilirubin > 100 µmol/l or > 6,0 mg/dl	○	○
pH < 7,1	○	○
Sat Hb < 90% ≥ 1 hour	○	○
PaO_2/FiO_2 < 200 mmHg	○	○
Lactato > 5 µmol/L o 45 mg/dl	○	○

INTERVENTIONS

	no	yes
Use of continuous vasoactive drug	○	○

Which ones?________

	no	yes
Intubation and ventilation not related to anesthesia	○	○

days [|]

Criteria Near Miss: FLASOG ≥ 3 vol. WHO ≥ 5 vol.

	no	yes
Massive transfusion of blood or red cells (≥ 3 units)	○	○
Admission to UCI ≥ 7 days	○	○
Histerectomy	○	○
Dialysis for acute renal failure	○	○
Cardiopulmonary resucitation	○	○

days [|]

PUERPERIUM

day	Temp ºC	blood prasure	pulse	uter. invol.	lochia	perine	breast feeding	observations	Responsabile
1st	,								
2nd	,								
3rd	,								
5th to 10th	,								

MATERNAL DISCHARGE

DATE: day / month / year — hour / min

TYPE OF DISCHARGE: dead ○ — against medical advise ○ — discharge ○

CONDITION AT DISCHARGE: healthy ○ — NA ○ — with pathology ○ — dead ○

Place — Transfer [| | | | | | | |]

passes away during or after transfer: no ○ yes ○

Autopsy: no ○ yes ○

Responsabile

Contraception councelling: oral ○ — written ○ — none ○

Responsabile

CONTRACEPTION

Start contraception: no ○ — yes ○

	pref.	acced.
hormonal contraception	○	○
Other hormonal (vaginal ring, patch)	○	○
IUD	○	○
male surgical sterilization	○	○
injectable	○	○
hormonal implant	○	○
barrier	○	○
condom	○	○
tube ligation	○	○
abstinence	○	○

This color means **WATCH** (does not necessarily indicate risk or inadequate practices)

HCP NEAR MISS REVERSO (Regional) INGLES 06-08-2018
