# Supplementary material for: Description of maternal and neonatal adverse events in pregnant people immunised with COVID-19 vaccines during pregnancy in the CLAP NETWORK of sentinel sites: nested case–control analysis of the immunization-associated risk – a study protocol
Source: BMJ Open. 2024 Jan 29;14(1):e073095. doi: 10.1136/bmjopen-2023-073095 (PMC10826566; doi:10.1136/bmjopen-2023-073095)
Supplement: Supplementary data [file bmjopen-2023-073095supp002.pdf]

LAST NAME

Country code | Type of doc. | ID number

Had COVID-19 in the 12 months before the pregnancy termination?

	yes	no	Date (month / day / year)	Diagnostic test: PCR	Rapid	doesn't know	Asymptomatic - home	Symptomatic - home	Inpatient – intermediate	Inpatient – intensive
Before pregnancy	○	○	month / day / year	○	○	○				
During pregnancy 1st. contagion	○	○	month / day / year	○	○	○	○	○	○	○
During pregnancy 2nd contagion	○	○	month / day / year	○	○	○	○	○	○	○

Indicate the greater severity of the contagion(s) during pregnancy

Did she receive any dose of the COVID-19 vaccine in the last year? yes ○ no ○ **(If yes, indicate from the most recent to the oldest)**

Date		Gestational age	Did she present certificate/card? yes	no	AstraZeneca (AZD1222, ChAdOx1 nCoV-19, ChAdOx1-S, Vaxzevria, Covishield)	Janssen (JNJ-78436735; Ad26.COV2-S)	Pfizer/BioNtech (BNT162b2; Tozinameran; Comirnaty)	Sputnik V (Gamaleya; Gam-COVID-Vac; Adeno-based (rAd26-S+rAd5-S))	Sinopharm/ BIBP (Sinopharm/BIB)	Sinovac (CoronaVac; VeroCell)	Moderna (mRNA-1273; Elasomeran)	doesn't know	Other
month / day / year	or		○	○	○	○	○	○	○	○	○	○	○ Which one?:
month / day / year	or		○	○	○	○	○	○	○	○	○	○	○ Which one?:
month / day / year			○	○	○	○	○	○	○	○	○	○	○ Which one?:
