# Supplementary material for: Description of maternal and neonatal adverse events in pregnant people immunised with COVID-19 vaccines during pregnancy in the CLAP NETWORK of sentinel sites: nested case–control analysis of the immunization-associated risk – a study protocol
Source: BMJ Open. 2024 Jan 29;14(1):e073095. doi: 10.1136/bmjopen-2023-073095 (PMC10826566; doi:10.1136/bmjopen-2023-073095)
Supplement: Supplementary data [file bmjopen-2023-073095supp003.pdf]

Supplemental material III. Births attended and estimated number of potential cases per year.

Country	Hospital Name	Deliveries per year	Number of potential events per year		
			Small for gestational age	Preterm	Low birth weight
Bolivia	Hospital Boliviano Japonés	2719	177	337	185
	Maternidad de la Paz	1500	98	186	102
Colombia	Clínica Maternidad Rafael Calvo	7000	455	868	476
	Hospital Universitario del Valle, Cali	7200	468	893	497
Honduras	Hospital Roberto Suazo Cordova; Hospital de San Felipe; Leonardo Martinez Valenzuela; Hospital Berta Calderón Roque	27300	1775	3385	1856
República Dominicana	Hospital San Lorenzo de Los Mina	13000	845	1612	884
Argentina	Maternidad Martin	4200	273	349	291
	Roque Saenz Peña	2500	163	310	170
	Eva Peron	1500	98	186	102
Ecuador	Isidro Ayora	7000	455	868	476
Uruguay	Centro hosp Pereira Rosell	5000	325	620	340
	Hospital de Clinicas	800	52	99	54
Total SIP PLUS	-	93962	5731	10932	5995
