# Supplementary material for: Description of maternal and neonatal adverse events in pregnant people immunised with COVID-19 vaccines during pregnancy in the CLAP NETWORK of sentinel sites: nested case–control analysis of the immunization-associated risk – a study protocol
Source: BMJ Open. 2024 Jan 29;14(1):e073095. doi: 10.1136/bmjopen-2023-073095 (PMC10826566; doi:10.1136/bmjopen-2023-073095)

Figure. Schematic example of the matching strategies for PTB cases: Approach A “matched by date of birth” and Approach B “ matched by conception time”

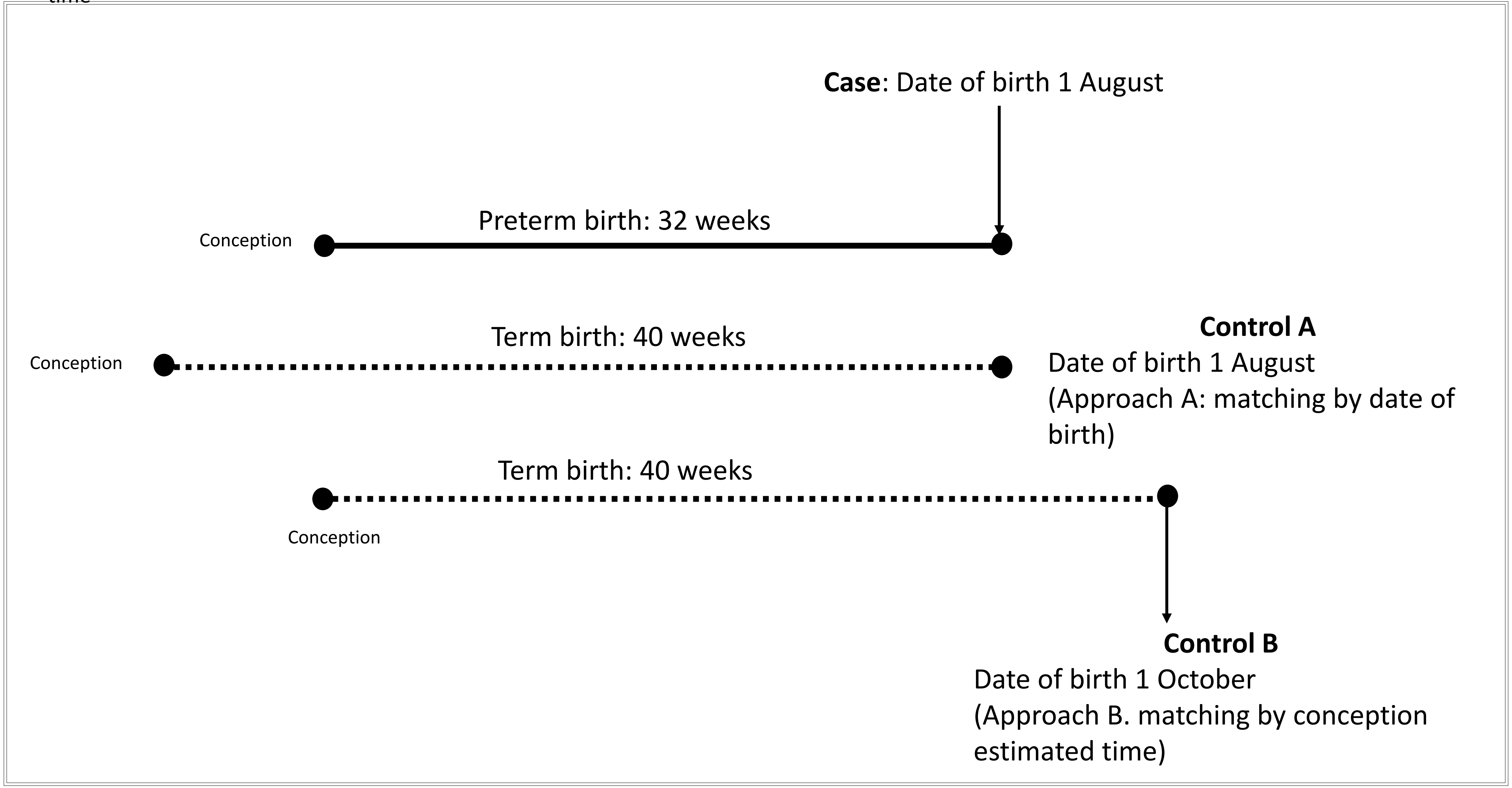

Supplement: Supplementary data [file bmjopen-2023-073095supp004.pdf]
