# Supplementary material for: Description of maternal and neonatal adverse events in pregnant people immunised with COVID-19 vaccines during pregnancy in the CLAP NETWORK of sentinel sites: nested case–control analysis of the immunization-associated risk – a study protocol
Source: BMJ Open. 2024 Jan 29;14(1):e073095. doi: 10.1136/bmjopen-2023-073095 (PMC10826566; doi:10.1136/bmjopen-2023-073095)

BMJ Open

BMJ Open is committed to open peer review. As part of this commitment we make the peer review history of every article we publish publicly available.

When an article is published we post the peer reviewers' comments and the authors' responses online. We also post the versions of the paper that were used during peer review. These are the versions that the peer review comments apply to.

The versions of the paper that follow are the versions that were submitted during the peer review process. They are not the versions of record or the final published versions. They should not be cited or distributed as the published version of this manuscript.

BMJ Open is an open access journal and the full, final, typeset and author-corrected version of record of the manuscript is available on our site with no access controls, subscription charges or pay-per-view fees (http://bmjopen.bmj.com).

If you have any questions on BMJ Open's open peer review process please email info.bmjopen@bmj.com

Description of maternal and neonatal adverse events in women immunized with COVID-19 vaccines during pregnancy in the CLAP NETWORK of sentinel sites. Nested case-control analysis of the immunization-associated risk: A study protocol.

Journal:	*BMJ Open*
Manuscript ID	bmjopen-2023-073095
Article Type:	Protocol
Date Submitted by the Author:	02-Mar-2023
Complete List of Authors:	Macías Saint-Gerons, Diego ; Department of Medicine, University of Valencia, INCLIVA Health Research Institute and CIBERSAM, Department of Medicine; PAHO<br>Castro, José ; Fundación para la innovación, la formación, la investigación y el desarrollo comunitario (FUNDEC); PAHO<br>Colomar, Mercedes; Unidad de Investigación Clínica y Epidemiológica Montevideo; PAHO, Latin American Center of Perinatology, Women and Reproductive Health (CLAP/WR)<br>Rojas, Robin; PAHO<br>Sosa, Claudio; Universidad de la Republica Uruguay, Department of Obstetrics and Gynecology, Pereira Rossell Hospital, School of Medicine; PAHO, Latin American Center of Perinatology, Women and Reproductive Health (CLAP/WR)<br>Ropero, Alba ; PAHO<br>Serruya, Suzanne; PAHO, Latin American Center of Perinatology, Women and Reproductive Health (CLAP/WR)<br>Pastor, Desiré; PAHO<br>Chiu, Monica; PAHO<br>Velandia-Gonzalez, Martha; PAHO<br>Abalos, Edgardo; Centro de Estudios de Estado y Sociedad (CEDES)<br>Durán, Pablo; PAHO, Latin American Center of Perinatology, Women and Reproductive Health (CLAP/WR)<br>Gomez Ponce de León, Rodolfo; PAHO, Latin American Center of Perinatology, Women and Reproductive Health (CLAP/WR)<br>Tomasso, Giselle; Unidad de Investigación Clínica y Epidemiológica Montevideo; PAHO, Latin American Center of Perinatology, Women and Reproductive Health (CLAP/WR)<br>Mainero, Luis; PAHO, Latin American Center of Perinatology, Women and Reproductive Health (CLAP/WR)<br>Rubino, Marcelo; PAHO, Latin American Center of Perinatology, Women and Reproductive Health (CLAP/WR)<br>De Mucio, Bremen; PAHO, Latin American Center of Perinatology, Women and Reproductive Health (CLAP/WR)
Keywords:	PERINATOLOGY, COVID-19, Maternal medicine < OBSTETRICS

Description of maternal and neonatal adverse events in women immunized with COVID-19 vaccines during pregnancy in the CLAP NETWORK of sentinel sites. Nested case-control analysis of the immunization-associated risk: A study protocol.

Diego Macías Saint-Gerons (0000-0002-2572-2160), José Luis Castro (0000-0002-9179-113X), Mercedes Colomar (0000-0001-7424-5551), Robin Rojas (0000-0002-4046-9298), Claudio Sosa (0000-0002-2539-0848), Alba Maria Ropero (0000-0001-9960-7491), Suzanne Serruya (0000-0003-1371-4558), Desiré Pastor, Monica Chiu (0000-0002-2730-8625), Martha Velandia (0000-0002-7636-3604), Edgardo Abalos (0000-0001-6653-429X), Pablo Durán (0000-0003-1032-487X), Rodolfo Gómez Ponce de León (0000-0001-9914-7130), Giselle Tomasso (0000-0002-4424-1581), Luis Mainero, Marcelo Rubino, Bremen de Mucio (0000-0003-0662-9742)

Department of Medicine, University of Valencia, INCLIVA Health Research Institute and CIBERSAM, Department of Medicine, Valencia, Comunitat Valenciana Diego Macías Saint-Gerons International PAHO consultant, FÜNDEC: Fundación para la innovación, la formación, la investigación y el desarrollo comunitario, Santa Cruz de Tenerife José Luis Castro International PAHO consultant, Montevideo Clinical and Research Unit. Hospital de Clinicas, 11300 Montevideo, Uruguay Mercedes Colomar International PAHO consultant,

Pan-American Health Organization (PAHO), 525 23rd St NW, Washington DC 20037, United States Robin Rojas Technical Officer, Universidad de la República Uruguay, Department of Obstetrics and Gynecology, Pereira Rossell Hospital, School of Medicine, 11300 Montevideo, Uruguay Claudio Sosa International PAHO consultant, Pan-American Health Organization (PAHO), 525 23rd St NW, Washington DC 20037, United States Alba Maria Ropero Regional Advisor on Immunization, Latin American Center of Perinatology, Women and Reproductive Health (CLAP/WR), Av. Brasil 2697, 11300 Montevideo, Uruguay Suzanne Serruya CLAP Director, Pan-American Health Organization (PAHO), 525 23rd St NW, Washington DC 20037, United States Desiré

Pastor Regional Immunization Advisor, Pan-American Health Organization (PAHO), 525 23rd St NW, Washington DC 20037, United States Monica Chiu International PAHO consultant, Pan-American Health Organization (PAHO), 525 23rd St NW, Washington DC 20037, United States Martha Velandia Advisor Immunization, Centro de Estudios de Estado y Sociedad (CEDES), Sánchez de Bustamante 27, Ciudad Autónoma de Buenos Aires, Argentina, Edgardo Abalos CLAP Associate researcher, Latin American Center of Perinatology, Women and Reproductive Health (CLAP/WR), Av. Brasil 2697, 11300 Montevideo, Uruguay Pablo Durán advisor, Latin American Center of Perinatology, Women and Reproductive Health (CLAP/WR), Av. Brasil 2697, 11300 Montevideo, Uruguay Rodolfo Gómez Ponce de León advisor, Montevideo Clinical and Research Unit. Hospital de Clinicas, 11300 Montevideo, Uruguay Giselle Tomasso International PAHO consultant, Latin American Center of Perinatology, Women and Reproductive Health (CLAP/WR), Av. Brasil 2697, 11300 Montevideo, Uruguay Luis Mainero IT expert, Latin American Center of Perinatology, Women and Reproductive Health (CLAP/WR), Av. Brasil 2697, 11300 Montevideo, Uruguay Marcelo Rubino data analyst, Latin American Center of Perinatology, Women and Reproductive Health (CLAP/WR), Av. Brasil 2697, 11300 Montevideo, Uruguay Bremen de Mucio Advisor.

Correspondence to: Mercedes Colomar; +59899114204; mcolomar@unicem-web.org; Julio Maria Sosa 2281, CP 11300, Montevideo, Uruguay.

Word count: 4461

Keywords:

Perinatology; Maternal Health; Pharmacovigilance; Infant, Premature Birth; Infant, Small for Gestational Age; Low Birth Weight; Pregnancy Outcome; COVID-19 vaccines.

Protocol summary

Introduction

Coronavirus disease is associated with higher morbi-mortality in pregnant women compared to non-pregnant women. At present, the benefits of maternal immunization

For peer review only

are considered to outweigh the risks and therefore vaccination is recommended at any time during pregnancy. However, additional information is needed on the safety of the vaccines in this population.

Methods and analysis

This a retrospective cohort nested case–control study in pregnant women who attended maternity hospitals from 8 Latin American and Caribbean countries. Hospital´s characteristics can be found in table 1. A perinatal electronic clinical history database with data from neonatal and obstetric monitoring will be used (12 months; approximately 75,000 deliveries). The proportion of pregnant women immunized with COVID-19 vaccines of the following maternal and neonatal events will be described: preterm infant, small for gestational age, low birth weight, stillbirth, neonatal death, congenital malformations, maternal near miss, and maternal death. Moreover, the risk of prematurity, small for gestational age, and low birth weight associated with exposure to COVID-19 vaccines will be. Each case will be matched with 3 randomly selected controls at the same delivery date of each case (index date) and matched by hospital and mother's age (+/- 3 years). The estimated required sample size for the main analysis (exposure to any vaccine) concerning "non-use" is at least 1009 cases (3,027 controls) to detect an increased probability of vaccine-associated event risk of 30% (OR=1.3) and at least 650 cases (1,950 controls) to detect 30% protection (OR=0.7). Sensitivity and secondary analyses considering country, type of vaccine, exposure windows, and completeness of immunization will be reported.

Ethics and dissemination

The study protocol was reviewed by the Ethical Review Committee on Research of the Panamerican Health Organization. Patient informed consent was waived due to the retrospective design and the utilization of anonymized data (Ref. No: PAHOERC.0546.01. Results will be disseminated in Open Access journals.

Strengths and limitations of this study

- Pregnant women were excluded from the pivotal clinical trials of COVID-19 vaccines and most of the evidence from vaccine safety comes from high income country studies. This study represents an opportunity to generate

For peer review only - http://bmjopen.bmj.com/site/about/guidelines.xhtml

complementary evidence of maternal, foetal and neonatal adverse events after administration of COVID-19 vaccines currently used in LAC countries for maternal immunization.

- The use data from the SIP-PLUS database allows achieving a large sample size required to explore potential vaccine-associated discrete effects (beneficial or harmful) in a timely manner.
- The number of “non exposed” pregnant women could decrease over time and may introduce affect statistical power and interpretation.
- As in any study using routinely collected data there are further potential limitations due to incomplete data records, inaccuracies (dates), unmeasured confounding factors, and missing data.

For peer review only

Introduction

Pregnant women do not appear to have a higher susceptibility to SARS-CoV-2 coronavirus infection than nonpregnant women.[1] However, coronavirus disease (COVID-19) is associated with higher morbidity and mortality among the former women than among the latter.[2,3] Mortality in pregnant women with COVID-19 could be 70% higher, and the risk of admission to intensive care units (ICU) or the need for mechanical ventilation would be three times higher compared to non-pregnant women.[3,4] Pregnant women with COVID-19 would have an increased risk of occurrence of some adverse maternal-fetal outcomes such as increased risk of preterm delivery, preeclampsia, or miscarriage compared to pregnant women without COVID-19.[2] It has been observed that, in its most complicated or critical forms, COVID-19 infection is associated with prematurity, gestational diabetes, preeclampsia and low birth weight.[3] Neonates born to mothers with COVID-19 appear to have a higher probability of admission to a neonatal care unit (NCU), which could be attributed to a greater extent to the negative effect of COVID-19 on maternal health during gestation than to vertical transmission.[4–6] Besides other maternal, fetal, and neonatal complications associated with COVID-19 have also been reported. A systematic review described adverse events such as fetal distress, premature rupture of membranes, and other neonatal adverse events such as

For peer review only - http://bmjopen.bmj.com/site/about/guidelines.xhtml

respiratory distress, gastrointestinal events, and fever. Maternal death reports have been more rare.[7]

After a global research effort to develop effective treatments and vaccines against COVID-19, vaccines became available on January 2021. It should be noted that a characteristic of the new COVID-19 vaccines is that they were developed from a range of different platforms: viral vector (adenoviral), mRNA platform, inactivated, attenuated viruses, being pregnancy an exclusion criterion in the main pivotal clinical trials undertaken for the authorization of these COVID-19 vaccines.[8] Besides, vaccines that are currently recommended for maternal immunization - and with which there is most experience - such as the influenza vaccine or the Tdap vaccine, do not use mRNA-based platforms as is the case with the Pfizer-BioNTech and Moderna vaccines.[9] However, despite being based on a newer platform, mRNA vaccines do not entail the risk of acquiring vaccination-induced infection, as might be the case with the use of attenuated/inactivated vaccines. On the other hand, they could be useful in pregnant women, as a robust transfer of the humoral response to the fetus has been observed with mRNA vaccines.[10,11] Furthermore, no remarkable adverse events have been detected in experimental genotoxicity studies conducted on rats.[12]

Although various clinical trials have been planned for the study of COVID-19 vaccines in pregnant women, most of the initiatives are based on the creation of registries of pregnant women exposed to the vaccines. In many cases, it is the adaptation of previous registries that had been created to study the effects of COVID-19 infection in pregnant women. In this respect, Moderna created a registry to monitor pregnancy outcomes in individuals who received their COVID-19 vaccine. PREGISTRY - for example - is a startup focused on improving maternal-fetal health, in collaboration with the Harvard School of Public Health (https://www.pregistry.com); aim to enroll 8,000 pregnant women in their registry to evaluate their obstetric, neonatal, and infant outcomes following COVID-19 immunization. In the UK, the UKOSS registry (https://www.npeu.ox.ac.uk/ukoss) is collecting data to allow the parallel nesting of cohort and case-control studies. The CDC's "v-safe" registry have reported preliminary results with observed frequencies of spontaneous abortion and fetal death in vaccinated pregnant women similar to the

For peer review only - http://bmjopen.bmj.com/site/about/guidelines.xhtml

expected frequencies in the general population of pregnant women of the same age and without known comorbiditiests.[13]

Apart from these active registries that include pregnant women, there is little evidence from published studies with complete data on the effectiveness and safety of vaccination for COVID-19 in this population. One retrospective cohort study observed that, in pregnant women, vaccination with Pfizer-BioNTech was associated with a lower risk of SARS-CoV-2 infection compared to those who did not receive vaccination (HR:0.22; 95% CI: 0.11-0.43).[14] In another prospective cohort study, short-term adverse events associated with mRNA vaccines were similar among pregnant women to those occurring in nonpregnant women of the same childbearing age.[15] In another large comparative study involving 157,521 singleton pregnancies from the Swedish pregnancy and Norwegian childbirth electronic registries, no statistically significant differences were found in the risk for fetal death, small for gestational age rates, preterm, and low APGAR score value in mRNA and Pfizer-BioNTech vaccinated and unvaccinated subjects.[16] Also, the risk of miscarriage does not appear to be higher than expected among women immunized before or during pregnancy.[17,18] A case-control study using Norwegian registry data observed no statistically significant risk difference for first-trimester miscarriage in pregnant women exposed to COVID-19 vaccines (Pfizer-BioNTech, Moderna, Astra) and those not immunized.[19] A recent systematic review and meta-analysis included data from 23 studies and 117,552 pregnant women immunized mostly with mRNA vaccines. In this meta-analysis, which accumulates the most evidence on the subject to date, no differences were found in vaccinated compared to unvaccinated women with regards the risk of occurrence of miscarriage, earlier gestation at birth, placental abruption, pulmonary embolism, postpartum hemorrhage, maternal death, mothers' admission to the ICU, low birth weight, or newborns' admission to the ICU. The meta-analysis identified that immunization conferred a 15% protective effect against fetal death, although the heterogeneity found was very high (Odds Ratio: 0.85; 95%CI: 0.73-0.99; I^2 = 93.9%).[20] Some absolute estimates of the impact of vaccination with mRNA vaccines have been proposed to assess the number of maternal and neonatal adverse outcomes that could be avoided such as "Number Needed to Vaccinate (NNV)". The impact measures suggest a benefit in avoiding certain

For peer review only - http://bmjopen.bmj.com/site/about/guidelines.xhtml

events such as COVID-19 infection (NNV:11), cesarean section delivery (NNV: 182) and prematurity (NNV: 200), while the NNV would be higher for "neonatal problems" (NNV: 463), severe cases of COVID-19 (NNV: 412-2,058), requirement for mechanical ventilation (NNV: 1,371-6,857) and small for gestational age (NNV: >2,000). In all cases far from the "number needed to harm" (NNH) estimated for some adverse events: myocarditis (NNH: 37,000) or thrombosis syndrome with thrombocytopenia (NNH: 50,000).[21] estimates carried out based on observational data in Israel to determine the "Number Needed to Boost" and assess the impact of the third booster dose of the Pfizer-BioNTech vaccine in preventing hospital admissions for COVID-19 and complicated COVID-19 would also suggest a greater potential benefit for pregnant women of advanced age or with comorbidities receiving booster doses.[22]

However, the majority of evidence suggesting the possible beneficial effect of vaccination comes from descriptive observational studies. A large population-based descriptive study included more than 180,000 pregnant women in Scotland. The results suggested a possible benefit of vaccination. Thus, 77.4% of all COVID-19 infections, 90.9% of hospital admissions, 98% of ICU hospitalizations and 100% of neonatal deaths occurred in women diagnosed with COVID who had not been vaccinated.[23] In addition, descriptive data from six European countries from the International Network of Obstetric Survey Systems (INNOSS) have also reported that the highest proportion of ICU admissions of pregnant women corresponded to women who had not been vaccinated (80-100% of the total).[24] On the other hand, in a study in the USA from electronic medical record data in women who had miscarriages, no differences were found in the probability of having been vaccinated (mRNA vaccines) 28 days before the occurrence of the miscarriage.[25]

At present, the benefits of maternal immunization are considered to outweigh the risks and for that reason vaccination is recommended at any time during pregnancy.[1,26,27] The recommendation is supported, on the one hand, by the robust evidence indicating a higher morbidity and mortality associated with COVID-19 among pregnant women compared to non-pregnant women; and on the other hand, by the protective effect of maternal immunization observed against complications of COVID-19. Moreover, no pharmacovigilance signals associated with maternal immunization have been

For peer review only - http://bmjopen.bmj.com/site/about/guidelines.xhtml

detected.[13] Finally, some studies suggest a decreased risk of occurrence for certain adverse fetal and neonatal outcomes associated with maternal immunization, although these are considerations based mostly on observational evidence, and in many cases with the caveat that further studies are needed. However, the great difference in the amount of evidence available according to the type of vaccine is striking. Most studies in pregnant women have been carried out with vaccines developed from the newest mRNA platform (Pfizer-BioNTech, Moderna), with very little evidence available for the other vaccines. Perhaps for this reason, some countries prioritize the use of mRNA vaccines in their recommendations, leaving the administration of other (non-mRNA) vaccines with a weaker recommendation based on clinical criteria, weighing the possible risk of infection of the mother considering certain circumstances (e.g.: health workers, comorbidities, etc.) with the theoretical risks that the uncertainties of immunization may entail.[28,29] In spite of the available evidence, most countries that have reported vaccination coverage in pregnant women had low vaccinations rates.[30] For example, in Scotland only 32.3% of pregnant women had received the full scheme (versus 77.4% of non-pregnant women of childbearing age).[23] Other countries have also reported low coverage rates among pregnant women such as Italy (20%) or England (22%) or 30-50% in the Netherlands. [24]

The Latin American Center of Perinatology (CLAP) coordinates a NETWORK of sentinel centers in Latin American and Caribbean countries for the surveillance of maternal health related issues. All centers use SIP-PLUS as a common data collection system. SIP-PLUS database contains the perinatal electronic health record data (Annex I). The perinatal medical record prospectively and systematically records individual data on the clinical follow-up of pregnant women seen from the date of the first antenatal visit to the date of discharge following delivery. In addition, it incorporates the systematic collection of information on the use of vaccines (tetanus/diphtheria, Tdap, influenza, rubella, hepatitis B, hepatitis A), as well as information on dosage, date of vaccination and time of vaccination (before or during pregnancy, or after delivery).[31] In response to the outbreak of the COVID-19 pandemic, CLAP developed a specific SIP form to monitor COVID vaccination of pregnant women, along with other related variables, such as

For peer review only - http://bmjopen.bmj.com/site/about/guidelines.xhtml

COVID-19 history, which have become part of the routine follow-up of pregnant women (Annex II).

Therefore, this project aims to retrospectively describe the use of COVID-19 vaccines among pregnant women and explore the risk associated with vaccination and selected adverse events, adapting the SIP-PLUS database for surveillance of the use and safety of COVID-19 vaccines from data routinely collected during obstetric and neonatal monitoring of pregnant women in a network of Latin American centers.

GENERAL AND SPECIFIC OBJECTIVES.

General objective.

To describe the utilization of COVID-19 vaccines by pregnant women in hospitals using the SIP-PLUS database and to analyze the risk of vaccination associated with selected maternal and neonatal events.

Specific objectives.

1. To describe the proportion of vaccinated individuals among pregnant women by type of COVID-19 vaccine, number of doses administered, and country.

2. Describe the frequency (Cumulative Incidence) of the following neonatal and maternal events in the cohort of pregnant women: preterm delivery, small for gestational age, low birth.

3. To analyze the associated risk of vaccination for the following neonatal events of interest through an exploration of case-controls nested in a cohort for the following events: prematurity, small for gestational age, and low birth weight.

Methods and Analysis

1. Design and rationale

Retrospective observational descriptive cohort and analytical study with a nested case-control approach. All outcome and exposure data will be available before the study start date (retrospective analysis).

For peer review only - http://bmjopen.bmj.com/site/about/guidelines.xhtml

The dedicated module for capturing COVID-19 vaccine exposure information (including possible heterologous combinations) will be used to obtain the data required. This module complements the current data routinely collected for the vaccines recommended during pregnancy and is collected as part of the obstetric clinical monitoring (Annex II).

2. Source of information and study period.

This study includes secondary data. All the necessary information, including socio-demographic data, comorbidities, exposure to vaccines and events will be retrieved from the SIP-PLUS perinatal electronic medical record database that electronically consolidates perinatal medical record data obtained during routine clinical practice over a retrospective 12-month period.

3. Population, outcome variables, exposure and others.

3.1 Retrospective description of vaccination and event frequency in the pregnant women cohort.

3.1.1 Inclusion criteria

All pregnant women admitted for termination of pregnancy (singleton delivery).

3.1.2 Exclusion criteria

Twin pregnancies will be excluded.

3.1.3 Adverse events of interest

1. Premature births (preterm delivery)
   Born alive at less than 37 weeks gestational age as registered in the SIP form Clinical record.
2. Small for gestational age. Live birth classified as small by country standards.
3. Low birth weight. Born alive weighing less than 2500 grams.
4. Stillbirth (at any time).
5. Non-extended neonatal death (max 48h postpartum).
6. Congenital malformations (major and minor).
7. Maternal death (at any time).

For peer review only - http://bmjopen.bmj.com/site/about/guidelines.xhtml
For peer review only

8. Maternal Near Miss (MNM). Women with a MNM condition according to WHO MNM approach.[32]
9. Abortion.

3.1.4 Covariates

Sociodemographic variables:

- Mother's age (years)
- Ethnicity (white, indigenous, mixed, other)
- Marital status (married, common-law marriage, single, other)
- Educational level (none, elementary, high school, college, university)
- Country
- Hospital

Obstetric variables and risk factors (definitions)

- Date of last menstrual period
- Smoking (yes/no)
- Drug use (yes/no)
- Alcoholism (yes/no)
- Induced labor (yes/no)
- Diabetes (yes/no)
- High blood pressure (yes/no)
- Eclampsia (yes/no)
- Preeclampsia (yes/no)
- Parity (nulliparous, multiparous)
- Inter-gestational period (months)
- COVID infection (describe diagnosis, prior to or during pregnancy)

Administration of other recommended vaccines during pregnancy

- Tetanus/diphtheria (yes/no)
- Tdap (yes/no)
- Hepatitis B(yes/no)
- Influenza (yes/no)

For peer review only - http://bmjopen.bmj.com/site/about/guidelines.xhtml

- Any of the other recommended vaccines (yes/no)

3.1.5 Exposure of interest (immunization with COVID-19 vaccines)

Type of vaccine, number of doses administered, and date.

The vaccines of interest will be

-Astra-Zeneca (AZD1222; ChAdOx1 nCoV-19; ChAdOx1-S; Vaxzevria, Covishield)

-Janssen (NJ-78436735; Ad26.COV2-S)

-Moderna (mRNA-1273; Elasomeran)

-Pfizer-BioNTech (BNT162b2; Tozinameran; Comirnaty)

-Sinopharm (Sinopharm/BIB)

-Sinovac (CoronaVac; VeroCell)

-Sputnik- Gamaleya (Gamaleya; Gam-COVIDVac; Adeno-based (rAd26-S+rAd5-S)

The rest of vaccines not considered in the list will be recorded in free text as "other vaccines". When the administration of two different vaccines is included, the vaccination will be classified as "heterologous schedule". On the other hand, it will be considered a complete schedule if the pregnant woman received 2 doses (or 1 dose in case of Janssen's vaccine) and a booster schedule if she has received 3 doses (or 2 doses in case of Janssen's vaccine).

3.2. Analysis of the risk associated with vaccination for the occurrence of the neonatal events of interest through an exploration of case-controls nested in a cohort.

A matched case-control approach nested in a cohort will be carried out for each of the events of interest.

3.2.1 Cohort definition (descriptive part)

- Pregnant women at term and with mother's age data

- Live births with delivery date available based on the best available obstetric estimator.

- Perinatal medical record with "COVID-19 vaccine module" implemented.

3.2.2 Case definition (Events of interest)

For peer review only - http://bmjopen.bmj.com/site/about/guidelines.xhtml

1. Premature. Preterm delivery: live birth at less than 37 weeks gestational age according to the best available obstetric estimator
2. Small for gestational age: live birth classified as small based on country standards.
3. Low birth weight: born alive weighing less than 2500 grams.

3.2.3 Definition of controls

Three (uneventful) controls will be randomly selected at the same delivery date of each case (index date) matched by hospital and mother's age (+/- 3 years).

3.2.4 Definition of exposure. The vaccines considered will be those mentioned in section 3.1.5. Exposure shall be classified according to the following exposure windows**:**

1. **"Late immunization"**: pregnant women immunized on the index date or up to one trimester before the end of pregnancy (third trimester)**.**
2. **Second trimester immunization":** pregnant women immunized after completing the first trimester until the second trimester before the event.
3. **"First trimester immunization"** pregnant women immunized during the first trimester.
4. **"Past immunization"** immunized prior to pregnancy
5. **"Immunization at any time".** Immunization at any of the above times**.**

The figure 1 summarizes the overall conceptual framework and procedures of this study.

4. Expected sample size and rationale for its determination.

4.1 General considerations

In estimating the sample size, the frequencies of events observed in a previous analysis in the SIP-PLUS database were considered to estimate the number of potential events (Annex III). Given the lack of precise data on vaccination coverage in hospitals in the CLAP network, literature values from studies published in other countries (prevalences ranging from 20 to 50%) were used as a reference.[23,24] The design of the contrast tests as bilateral (or two-tailed) is because the hypothesis contemplates the scenario of a possible protection against adverse events associated with vaccines and also the opposite scenario, with an increased risk. The correlation coefficient (r) between

For peer review only - http://bmjopen.bmj.com/site/about/guidelines.xhtml

(matched) cases and controls is not known and cannot be estimated from previous studies. Hence, a conservative value of "r=0.2" has been assumed for the calculation of the sample size, following the recommendation of some authors for these cases.[33] In this part we will consider the analysis of events for which, a priori, there would be sufficient statistical power, anticipating that the measures of association (Odds Ratio) may not be to large based on existing published studies.

4.2. Main analysis of vaccination (any vaccine) with respect to "non-use".

For the matched case-control study (matched with 3 controls) with a power of 80%, alpha error=0.05 (bilateral), a case-control correlation coefficient of 0.2 and a vaccine exposure of 20% would require at least 1009 cases (3,027 controls) to detect an increased probability of vaccine-associated event risk of 30% (OR=1.3) and at least 650 cases (1,950 controls) to detect 30% protection (OR=0.7) (Figures 2 and 3).

4.3. Estimation of the expected number of cases in the database

Based on previous estimates in the SIP-PLUS database and the annual number of deliveries in each hospital of the network, a sufficient number of cases could be expected annually: 5,731 cases of small for gestational age, 10,932 preterm, and 5,995 low birth weight babies.

5. Data analysis

5.1 Retrospective description (Cumulative Incidence) of the following maternal and neonatal events in the pregnant cohort: preterm, small for gestational age, low birth weight, stillbirth, neonatal death, congenital malformations, maternal near miss (MNM) and maternal death.

The cumulative incidence will be estimated together with the 95% confidence interval using the exact method. When calculating the cumulative incidence, the number of events of interest will be used in the numerator and different denominators according to the type of event.

1. "Live births" denominator: small for gestational age, premature, low birth weight, stillbirth, MNM, and maternal death.

For peer review only

2. "Total pregnancies" denominator: stillbirth, congenital malformations.

5.2 Description of the proportion of pregnant women who have received a dose of COVID-19 vaccine (total and by type of vaccine) and complete vaccination schedule.

The proportion of vaccinated women out of the total number of pregnant women in the database will be estimated. The proportions (prevalences) of immunization will be estimated by type of vaccine, vaccine platform (ARNm, Adenoviral vector-based), schedule (complete/incomplete) and time of administration (pre-pregnancy, first trimester, second trimester, third trimester). The total prevalence of maternal immunization (combination of all vaccines administered at any time) will also be estimated. The 95% confidence interval of the proportion will be calculated using the exact method.

5.3 Nested case-control analysis

Logistic regression models will be built for each event of interest, considering COVID-19 vaccine exposure as the main independent variable adjusting for possible confounders (covariates) and estimating the Odds Ratio together with its 95% confidence intervals (CI) as a measure of association (OR). The use of a "standard" (non-conditional conditional) logistic regression analysis will consider adjustment for potential confounders and in addition to adjustment variables, but in addition matched variables will be included in the adjusted models, as recommended for the minimization of bias in the analysis of matched case-control studies.[34] Two models will be presented: (1) an unadjusted model that includes only the matching variables (mother's age, date of delivery, and hospital) and (2) a fully adjusted model that also includes all possible confounders described above. As a rule, missing variable data will be treated as a separate (unknown) category. If necessary, missing data will be handled by multiple imputation methods. Analyses by individual vaccine type or platform will be explored. Sensitivity analysis will be performed for different exposure time windows considering the different types of exposure considered in the paper, and their classification according to proximity to the event (late vaccination, second trimester, early), completeness of the schedule (complete or incomplete schedule) and country. Sample size estimation were made using PS: Power and Sample Size Calculation (v. 3.1.6).[35]

For peer review only - http://bmjopen.bmj.com/site/about/guidelines.xhtml

Statistical analyses will be performed using Stata 17 statistical software (Stata Corp., College Station, TX).

It is estimated that a retrospective analysis of all the data will be possible around July 2023. In addition to the cohort description, the risk for adverse events of interest for which the planned sample size has been achieved will be explored. Controls will be obtained from a random selection of pregnant women in the SIP-PLUS database. Three controls will be included for each case matched by maternal age, hospital and delivery date.

6. Patient and public involvement

This protocol presents a retrospective register-based, non-experimental study, and will not have measures of intervention that could burden patients in any way assessable not constituting any risk for patients' participation, and it neither implies the realization of diagnostic tests or additional therapeutic decision besides of what the professional considers timely necessary as part of their clinical practice. Besides, the research question formulation was based upon our own priorities for patient benefit and result interpretation. The nested case–control study described by the protocol is based on clinical records from a network of sentinel sites and thus will not include a recruitment process for patients, who will not be involved in neither the design nor conduct of the study. All results will be distributed via the relevant pregnant women groups, as well as peer-reviewed journals and scientific conferences.

Strengths and Limitations

Risk of indication bias. It is possible that women at higher baseline risk for severe COVID-19 may be more likely to have been vaccinated than those not presenting these factors. Adjusted models will consider inclusion of risk factors (e.g., diabetes, hypertension, BMI≥30 kg/m2) as adjustment variables. [4,36]

Availability of controls. The number of controls available for case-matching may be small. In

field studies, obtaining controls is very expensive and getting the desired number may turn out unfeasible. However, in database studies controls are more readily available.

For peer review only - http://bmjopen.bmj.com/site/about/guidelines.xhtml
For peer review only

Possible lack of "non-exposure" to COVID-19 vaccines in pregnant women (case of massive recommendation for maternal immunization and full coverage). We understand that current recommendations are aimed at promoting maternal immunization with COVID-19 vaccines, but full coverage is not anticipated at present. However, an interim analysis is planned at the end of the year to assess the prevalence of exposure by that time, and to readjust the initial estimates of sample size and study potential before the start of the study.

Missing data. As in any study with secondary data, there are always limitations due to incomplete data records, inaccuracies (dates), unmeasured confounding factors, and missing data, etc. In case of missing data for a variable, these will be treated as a separate category in the analysis, and multiple imputation techniques will be considered whenever necessary.

Moreover, the description of abortions will be considered exploratory/hypothetical since: 1) it is not possible to differentiate whether the abortions recorded are spontaneous or induced and, 2) a significant proportion of spontaneous abortions occurs on the first weeks of pregnancy and they may not require hospital care nor attended in maternal hospital settings.

Finally, Randomized Clinical Trials (RCTs) are the gold-standard for causal inference. However comparative observational studies may be appropriate when RCTs are not feasible. With the new recommendations for maternal immunization issued by the CDC and WHO RCTs comparing COVID-19 vaccines vs placebo would be difficult to carry out since clinical equipoise will not be reached and administering placebo would be considered unethical. In this scenery retrospective observational studies can provide comparative evidence on COVID-19 vaccine effects versus unexposed pregnant women. Furthermore, information on COVID-19 vaccine safety in pregnant women might mainly be coming, as already mentioned, from high-income countries (HICs). This study represents an opportunity to generate complementary evidence of maternal, foetal and neonatal adverse events from hospitals in middle- and low-income settings but also to gather information for vaccines whose safety profile in pregnancy is less known such as vaccines non authorized nor utilized in HICs.

For peer review only

For peer review only - http://bmjopen.bmj.com/site/about/guidelines.xhtml

Ethics and dissemination

The present proposal will be implemented in compliance with the principles of the Declaration of Helsinki.[37] and the international standards for the conduct of epidemiological studies, contained in the International Guidelines for Ethical Review of Epidemiological Studies.[38] The reporting of results will conform to the guidelines for reporting routinely collected and observational pharmacoepidemiology health data (RECORD-PE).[39]The study protocol was reviewed by the Ethical Review Committee on Research of the Panamerican Health Organization. Patient informed consent was waived due to the retrospective design of the study and the utilization of anonymized data (Ref. No: PAHOERC.0546.01). Study results will be disseminated in Open Access journals.

This is an open access article distributed in accordance with the Creative Commons Attribution 4.0 International (CC BY 4.0) license, which permits others to copy and redistribute, remix, adapt, build upon this work for any purpose, even commercially, giving appropriate credit.

Benefit-risk assessment for participants

This retrospective study based on secondary data does not entail any additional risk for the patients because of their participation. Nor does it involve additional diagnostic tests, evaluations or therapeutic decisions beyond those deemed appropriate as part of routine clinical practice. However, the results of the proposal are expected to inform the understanding of the benefit-risk balance of maternal immunization against COVID-19 in the future.

Confidential treatment of data.

As the study will be carried out on an anonymized database without personal identifiers, an informed consent waiver was granted.

Authors' contributions

For peer review only - http://bmjopen.bmj.com/site/about/guidelines.xhtml
For peer review only

Diego Macías Saint-Gerons, Alba Maria Ropero, Bremen de Mucio, Claudio Sosa, José Luis Castro, Suzanne Serruya , and Mercedes Colomar wrote the proposal. Desiré Pastor, Monica Chiu, Martha Velandia, Robin Rojas, Edgardo Abalos, Pablo Durán, and Rodolfo Gómez Ponce de León reviewed the proposal and made remarkable contributions. Giselle Tomasso, Luis Mainero and Marcelo Rubino designed the clinical records. All the authors reviewed and agree with the protocol.

Funding statement

This study is funded by Health Canada: Grant 452029: "Access to COVID-19 Vaccines and Therapeutics for Populations in Situations of Vulnerability in the Americas and to support maternal immunization safety related activities"; U.S. Food and Drug Administration grant to support maternal immunization safety related activities, and by the US government (USG) funds through the American Rescue Plan Act.

Competing interest statement.

The authors declare that they have no known competing financial interests or personal relationships that could have appeared to influence the work reported in this paper. The opinions expressed in this article are those of the authors and do not necessarily reflect the views of the Pan American Health Organization (PAHO), its Board of Directors, or the countries they represent.

Figure Legends

Figure 1. Overview of the study procedure for data collection and outcomes of interest

Figure 2. Power of the analysis as a function of the number of cases for different probabilities of exposure to vaccines from 20% to 50% for the detection of an OR=1.3 (increased risk).

Figure 3. Power as a function of the number of cases for different probabilities of exposure to vaccines from 20% to 50% for the detection of an OR=0.7 (protection).

For peer review only - http://bmjopen.bmj.com/site/about/guidelines.xhtml

For peer review only

For peer review only - http://bmjopen.bmj.com/site/about/guidelines.xhtml

References

1. WHO. WHO SAGE Roadmap For Prioritizing Uses Of COVID-19 Vaccines In The Context Of Limited Supply - An approach to inform planning and subsequent recommendations based on epidemiological setting and vaccine supply scenarios (Version 16 July 2021) [Internet]. 19 July. 2021. Available from: https://reliefweb.int/report/world/who-sage-roadmap-prioritizing-uses-covid-19-vaccines-context-limited-supply-approach
2. Wei SQ, Bilodeau-Bertrand M, Liu S, Auger N. The impact of COVID-19 on pregnancy outcomes: a systematic review and meta-analysis. C Can Med Assoc J = J l'Association medicale Can. 2021 Apr;193(16):E540–8.
3. Adhikari EH, Spong CY. COVID-19 Vaccination in Pregnant and Lactating Women. JAMA [Internet]. 2021 Mar 16 [cited 2022 Jun 6];325(11):1039–40. Available from: https://pubmed.ncbi.nlm.nih.gov/33555297/
4. Allotey J, Stallings E, Bonet M, Yap M, Chatterjee S, Kew T, et al. Clinical manifestations, risk factors, and maternal and perinatal outcomes of coronavirus disease 2019 in pregnancy: living systematic review and meta-analysis. BMJ. 2020 Sep;370.
5. Di Mascio D, Sen C, Saccone G, Galindo A, Grünebaum A, Yoshimatsu J, et al. Risk factors associated with adverse fetal outcomes in pregnancies affected by Coronavirus disease 2019 (COVID-19): a secondary analysis of the WAPM study on COVID-19. J Perinat Med [Internet]. 2020 Jan 1 [cited 2022 Sep 21];49(1):111–5. Available from: https://pubmed.ncbi.nlm.nih.gov/33470966/
6. Ciapponi A, Bardach A, Comandé D, Berrueta M, Argento FJ, Cairoli FR, et al. COVID-19 and pregnancy: An umbrella review of clinical presentation, vertical transmission, and maternal and perinatal outcomes. PLoS One [Internet]. 2021 Jun 1 [cited 2022 Jun 6];16(6). Available from: https://pubmed.ncbi.nlm.nih.gov/34185807/
7. Akhtar H, Patel C, Abuelgasim E, Harky A. COVID-19 (SARS-CoV-2) Infection in Pregnancy: A Systematic Review. Gynecol Obstet Invest [Internet]. 2020 Oct 1 [cited 2022 Jun 6];85(4):295–306. Available from: https://pubmed.ncbi.nlm.nih.gov/32728006/

For peer review only

8. Beigi RH, Krubiner C, Jamieson DJ, Lyerly AD, Hughes B, Riley L, et al. The need for inclusion of pregnant women in COVID-19 vaccine trials. Vaccine [Internet]. 2021 Feb 5 [cited 2022 Jun 6];39(6):868–70. Available from: https://pubmed.ncbi.nlm.nih.gov/33446385/

9. Macias Saint-Gerons D, Solà Arnau I, De Mucio B, Arévalo-Rodríguez I, Alemán A, Castro JL, et al. Adverse events associated with the use of recommended vaccines during pregnancy: An overview of systematic reviews. Vaccine. 2021 Jul;39 Suppl 2:B12–26.

10. Collier ARY, McMahan K, Yu J, Tostanoski LH, Aguayo R, Ansel J, et al. Immunogenicity of COVID-19 mRNA Vaccines in Pregnant and Lactating Women. JAMA [Internet]. 2021 Jun 15 [cited 2022 Jun 6];325(23):2370–80. Available from: https://pubmed.ncbi.nlm.nih.gov/33983379/

11. Beharier O, Mayo RP, Raz T, Sacks KN, Schreiber L, Suissa-Cohen Y, et al. Efficient maternal to neonatal transfer of antibodies against SARS-CoV-2 and BNT162b2 mRNA COVID-19 vaccine. J Clin Invest [Internet]. 2021 Oct 1 [cited 2022 Jun 6];131(19). Available from: https://pubmed.ncbi.nlm.nih.gov/34596052/

12. Bowman CJ, Bouressam M, Campion SN, Cappon GD, Catlin NR, Cutler MW, et al. Lack of effects on female fertility and prenatal and postnatal offspring development in rats with BNT162b2, a mRNA-based COVID-19 vaccine. Reprod Toxicol [Internet]. 2021 Aug 1 [cited 2022 Jun 6];103:28–35. Available from: https://pubmed.ncbi.nlm.nih.gov/34058573/

13. Shimabukuro TT, Kim SY, Myers TR, Moro PL, Oduyebo T, Panagiotakopoulos L, et al. Preliminary Findings of mRNA Covid-19 Vaccine Safety in Pregnant Persons. N Engl J Med [Internet]. 2021 Jun 17 [cited 2022 Jun 1];384(24):2273–82. Available from: https://www.nejm.org/doi/full/10.1056/NEJMoa2104983

14. Goldshtein I, Steinberg DM, Kuint J, Chodick G, Segal Y, Shapiro Ben David S, et al. Association of BNT162b2 COVID-19 Vaccination During Pregnancy With Neonatal and Early Infant Outcomes. JAMA Pediatr [Internet]. 2022 [cited 2022 May 4];176(5). Available from: https://pubmed.ncbi.nlm.nih.gov/35142809/

15. Kachikis A, Englund JA, Singleton M, Covelli I, Drake AL, Eckert LO. Short-term Reactions Among Pregnant and Lactating Individuals in the First Wave of the COVID-19 Vaccine Rollout. JAMA Netw open [Internet]. 2021 [cited 2022 Jun

For peer review only

1];4(8). Available from: https://pubmed.ncbi.nlm.nih.gov/34402893/

16. Magnus MC, Örtqvist AK, Dahlqwist E, Ljung R, Skår F, Oakley L, et al. Association of SARS-CoV-2 Vaccination During Pregnancy With Pregnancy Outcomes. JAMA [Internet]. 2022 Apr 19 [cited 2022 Jun 6];327(15):1469. Available from: https://pubmed.ncbi.nlm.nih.gov/35323851/

17. Zauche LH, Wallace B, Smoots AN, Olson CK, Oduyebo T, Kim SY, et al. Receipt of mRNA Covid-19 Vaccines and Risk of Spontaneous Abortion. N Engl J Med [Internet]. 2021 Oct 14 [cited 2022 Sep 22];385(16):1533–5. Available from: https://pubmed.ncbi.nlm.nih.gov/34496196/

18. Falsaperla R, Leone G, Familiari M, Ruggieri M. COVID-19 vaccination in pregnant and lactating women: a systematic review. Expert Rev Vaccines [Internet]. 2021 [cited 2022 Sep 22];20(12):1619–28. Available from: https://pubmed.ncbi.nlm.nih.gov/34592123/

19. Magnus MC, Gjessing HK, Eide HN, Wilcox AJ, Fell DB, Håberg SE. Covid-19 Vaccination during Pregnancy and First-Trimester Miscarriage. N Engl J Med [Internet]. 2021 Nov 18 [cited 2022 Sep 22];385(21):2008–10. Available from: https://pubmed.ncbi.nlm.nih.gov/34670062/

20. Prasad S, Kalafat E, Blakeway H, Townsend R, O'Brien P, Morris E, et al. Systematic review and meta-analysis of the effectiveness and perinatal outcomes of COVID-19 vaccination in pregnancy. Nat Commun [Internet]. 2022 Dec 1 [cited 2022 Jul 5];13(1). Available from: https://pubmed.ncbi.nlm.nih.gov/35538060/

21. Magee LA, von Dadelszen P, Kalafat E, Duncan EL, O'Brien P, Morris E, et al. COVID-19 vaccination in pregnancy-number needed to vaccinate to avoid harm. Lancet Infect Dis [Internet]. 2021 Dec 1 [cited 2022 Jun 6];21(12):1627. Available from: https://pubmed.ncbi.nlm.nih.gov/34739865/

22. Kalafat E, Magee LA, von Dadelszen P, Heath P, Khalil A. COVID-19 booster doses in pregnancy and global vaccine equity. Lancet (London, England) [Internet]. 2022 Mar 5 [cited 2022 Jun 6];399(10328):907–8. Available from: https://pubmed.ncbi.nlm.nih.gov/35189078/

23. Stock SJ, Carruthers J, Calvert C, Denny C, Donaghy J, Goulding A, et al. SARS-CoV-2 infection and COVID-19 vaccination rates in pregnant women in Scotland.

For peer review only - http://bmjopen.bmj.com/site/about/guidelines.xhtml

Nat Med [Internet]. 2022 Mar 1 [cited 2022 Jun 6];28(3):504–12. Available from: https://pubmed.ncbi.nlm.nih.gov/35027756/

24. Engjom H, van den Akker T, Aabakke A, Ayras O, Bloemenkamp K, Donati S, et al. Severe COVID-19 in pregnancy is almost exclusively limited to unvaccinated women - time for policies to change. Lancet Reg Heal Eur [Internet]. 2022 Feb 1 [cited 2022 Sep 22];13. Available from: https://pubmed.ncbi.nlm.nih.gov/35098198/

25. Kharbanda EO, Haapala J, Desilva M, Vazquez-Benitez G, Vesco KK, Naleway AL, et al. Spontaneous Abortion Following COVID-19 Vaccination During Pregnancy. JAMA [Internet]. 2021 Oct 26 [cited 2022 Jun 6];326(16):1629–31. Available from: https://pubmed.ncbi.nlm.nih.gov/34495304/

26. Centers for disease control and prevention (CDC). COVID-19 Vaccines While Pregnant or Breastfeeding [Internet]. 2022 [cited 2022 Nov 23]. Available from: https://www.cdc.gov/coronavirus/2019-ncov/vaccines/recommendations/pregnancy.html#:~:text=COVID-19 vaccination is recommended,become pregnant in the future.

27. WHO. Questions and Answers: COVID-19 vaccines and pregnancy [Internet]. WHO/2019-nCoV/FAQ/Pregnancy/Vaccines/2022.1. 2022 [cited 2022 Sep 22]. p. 1–5. Available from: https://www.who.int/publications/i/item/WHO-2019-nCoV-FAQ-Pregnancy-Vaccines-2022.1

28. Kalafat E, O'Brien P, Heath PT, Le Doare K, von Dadelszen P, Magee L, et al. Benefits and potential harms of COVID-19 vaccination during pregnancy: evidence summary for patient counseling. Ultrasound Obstet Gynecol [Internet]. 2021 May 1 [cited 2022 Jun 6];57(5):681–6. Available from: https://pubmed.ncbi.nlm.nih.gov/33734524/

29. Stafford IA, Parchem JG, Sibai BM. The coronavirus disease 2019 vaccine in pregnancy: risks, benefits, and recommendations. Am J Obstet Gynecol [Internet]. 2021 May 1 [cited 2022 Jun 6];224(5):484–95. Available from: https://pubmed.ncbi.nlm.nih.gov/33529575/

30. Blakeway H, Prasad S, Kalafat E, Heath PT, Ladhani SN, Le Doare K, et al. COVID-19 vaccination during pregnancy: coverage and safety. Am J Obstet Gynecol [Internet]. 2022 Feb 1 [cited 2022 Jun 6];226(2):236.e1-236.e14. Available from:

For peer review only

https://pubmed.ncbi.nlm.nih.gov/34389291/

31. Gomez Ponce de Leon R, Baccaro LF, Rubio Schweizer G, Bahamondes V, Messina A, de Francisco LA, et al. Building a network of sentinel centres for the care of women in an abortion situation: advances in Latin America and the Caribbean. BMJ Glob Heal. 2022 Oct;7(10).
32. Organization WH. Evaluating the quality of care for severe pregnancy complications: the WHO near-miss approach for maternal health. Geneva: WHO Press; 2011.
33. Dupont WD. Power Calculations for Matched Case-Control Studies. Biometrics. 1988 Dec;44(4):1157.
34. Pearce N. Analysis of matched case-control studies. BMJ [Internet]. 2016 Feb 25 [cited 2022 Sep 22];352. Available from: https://www.bmj.com/content/352/bmj.i969
35. Dupont WD, Plummer WDJ. Power and sample size calculations. A review and computer program. Control Clin Trials. 1990 Apr;11(2):116–28.
36. Zambrano LD, Ellington S, Strid P, Galang RR, Oduyebo T, Tong VT, et al. Update: Characteristics of Symptomatic Women of Reproductive Age with Laboratory-Confirmed SARS-CoV-2 Infection by Pregnancy Status — United States, January 22–October 3, 2020. MMWR Morb Mortal Wkly Rep. 2020 Nov;69(44):1641–7.
37. World Medical Association Declaration of Helsinki: ethical principles for medical research involving human subjects. JAMA [Internet]. 2013 Nov 27 [cited 2022 Jun 7];310(20):2191–4. Available from: https://pubmed.ncbi.nlm.nih.gov/24141714/
38. CIOMS. International Ethical Guidelines for Epidemiological Studies. Geneva; 2009. 130 p.
39. Langan SM, Schmidt SA, Wing K, Ehrenstein V, Nicholls SG, Filion KB, et al. The reporting of studies conducted using observational routinely collected health data statement for pharmacoepidemiology (RECORD-PE). BMJ [Internet]. 2018 [cited 2022 Jun 7];363. Available from: https://pubmed.ncbi.nlm.nih.gov/30429167/

For peer review only

For peer review only

For peer review only - http://bmjopen.bmj.com/site/about/guidelines.xhtml

TABLES

Table 1. Characteristics of the hospitals that use the SIP-PLUS database

Country	Income Level	Name of Hospital	Level	Deliveries attended per year
Argentina	UMIC	Martin Maternity Hospital, Rosario	Tertiary	3600
		Roque Sáenz Peña, Rosario		2500
		Eva Perón, Rosario		1500
Bolivia	LMIC	Bolivian-Japanese Hospital	Tertiary	2719
		La Paz Maternity Hospital	Tertiary	1500
Colombia	UMIC	Rafael Calvo Maternity Clinic	Secondary	7000
		Hospital Universitario del Valle, Cali	Tertiary	7200
Ecuador	UMIC	Isidro Ayora Maternity Hospital	Tertiary	7000
Honduras	LMIC	Roberto Suazo Cordova Hospital	Secondary	3201
		San Felipe Hospital	Secondary	6128
		Leonardo Martínez Valenzuela	Tertiary	18000
Nicaragua	LMIC	España Hospital	Secondary	5250
		Berta Calderón Roque Hospital	Secondary	8993
Dominican Republic	UMIC	San Lorenzo de Los Mina Hospital	Tertiary	13000
Uruguay	UMIC	Pereira Rosell Hospital Center	Tertiary	5000
		Hospital de Clinicas Manual Quintela	Tertiary	800

LMIC: low- and middle-income country; UMIC: upper-middle-income country.

For peer review only

For peer review only

For peer review only - http://bmjopen.bmj.com/site/about/guidelines.xhtml

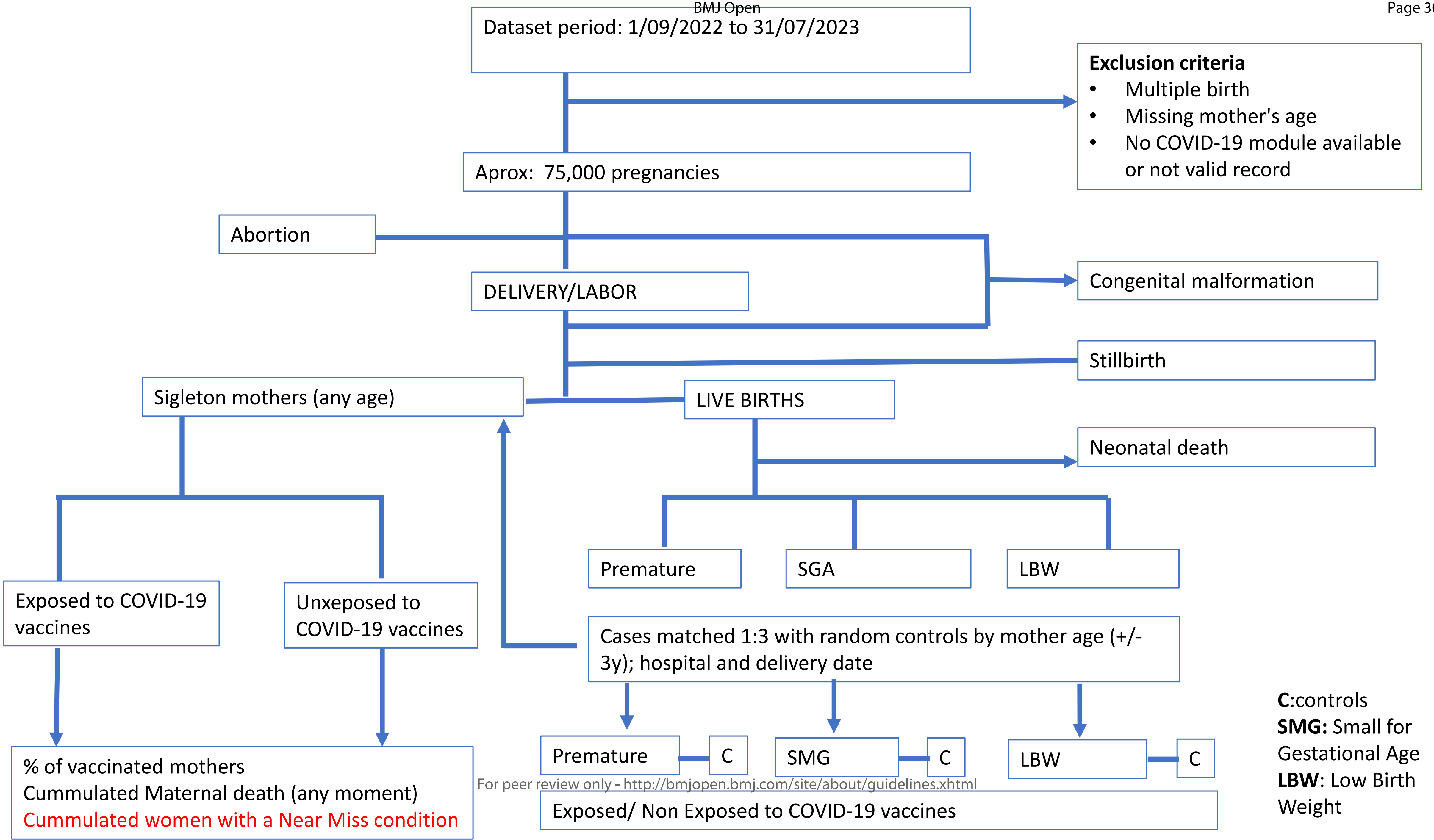
Dataset period: 1/09/2022 to 31/07/2023
Exclusion criteria
• Multiple birth
• Missing mother's age
• No COVID-19 module available or not valid record
Aprox: 75,000 pregnancies
Abortion
DELIVERY/LABOR
Congenital malformation
Stillbirth
Sigleton mothers (any age)
LIVE BIRTHS
Neonatal death
Premature
SGA
LBW
Exposed to COVID-19 vaccines
Unexposed to COVID-19 vaccines
Cases matched 1:3 with random controls by mother age (+/-3y); hospital and delivery date
% of vaccinated mothers
Cummulated Maternal death (any moment)
Cummulated women with a Near Miss condition
Premature
C
SMG
C
LBW
C
Exposed/ Non Exposed to COVID-19 vaccines
C:controls
SMG: Small for Gestational Age
LBW: Low Birth Weight

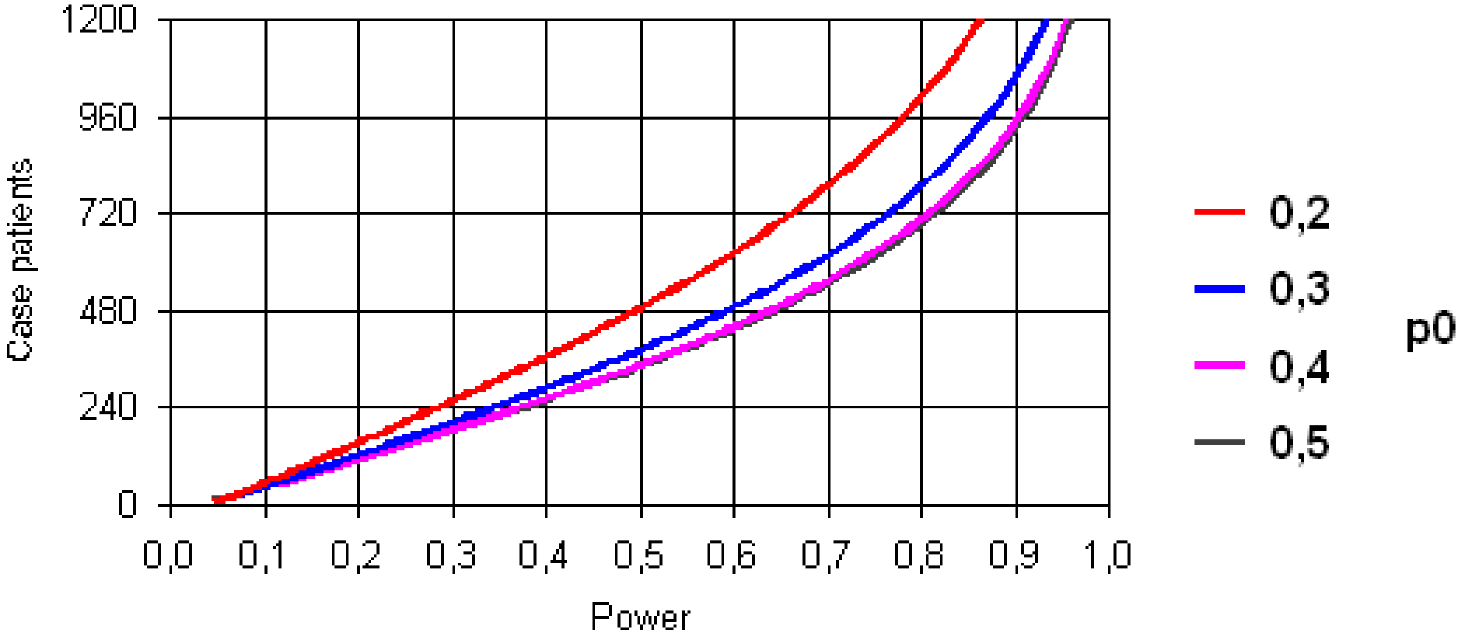
1200
960
720
480
240
0
Case patients
0,0
0,1
0,2
0,3
0,4
0,5
0,6
0,7
0,8
0,9
1,0
Power
0,2
0,3
0,4
0,5
p0

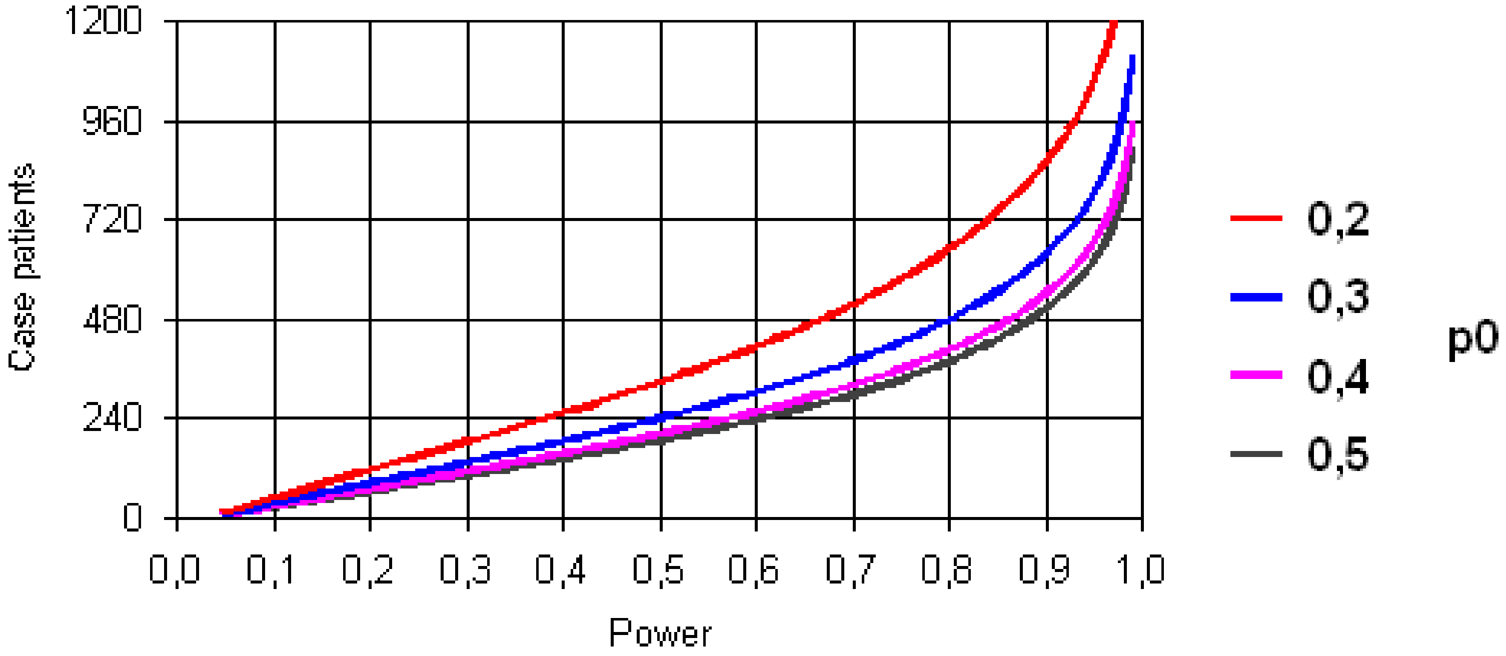
1200
960
720
480
240
0
Case patients
0,0
0,1
0,2
0,3
0,4
0,5
0,6
0,7
0,8
0,9
1,0
Power
0,2
0,3
0,4
0,5
p0

This color means WATCH (does not necessarily indicate risk or inadequate practices)

PERINATAL CLINICAL RECORD - CLAP/WR-PAHO/WHO

FIRST NAME | LAST NAME
ADDRESS
DISTRICT | PHONE.

DATE OF BIRTH day month year
AGE (years) < 15 > 35
ETHNICITY: white, indigenous, mixed, black, other
LITERACY: no yes
EDUCATION: none, primary, secondary, university; years in highest level
CIVIL STATUS: married, common mar., single, other; lives alone no yes
Place of antenatal visits; Place of birth; ID #

HISTORY

FAMILY no yes | PERSONAL no yes
TBC (I II G); diabetes; hypertension; pre-eclampsia; eclampsia; other severe med. cond.
surgery of UT-UG no yes; infertility; heart dis.; kidney dis.; violence; HIV+

OBSTETRIC: prev. gest; abortions; vaginal; livebirths; alive
LAST PREVIOUS: n/a, < 2500g; normal, ≥ 4000g
3 consec. spont. ab.; tubal pregnancy; deliveries; cesar. sections; stillbirths; died 1st week; died after 1st week
History of twins no yes
END PREVIOUS PREGNANCY day month year; < 1 year
PLANNED PREGNANCY no yes
CONTRACEP. METHOD FAILURE: no method, barrier, IUD, horm., emerg., natural

CURRENT PREGNANCY

PREVIOUS WEIGHT; HEIGHT (cm); Kg
DLM day month year; EDD day month year
GA BASED on DLM, Us <20 w. no yes
ACT SMO, PAS SMO, DRUGS, ALCOHOL, VIOLENCE (no yes) — 1st trim, 2nd trim, 3rd trim
VACCINES: no; before pregnancy; during pregnancy; after delivery/abortion; day month year; number of doses; gest. age weeks
tetanus diphtheria; tdap; influenza; rubella; hepatitis B; hepatitis A
screening hepatitis B no yes

NORMAL EX. no yes: DENTAL; BREAST
CERVIX normal abnormal not done: Visual insp; PAP; COLP
GROUP; Rh − +; Inmun no yes; γglobulina anti D no yes n/a
TOXOPLASMOSIS − + not done: <20 week IgG; ≥20 week IgG; 1 visit IgM
Hb <20 wk: g <11.0 g/dl
Presciption Fe/Folates: Fe, Folates; no yes
Hb ≥20 wk: g <11.0 g/dl

CHAGAS − + not done
MALARIA − + not done
BACTERIURIA normal abnormal not done: wk <20; ≥20
FASTING BLOOD SUGAR: <20 wk mg/dl; ≥30 wk mg/dl
G.B.S 35-37 weeks − + not done
PREPARING FOR CHILDBIRTH no yes
ADVICE ON BREAST-FEEDING no yes

HIV - Diag - Treatment: <20 weeks request (yes, no, n/a), test result (+, −, n/d, n/a); ART in preg. (yes, no, n/a); ≥20 weeks request (Si, No, n/a), test result (+, −, n/d, n/a); ART in preg. (yes, no, n/a)

SYPHILIS Diagnosis and treatment: Test no treponemic (− + n/d), treponemic (− + n/d n/a), <20 wk; weeks; Treatment (no yes n/d n/a) weeks; Treat. partner (no yes n/d n/a); Test no treponemic (− + n/d) ≥20 wk, treponemic (− + n/d n/a) weeks; Treatment (no yes n/d n/a) weeks; Treat. partner (no yes n/d n/a)

PRENATAL VISITS

day / month / year	gest age	weight	BP	uterine height	presentation	FHR (bpm)	fetal movements	proteinuria	place of control	alarm signs, test, treatments	Technician's initials	next visit
		,										
		,										
		,										
		,										
		,										

CHILDBIRTH / ABORTION

DATE OF ADMISSION day month year
CARD no yes
PRENATAL VISITS total
AGE GEST. 1st CONSULT. weeks
HOSPITAL during PREGNANCY no yes days
ANTENATAL STEROIDS: complete, incompl., no, n/a; week started
ONSET: spontan., induced, elect. ces.
ANTEPARTUM RUPT. OF MEMBRANES: no yes; day month year; hour min; <37 wk; ≥18 hs.; temp. ≥38ºC
GEST AGE AT CHILDBIRTH weeks days; by LMP, by US
PRESENTATION SITUATION: cephalic, breech, transverse
FETAL SIZE ADEQUATE no yes
COMPANION labor del: couple, relative, other, no one

LABOR

details in partograph yes no

hour	min	mother position	BP	pulse	contr./10'	dilatation	height present.	variety posit.	mecon	FHR/dips

DISEASES — Labor Test − + n/d n/a: Syphilis; HIV; ART yes no n/a

BIRTH

ALIVE; DEAD antepartum, intra partum, not known when
hour min; day month year
MULTIPLE order no yes
TERMINATION: spont., forceps, c-sect, vacuum, other
MAIN INDICATION FOR INDUCTION OR SURGERY; INDUC. SURG; code
POSITION LABOR: sitting, recumb, squat
EPISIOTOMY no yes
TEARS Grade (1 to 4) no
OXYTOCIC before expul. plac. no yes; after no yes
PLACENTA no yes: complete, retained
EARLY CORD CLAMPING < 1 min., 1 to 3 min.
MEDICATION RECEIVED: oxytocic at labor, ATB, analges, local anesth, region anesth, gral anesth, transfusion, Mg sulfate preeclam. eclam. — no / yes; others (code) medic 1, medic 2

NEONATE

SEX f m not defined
BIRTH WEIGHT g; <2500 g; ≥4000 g
HEAD CIRC cm; LENGTH cm
GESTATIONAL AGE weeks days; LMP; US; ESTIMATED
WEIGHT G.A. adeq.; small; large
APGAR (min) 1st 5th
Early intitiation of breast feeding no yes
RESUSCITATION no yes: stimul., suction, mask, oxygen, massage, tube, medication
ATTENDED: doctor, obst., nuerse, assist., stud., TBA, other, Name — CHILDBIRTH; NEONATE

CONGENITAL DEFECTS no, minor, major; code
DISEASES: 1 o more, none; code
NEONATAL SCREENING: HIV in NN — Exposed no yes s/d; Treated no yes s/d n/a
VDRL − + not done; Tx no yes n/a s/d
Hearing; Chagas disease; Bili rrub; Toxo IgM; Hb pathy; Cardio-vascular disease — − + not done
Metabolic; not done; done
Death at site of birth no yes: Mother; NN
REFERRAL: room in, neona tol, another hosp.

POSTPARTUM

time	Temperature ºC	B.P.	pulse	uterine involution	Bleeding	Responsible
	,					
	,					
	,					
	,					
	,					

γglobulin anti D no yes n/a

NEONATAL DISCHARGE

alive; dead; day month year; hour min
transfer; place
dies during or at transfer no yes s/d
AGE AT DISCHARGE complete day; <1 day
FEEDING AT DISCH: excl. breastf, partial, artificial
Supine no yes; BCG no yes; INMUN. HEPATITIS B no yes
Meconium 1st day no yes
WEIGHT AT DISCHARGE
Id. NN; Name NN; Responsabile

HCP NEAR MISS ANV_ENG 02-08-201087

M O R B I L I T Y

HYPERTENSIVE DISORDERS no yes

	no	yes
Chronic hypertension	○	○
Mild preeclampsia	○	○
Severe preeclampsia	○	○
Eclampsia	○	○
HELLP	○	○
Gestational Hypertension	○	○
Chronic hypertension + preeclampsia	○	○

INFECTIONS no yes

	no	yes		no	yes
Sepsis	○	○	Pyelonephritis	○	○
Endometritis	○	○	Pneumonia	○	○
Chorioamnionitis	○	○	Caesarean section wound infection	○	○
Asymptomatic bacteriuria	○	○	Episiorrhaphy infection	○	○
			Other infections	○	○

HEMORRHAGE

1st TRIMESTER no yes | 2nd TRIMESTER no yes | 3rd TRIMESTER no yes

	no	yes		no	yes
Post abortion	○	○	Uterine rupture	○	○
Hydatidiform Mole	○	○	Postpartum hemorrhage	○	○
Ectopic pregnancy	○	○	Uterine atony	○	○
Previous placenta	○	○	Lacerations	○	○
Accretism	○	○	Ovular remains	○	○
Abruptio placentae	○	○	Coagulation defect	○	○

METABOLIC DISORDERS no yes

MELLITUS DIABETES no yes

	no	yes	not performed
Abnormal oral tolerance to glucose load	○	○	○
Previous M. D. (insulin dependent)	○	○	
Previous M. D. (non insulin dependent)	○	○	
Gestational DM	○	○	
Hyperosmolar state	○	○	
Cetoacidosis	○	○	
Hyperglycemic state	○	○	

THYROID DISORDERS no yes

	no	yes
Hypothyroidism	○	○
Hyperthyroidism	○	○
Thyroid crisis	○	○
Another metabolic disorder	○	○

OTHER DISORDERS no yes

	no	yes		no	yes
Hyperemesis gravidarum	○	○	Oliguria	○	○
Deep vein thrombosis	○	○	Anemia	○	○
Pulmonary embolism	○	○	Sickle cell anemia	○	○
Amniotic fluid embolism	○	○	Renal disease	○	○
Heart disease	○	○	Malignant neoplasia	○	○
Valvulopathy	○	○	Psychiatric disorder	○	○
Seizures	○	○	Cholestasis	○	○
Altered state of consciousness	○	○	Others	○	○

OBSTETRIC COMPLICATIONS no yes

	no	yes		no	yes
Obstructed labor	○	○	Polyhydramnios	○	○
Prolonged rupture of membranes	○	○	Acute fetal distress	○	○
Oligoamnios	○	○	Intrauterine growth restriction	○	○
Other	○	○			

INTERVENTIONS

	no	yes	s/d
Manual removal	○	○	○
Uterotonic to treatment of hemorrhag	○	○	○

Which ones?

	no	yes	s/d
Central venous catheter	○	○	○
Administration of blood products	○	○	○

Nº of vol.

	no	yes	s/d
Laparotomy (excluding caesarean section)	○	○	○
ICU admission <7 days	○	○	○
Using ATB iv to treatment of infectious complication	○	○	○

days

Which ones?

	no	yes
No pneumatic shock-suit	○	○
Hydrostatic balls	○	○
B-Lynch suture or similar	○	○
Hemostatic ligature of uterine / hypogastric arteries	○	○
Embolizations	○	○

VARIABLES TO IDENTIFY NEAR MISS CASES no yes

CLINICAL CRITERIA / BODY-SYSTEM DYSFUNCTION

	no	yes
Cardiovascular		
Shock	○	○
Heart attack	○	○
Hepatic		
Jaundice in preeclampsia	○	○
Respiratory		
Acute cyanosis	○	○
Wheezing (gasping)	○	○
Severe tachypnea RF> 40 rpm	○	○
Severe bradypnea RF <6 rpm	○	○

	no	yes
Renal		
Oliguria non responsive to fluids or diuretics	○	○
Hematological/coagulation		
Failure to form clots	○	○
Neurological		
Coma	○	○
Prolonged unconsciousness (lasting >12 hours	○	○
Stroke	○	○
Uncontrollable fits/status epileptics	○	○
Total paralysis	○	○

LAB

Laboratory	no	yes
Platelets/ml < 50.000	○	○
Creatinine ≥ 300 µmol/l or ≥ 3,5 mg/dl	○	○
Bilirubin > 100 µmol/l or > 6,0 mg/dl	○	○
pH < 7,1	○	○
Sat Hb < 90% ≥ 1 hour	○	○
PaO_2/FiO_2 < 200 mmHg	○	○
Lactato > 5 µmol/L o 45 mg/dl	○	○

INTERVENTIONS

	no	yes
Use of continuous vasoactive drug	○	○

Which ones?

	no	yes
Intubation and ventilation not related to anesthesia	○	○

days

Criteria Near Miss: FLASOG ≥ 3 vol. WHO ≥ 5 vol.

	no	yes
Massive transfusion of blood or red cells (≥ 3 units)	○	○
Admission to UCI ≥ 7 days	○	○
Histerectomy	○	○
Dialysis for acute renal failure	○	○
Cardiopulmonary resucitation	○	○

days

PUERPERIUM

day	Temp ºC	blood prasure	pulse	uter. invol.	lochia	perine	breast feeding	observations	Responsabile
1st	,								
2nd	,								
3rd	,								
5th to 10th	,								

MATERNAL DISCHARGE

DATE: day | month | year — hour | min

TYPE OF DISCHARGE: dead ○ against medical advise ○ discharge ○

CONDITION AT DISCHARGE: healthy ○ NA ○ with pathology ○ dead ○

Place | Transfer | passes away during or after transfer no ○ yes ○ | Autopsy no ○ yes ○

Responsabile

Contraception councelling: oral ○ written ○ none ○

Responsabile

CONTRACEPTION

Start contraception	no ○	yes ○	IUD	pref. ○	acced. ○	injectable	pref. ○	acced. ○	barrier	pref. ○ acced. ○
tube ligation	pref. ○	acced. ○								
hormonal contraception	pref. ○	acced. ○	Other hormonal (vaginal ring, patch)	pref. ○	acced. ○	male surgical sterilization	pref. ○	acced. ○	hormonal implant	pref. ○ acced. ○
condom	pref. ○	acced. ○	abstinence	pref. ○	acced. ○					

This color means WATCH (does not necessarily indicate risk or inadequate practices)

HCP NEAR MISS REVERSO (Regional) INGLES 06-08-2018

For peer review only

For peer review only - http://bmjopen.bmj.com/site/about/guidelines.xhtml

For peer review only

NAME

Institution

LAST NAME

Country code

Type of doc.

ID number

Had COVID-19 in the 12 months before the pregnancy termination?

	yes	no	Date (month / day / year)
Before pregnancy			month / day / year
During pregnancy 1st. contagion			month / day / year
During pregnancy 2nd contagion			month / day / year

Diagnostic test

	PCR	Rapid	doesn't know
Before pregnancy			
During pregnancy 1st. contagion			
During pregnancy 2nd contagion			

Indicate the greater severity of the contagion(s) during pregnancy

	Asymptomatic - home	Symptomatic - home	Inpatient – intermediate	Inpatient – intensive
During pregnancy 1st. contagion				
During pregnancy 2nd contagion				

Did she receive any dose of the COVID-19 vaccine in the last year? yes no **(If yes, indicate from the most recent to the oldest)**

Date	Gestational age	Did she present certificate/card? yes	no	AstraZeneca (AZD1222, ChAdOx1 nCoV-19, ChAdOx1-S, Vaxzevria, Covishield)	Janssen (JNJ-78436735; Ad26.COV2-S)	Pfizer/BioNtech (BNT162b2; Tozinameran; Comirnaty)	Sputnik V (Gamaleya; Gam-COVID-Vac; Adeno-based (rAd26-S+rAd5-S))	Sinopharm/ BIBP (Sinopharm/BIB)	Sinovac (CoronaVac; VeroCell)	Moderna (mRNA-1273; Elasomeran)	doesn't know	Other
month / day / year	or											Which one?:
month / day / year	or											Which one?:
month / day / year												Which one?:

For peer review only - http://bmjopen.bmj.com/site/about/guidelines.xhtml

Supplemental material III. Births attended and estimated number of potential cases per year.

Country	Hospital Name	Deliveries per year	Number of potential events per year		
			Small for gestational age	Preterm	Low birth weight
Bolivia	Hospital Boliviano Japonés	2719	177	337	185
	Maternidad de la Paz	1500	98	186	102
Colombia	Clínica Maternidad Rafael Calvo	7000	455	868	476
	Hospital Universitario del Valle, Cali	7200	468	893	497
Honduras	Hospital Roberto Suazo Cordova; Hospital de San Felipe; Leonardo Martinez Valenzuela; Hospital Berta Calderón Roque	27300	1775	3385	1856
República Dominicana	Hospital San Lorenzo de Los Mina	13000	845	1612	884
Argentina	Maternidad Martin	4200	273	349	291
	Roque Saenz Peña	2500	163	310	170
	Eva Peron	1500	98	186	102
Ecuador	Isidro Ayora	7000	455	868	476
Uruguay	Centro hosp Pereira Rosell	5000	325	620	340
	Hospital de Clinicas	800	52	99	54
Total SIP PLUS	-	93962	5731	10932	5995

For peer review only

BMJ Open

Description of maternal and neonatal adverse events in pregnant people immunized with COVID-19 vaccines during pregnancy in the CLAP NETWORK of sentinel sites. Nested case-control analysis of the immunization-associated risk: A study protocol

Journal:	*BMJ Open*
Manuscript ID	bmjopen-2023-073095.R1
Article Type:	Protocol
Date Submitted by the Author:	20-Jul-2023
Complete List of Authors:	Macías Saint-Gerons, Diego ; Department of Medicine, University of Valencia, INCLIVA Health Research Institute and CIBERSAM, Department of Medicine; PAHO<br>Castro, José ; Fundación para la innovación, la formación, la investigación y el desarrollo comunitario (FUNDEC); PAHO<br>Colomar, Mercedes; Unidad de Investigación Clínica y Epidemiológica Montevideo; PAHO, Latin American Center of Perinatology, Women and Reproductive Health (CLAP/WR)<br>Rojas, Robin; PAHO<br>Sosa, Claudio; Universidad de la Republica Uruguay, Department of Obstetrics and Gynecology, Pereira Rossell Hospital, School of Medicine; PAHO, Latin American Center of Perinatology, Women and Reproductive Health (CLAP/WR)<br>Ropero, Alba ; PAHO<br>Serruya, Suzanne; PAHO, Latin American Center of Perinatology, Women and Reproductive Health (CLAP/WR)<br>Pastor, Desiré; PAHO<br>Chiu, Monica; PAHO<br>Velandia-Gonzalez, Martha; PAHO<br>Abalos, Edgardo; Centro de Estudios de Estado y Sociedad (CEDES)<br>Durán, Pablo; PAHO, Latin American Center of Perinatology, Women and Reproductive Health (CLAP/WR)<br>Gomez Ponce de León, Rodolfo; PAHO, Latin American Center of Perinatology, Women and Reproductive Health (CLAP/WR)<br>Tomasso, Giselle; Unidad de Investigación Clínica y Epidemiológica Montevideo; PAHO, Latin American Center of Perinatology, Women and Reproductive Health (CLAP/WR)<br>Mainero, Luis; PAHO, Latin American Center of Perinatology, Women and Reproductive Health (CLAP/WR)<br>Rubino, Marcelo; PAHO, Latin American Center of Perinatology, Women and Reproductive Health (CLAP/WR)<br>De Mucio, Bremen; PAHO, Latin American Center of Perinatology, Women and Reproductive Health (CLAP/WR)
**Primary Subject Heading**:	Infectious diseases

Secondary Subject Heading:	Obstetrics and gynaecology, Public health
Keywords:	PERINATOLOGY, COVID-19, Maternal medicine < OBSTETRICS

SCHOLARONE™ Manuscripts

Description of maternal and neonatal adverse events in pregnant people immunized with COVID-19 vaccines during pregnancy in the CLAP NETWORK of sentinel sites. Nested case-control analysis of the immunization-associated risk: A study protocol.

Diego Macías Saint-Gerons (0000-0002-2572-2160), José Luis Castro (0000-0002-9179-113X), Mercedes Colomar (0000-0001-7424-5551), Robin Rojas (0000-0002-4046-9298), Claudio Sosa (0000-0002-2539-0848), Alba Maria Ropero (0000-0001-9960-7491), Suzanne Serruya (0000-0003-1371-4558), Desiré Pastor, Monica Chiu (0000-0002-2730-8625), Martha Velandia (0000-0002-7636-3604), Edgardo Abalos (0000-0001-6653-429X), Pablo Durán (0000-0003-1032-487X), Rodolfo Gómez Ponce de León (0000-0001-9914-7130), Giselle Tomasso (0000-0002-4424-1581), Luis Mainero, Marcelo Rubino, Bremen de Mucio (0000-0003-0662-9742)

Department of Medicine, University of Valencia, INCLIVA Health Research Institute and CIBERSAM, Department of Medicine, Valencia, Comunitat Valenciana Diego Macías Saint-Gerons International PAHO consultant, FÜNDEC: Fundación para la innovación, la formación, la investigación y el desarrollo comunitario, Santa Cruz de Tenerife José Luis Castro International PAHO consultant, Montevideo Clinical and Research Unit. Hospital de Clinicas, 11300 Montevideo, Uruguay Mercedes Colomar International PAHO consultant,
Pan-American Health Organization (PAHO), 525 23rd St NW, Washington DC 20037, United States Robin Rojas Technical Officer, Universidad de la República Uruguay, Department of Obstetrics and Gynecology, Pereira Rossell Hospital, School of Medicine, 11300 Montevideo, Uruguay Claudio Sosa International PAHO consultant, Pan-American Health Organization (PAHO), 525 23rd St NW, Washington DC 20037, United States Alba Maria Ropero Regional Advisor on Immunization, Latin American Center of Perinatology, Women and Reproductive Health (CLAP/WR), Av. Brasil 2697, 11300 Montevideo, Uruguay Suzanne Serruya CLAP Director, Pan-American Health Organization (PAHO), 525 23rd St NW, Washington DC 20037, United States Desiré

For peer review only - http://bmjopen.bmj.com/site/about/guidelines.xhtml

Pastor Regional Immunization Advisor, Pan-American Health Organization (PAHO), 525 23rd St NW, Washington DC 20037, United States Monica Chiu International PAHO consultant, Pan-American Health Organization (PAHO), 525 23rd St NW, Washington DC 20037, United States Martha Velandia Advisor Immunization, Centro de Estudios de Estado y Sociedad (CEDES), Sánchez de Bustamante 27, Ciudad Autónoma de Buenos Aires, Argentina, Edgardo Abalos CLAP Associate researcher, Latin American Center of Perinatology, Women and Reproductive Health (CLAP/WR), Av. Brasil 2697, 11300 Montevideo, Uruguay Pablo Durán advisor, Latin American Center of Perinatology, Women and Reproductive Health (CLAP/WR), Av. Brasil 2697, 11300 Montevideo, Uruguay Rodolfo Gómez Ponce de León advisor, Montevideo Clinical and Research Unit. Hospital de Clinicas, 11300 Montevideo, Uruguay Giselle Tomasso International PAHO consultant, Latin American Center of Perinatology, Women and Reproductive Health (CLAP/WR), Av. Brasil 2697, 11300 Montevideo, Uruguay Luis Mainero IT expert, Latin American Center of Perinatology, Women and Reproductive Health (CLAP/WR), Av. Brasil 2697, 11300 Montevideo, Uruguay Marcelo Rubino data analyst, Latin American Center of Perinatology, Women and Reproductive Health (CLAP/WR), Av. Brasil 2697, 11300 Montevideo, Uruguay Bremen de Mucio Advisor.

Correspondence to: Mercedes Colomar; +59899114204; mcolomar@unicem-web.org; Julio Maria Sosa 2281, CP 11300, Montevideo, Uruguay.

Word count: 4690

Keywords:

Perinatology; Maternal Health; Pharmacovigilance; Infant, Premature Birth; Infant, Small for Gestational Age; Low Birth Weight; Pregnancy Outcome; COVID-19 vaccines.

Abstract

Introduction

Coronavirus disease is associated with higher morbi-mortality in pregnant people compared to non-pregnant people. At present, the benefits of maternal immunization

For peer review only - http://bmjopen.bmj.com/site/about/guidelines.xhtml

are considered to outweigh the risks and therefore vaccination is recommended at any time during pregnancy. However, additional information is needed on the safety of the vaccines in this population.

Methods and analysis

This a retrospective cohort nested case–control study in pregnant people who attended maternity hospitals from 8 Latin American and Caribbean countries. Hospital´s characteristics can be found in table 1. A perinatal electronic clinical history database with data from neonatal and obstetric monitoring will be used (12 months; approximately 75,000 deliveries). The proportion of pregnant people immunized with COVID-19 vaccines of the following maternal and neonatal events will be described: preterm infant, small for gestational age, low birth weight, stillbirth, neonatal death, congenital malformations, maternal near miss, and maternal death. Moreover, the risk of prematurity, small for gestational age, and low birth weight associated with exposure to COVID-19 vaccines will be estimated. Each case will be matched with 3 randomly selected controls at the same delivery date of each case (index date) and matched by hospital and mother's age (+/- 3 years). The estimated required sample size for the main analysis (exposure to any vaccine) concerning "non-use" is at least 1009 cases (3,027 controls) to detect an increased probability of vaccine-associated event risk of 30% (OR=1.3) and at least 650 cases (1,950 controls) to detect 30% protection (OR=0.7). Sensitivity and secondary analyses considering country, type of vaccine, exposure windows, and completeness of immunization will be reported.

Ethics and dissemination

The study protocol was reviewed by the Ethical Review Committee on Research of the Panamerican Health Organization. Patient informed consent was waived due to the retrospective design and the utilization of anonymized data (Ref. No: PAHOERC.0546.01. Results will be disseminated in Open Access journals.

Strengths and limitations of this study

- Pregnant people were excluded from the pivotal clinical trials of COVID-19 vaccines and most of the evidence from vaccine safety comes from high income country studies.

For peer review only - http://bmjopen.bmj.com/site/about/guidelines.xhtml

- This study represents an opportunity to generate complementary evidence of maternal, foetal and neonatal adverse events after administration of COVID-19 vaccines currently used in LAC countries for maternal immunization.
- The use of the SIP-PLUS database allows achieving a large sample size necessary to explore potential vaccine-associated discrete effects (beneficial or harmful) in a timely manner.
- As in any study using routinely collected data there are further potential limitations due to incomplete data records, inaccuracies (dates), unmeasured confounding factors, and missing data.
  The number of "non exposed" pregnant people could decrease over time and may introduce affect statistical power and interpretation.

Introduction

Pregnant people do not appear to have a higher susceptibility to SARS-CoV-2 coronavirus infection than nonpregnant people.[1] However, coronavirus disease 2019 (COVID-19) is associated with higher morbidity and mortality among pregnant people compared with non-pregnant people.[2,3] Mortality in pregnant people with COVID-19 could be 70% higher, and the risk of admission to intensive care units (ICU) or the need for mechanical ventilation would be three times higher compared to non-pregnant unvaccinated people of similar age.[3,4] Pregnant people with COVID-19 show an increased risk of occurrence of some adverse maternal-fetal outcomes such as increased risk of preterm delivery, preeclampsia, or miscarriage compared to pregnant people without COVID-19.[2] It has been observed that, in its most complicated or critical forms, COVID-19 infection is associated with prematurity, gestational diabetes, preeclampsia and low birth weight.[3] Neonates born to mothers with COVID-19 appear to have a higher probability of admission to a neonatal care unit (NCU), which could be attributed to a greater extent to the negative effect of COVID-19 on maternal health during gestation than to vertical transmission.[4–6] Besides other maternal, fetal, and neonatal complications associated with COVID-19 have also been reported. A systematic review described adverse events such as fetal distress, premature rupture of membranes, and other neonatal adverse events such as respiratory distress, gastrointestinal events, and fever. Maternal death reports have been more rare.[7]

For peer review only - http://bmjopen.bmj.com/site/about/guidelines.xhtml

After a global research effort to develop effective treatments and vaccines against COVID-19, vaccines became available on January 2021. It should be noted that a characteristic of the new COVID-19 vaccines is that they were developed from a range of different platforms: viral vector (adenoviral), mRNA platform, inactivated, attenuated viruses, being pregnancy an exclusion criterion in the main pivotal clinical trials undertaken for the authorization of these COVID-19 vaccines.[8] Besides, vaccines that are currently recommended for maternal immunization - and with which there is most experience - such as the influenza vaccine or the Tdap vaccine, do not use mRNA-based platforms as is the case with the Pfizer-BioNTech and Moderna vaccines.[9] However, despite being based on a newer platform, mRNA vaccines do not entail the risk of acquiring vaccination-induced infection, as might be the case with the use of attenuated/inactivated vaccines. On the other hand, they could be useful in pregnant people, as a robust transfer of the humoral response to the fetus has been observed with mRNA vaccines.[10,11] Furthermore, no remarkable adverse events have been detected in experimental genotoxicity studies conducted on rats.[12]

Although various clinical trials have been planned for the study of COVID-19 vaccines in pregnant people, most of the initiatives are based on the creation of registries of pregnant people exposed to the vaccines. In many cases, it is the adaptation of previous registries that had been created to study the effects of COVID-19 infection in pregnant people. In this respect, Moderna created a registry to monitor pregnancy outcomes in individuals who received their COVID-19 vaccine. PREGISTRY - for example - is a startup focused on improving maternal-fetal health, in collaboration with the Harvard School of Public Health (https://www.pregistry.com); aim to enroll 8,000 pregnant people in their registry to evaluate their obstetric, neonatal, and infant outcomes following COVID-19 immunization. In the UK, the UKOSS registry (https://www.npeu.ox.ac.uk/ukoss) is collecting data to allow the parallel nesting of cohort and case-control studies. The CDC's "v-safe" registry have reported preliminary results with observed frequencies of spontaneous abortion and fetal death in vaccinated pregnant people similar to the expected frequencies in the general population of pregnant people of the same age and without known comorbidities.[13]

For peer review only - http://bmjopen.bmj.com/site/about/guidelines.xhtml

Apart from these active registries that include pregnant people, there is little evidence from published studies with complete data on the effectiveness and safety of vaccination for COVID-19 in this population and information from surveillance systems, monitoring acute adverse events following immunization during pregnancy, did not find increased risk of clinically serious acute adverse in different populations groups.[14,15] One retrospective cohort study observed that, in pregnant people, vaccination with Pfizer-BioNTech was associated with a lower risk of SARS-CoV-2 infection compared to those who did not receive vaccination (HR:0.22; 95% CI: 0.11-0.43).[16] In another prospective cohort study, short-term adverse events associated with mRNA vaccines were similar among pregnant people to those occurring in nonpregnant people of the same childbearing age.[17] In another large comparative study involving 157,521 singleton pregnancies from the Swedish pregnancy and Norwegian childbirth electronic registries, no statistically significant differences were found in the risk for fetal death, small for gestational age rates, preterm, and low APGAR score value in mRNA and Pfizer-BioNTech vaccinated and unvaccinated subjects.[18] Also, the risk of miscarriage does not appear to be higher than expected among people immunized before or during pregnancy.[19,20] Moreover, in a retrospective cohort including more than 40.000 pregnant people, COVID-19 vaccines (mostly mRNA) were not associated with preterm birth or small-for-gestational-age at birth.[21] On the other hand, a case-control study using Norwegian registry data observed no statistically significant risk difference for first-trimester miscarriage in pregnant people exposed to COVID-19 vaccines (Pfizer-BioNTech, Moderna, Astra) and those not immunized.[22] A study investigating on the associations of COVID-19 vaccination throughout pregnancy with delivery and neonatal outcomes suggests that there is no associated risk between prenatal COVID-19 vaccination and adverse delivery and neonatal outcomes in a cohort sample from NYC.[23]A systematic review and meta-analysis included data from 23 studies and 117,552 pregnant people immunized mostly with mRNA vaccines. In this meta-analysis, which accumulates the most evidence on the subject to date, no differences were found in vaccinated compared to unvaccinated pregnant people with regards the risk of occurrence of miscarriage, earlier gestation at birth, placental abruption, pulmonary embolism, postpartum hemorrhage, maternal death, mothers' admission to the ICU, low birth weight, or newborns' admission to the ICU. The meta-analysis identified that

For peer review only - http://bmjopen.bmj.com/site/about/guidelines.xhtml

immunization conferred a 15% protective effect against fetal death, although the heterogeneity found was very high (Odds Ratio: 0.85; 95%CI: 0.73-0.99; I^2 = 93.9%).[24] Some absolute estimates of the impact of vaccination with mRNA vaccines have been proposed to assess the number of maternal and neonatal adverse outcomes that could be avoided such as "Number Needed to Vaccinate (NNV)". The impact measures suggest a benefit in avoiding certain events such as COVID-19 infection (NNV:11), cesarean section delivery (NNV: 182) and prematurity (NNV: 200), while the NNV would be higher for "neonatal problems" (NNV: 463), severe cases of COVID-19 (NNV: 412-2,058), requirement for mechanical ventilation (NNV: 1,371-6,857) and small for gestational age (NNV: >2,000). In all cases far from the "number needed to harm" (NNH) estimated for some adverse events: myocarditis (NNH: 37,000) or thrombosis syndrome with thrombocytopenia (NNH: 50,000).[25] estimates carried out based on observational data in Israel to determine the "Number Needed to Boost" and assess the impact of the third booster dose of the Pfizer-BioNTech vaccine in preventing hospital admissions for COVID-19 and complicated COVID-19 would also suggest a greater potential benefit for pregnant people of advanced age or with comorbidities receiving booster doses.[26]

However, majority of evidence suggesting the possible beneficial effect of vaccination comes from descriptive observational studies. A large population-based descriptive study included more than 180,000 pregnant people in Scotland. The results suggested a possible benefit of vaccination. Thus, 77.4% of all COVID-19 infections, 90.9% of hospital admissions, 98% of ICU hospitalizations and 100% of neonatal deaths occurred in pregnant people diagnosed with COVID who had not been vaccinated.[27] In addition, descriptive data from six European countries from the International Network of Obstetric Survey Systems (INNOSS) have also reported that the highest proportion of ICU admissions of pregnant people corresponded to those who had not been vaccinated (80-100% of the total).[28] On the other hand, in a study in the USA from electronic medical record data in women who had miscarriages, no differences were found in the probability of having been vaccinated (mRNA vaccines) 28 days before the occurrence of the miscarriage.[29]

At present, the benefits of maternal immunization are considered to outweigh the risks and for that reason vaccination is recommended at any time during pregnancy.[1,30,31] The

For peer review only - http://bmjopen.bmj.com/site/about/guidelines.xhtml

recommendation is supported, on the one hand, by the robust evidence indicating a higher morbidity and mortality associated with COVID-19 among pregnant people compared to non-pregnant people; and on the other hand, by the protective effect of maternal immunization observed against complications of COVID-19. Moreover, no pharmacovigilance signals associated with maternal immunization have been detected [13,14,32,33]Finally, some studies suggest a decreased risk of occurrence for certain adverse fetal and neonatal outcomes associated with maternal immunization, although these are considerations based mostly on observational evidence, and in many cases with the caveat that further studies are needed. However, the great difference in the amount of evidence available according to the type of vaccine is striking. Most studies in pregnant people have been carried out with vaccines developed from the newest mRNA platform (Pfizer-BioNTech, Moderna), with very little evidence available for the other vaccines. Perhaps for this reason, some countries prioritize the use of mRNA vaccines in their recommendations, leaving the administration of other (non-mRNA) vaccines with a weaker recommendation based on clinical criteria, weighing the possible risk of infection of the mother considering certain circumstances (e.g.: health workers, comorbidities, etc.) with the theoretical risks that the uncertainties of immunization may entail.[34,35] In spite of the available evidence, most countries that have reported vaccination coverage in pregnant people had low vaccinations rates.[36] For example, in Scotland only 32.3% of pregnant people had received the full scheme (versus 77.4% of non-pregnant people of childbearing age).[27] Other countries have also reported low coverage rates among pregnant people such as Italy (20%) or England (22%) or 30-50% in the Netherlands.[28]

The Latin American Center of Perinatology (CLAP) coordinates a NETWORK of sentinel centers in Latin American and Caribbean countries for the surveillance of maternal health related issues. All centers use SIP-PLUS as a common data collection system. SIP-PLUS database contains the perinatal electronic health record data (Supplemental material I). The perinatal medical record prospectively and systematically records individual data on the clinical follow-up of pregnant people seen from the date of the first antenatal visit to the date of discharge following delivery. It is a healthcare database of perinatal electronic medical records that centralizes the demographic, diagnostic,

For peer review only - http://bmjopen.bmj.com/site/about/guidelines.xhtml

obstetric and neonatal clinical data of mothers and their offspring. In addition, it incorporates the systematic collection of information on the use of vaccines (tetanus/diphtheria, Tdap, influenza, rubella, hepatitis B, hepatitis A), as well as information on dosage, date of vaccination and time of vaccination (before or during pregnancy, or after delivery).[37] In response to the outbreak of the COVID-19 pandemic, CLAP developed a specific SIP form to monitor COVID vaccination of pregnant people, along with other related variables, such as COVID-19 history, which have become part of the routine follow-up of pregnant people (Supplemental material II).

Therefore, this project aims to retrospectively describe the use of COVID-19 vaccines among pregnant people and explore the risk associated with vaccination and selected adverse events, adapting the SIP-PLUS database for surveillance of the use and safety of COVID-19 vaccines from data routinely collected during obstetric and neonatal monitoring of pregnant people in a network of Latin American centers.

GENERAL AND SPECIFIC OBJECTIVES.

General objective.

To describe the utilization of COVID-19 vaccines by pregnant people in hospitals using the SIP-PLUS database and to analyze the risk of vaccination associated with selected maternal and neonatal events.

Specific objectives.

1. To describe the proportion of vaccinated individuals among pregnant people by type of COVID-19 vaccine, number of doses administered, and country.

2. Describe the frequency (Cumulative Incidence) of the following neonatal and maternal events in the cohort of pregnant people: preterm delivery, small for gestational age, low birth.

3. To analyze the associated risk of vaccination for the following neonatal events of interest through an exploration of case-controls nested in a cohort for the following events: prematurity, small for gestational age, and low birth weight.

For peer review only - http://bmjopen.bmj.com/site/about/guidelines.xhtml

Methods and Analysis

1. Design and rationale

Retrospective observational descriptive cohort and analytical study with a nested case-control approach. All outcome and exposure data will be available before the study start date (retrospective analysis).

The dedicated module for capturing COVID-19 vaccine exposure information (including possible heterologous combinations) will be used to obtain the data required. This module complements the current data routinely collected for the vaccines recommended during pregnancy and is collected as part of the obstetric clinical monitoring (Supplemental material II).

2. Source of information and study period.

This study includes secondary data. All the necessary information, including socio-demographic data, comorbidities, exposure to vaccines and events will be retrieved from the SIP-PLUS perinatal electronic medical record database that electronically consolidates perinatal medical record data obtained during routine clinical practice over a retrospective 12-month period.

3. Population, outcome variables, exposure and others.

3.1 Retrospective description of vaccination and event frequency in the pregnant people cohort.

3.1.1 Inclusion criteria

All pregnant people admitted for delivery (singleton) or miscarriage from October 2022 to July 2023.

3.1.2 Exclusion criteria

Twin pregnancies will be excluded.

3.1.3 Adverse events of interest

1. Premature births (preterm delivery)
   Born alive at less than 37 weeks gestational age as registered in the SIP form Clinical record.

For peer review only - http://bmjopen.bmj.com/site/about/guidelines.xhtml

2. Small for gestational age. Live birth classified as small by country standards.
3. Low birth weight. Born alive weighing less than 2500 grams.
4. Stillbirth (at any time).
5. Non-extended neonatal death (max 48h postpartum).
6. Congenital malformations (major and minor).
7. Maternal death (at any time).
8. Maternal Near Miss (MNM). Pregnant people with a MNM condition according to WHO MNM approach.[38]
9. Abortion (spontaneous abortion and therapeutic abortion)

3.1.4 Covariates

Sociodemographic variables:

- Mother's age (years)
- Ethnicity (white, indigenous, mixed, other)
- Marital status (married, common-law marriage, single, other)
- Educational level (none, elementary, high school, college, university)
- Country
- Hospital

Obstetric variables and risk factors (definitions)

- Date of last menstrual period
- Gestational age
- Smoking (yes/no)
- Drug use (yes/no)
- Alcoholism (yes/no)
- Induced labor (yes/no)
- Diabetes
    - Pre-existing (yes/no)
    - Gestational (yes/no)
- High blood pressure
    - Pre-existing (yes/no)
    - Gestational (yes/no)

For peer review only - http://bmjopen.bmj.com/site/about/guidelines.xhtml

- Eclampsia (yes/no)
- Preeclampsia (yes/no)
- Parity (nulliparous, multiparous)
- Inter-gestational period (months)
- COVID infection (describe diagnosis, prior to or during pregnancy)

Administration of other recommended vaccines during pregnancy

- Tetanus/diphtheria (yes/no)
- Tdap (yes/no)
- Hepatitis B (yes/no)
- Influenza (yes/no)
- Any of the other recommended vaccines (yes/no)

3.1.5 Exposure of interest (immunization with COVID-19 vaccines)

Type of vaccine, number of doses administered, and date.

The vaccines of interest will be[33]

-Astra-Zeneca (AZD1222; ChAdOx1 nCoV-19; ChAdOx1-S; Vaxzevria, Covishield)

-Janssen (NJ-78436735; Ad26.COV2-S)

-Moderna (mRNA-1273; Elasomeran)

-Pfizer-BioNTech (BNT162b2; Tozinameran; Comirnaty)

-Sinopharm (Sinopharm/BIB)

-Sinovac (CoronaVac; VeroCell)

-Sputnik- Gamaleya (Gamaleya; Gam-COVIDVac; Adeno-based (rAd26-S+rAd5-S)

The rest of vaccines not considered in the list will be recorded in free text as "other vaccines". When the administration of two different vaccines is included, the vaccination will be classified as "heterologous schedule". On the other hand, it will be considered a complete schedule if the pregnant person received 2 doses (or 1 dose in case of Janssen's vaccine) and a booster schedule if she has received 3 doses (or 2 doses in case of Janssen's vaccine).

For peer review only - http://bmjopen.bmj.com/site/about/guidelines.xhtml

3.2. Analysis of the risk associated with vaccination for the occurrence of the neonatal events of interest through an exploration of case-controls nested in a cohort.

A matched case-control approach nested in a cohort will be carried out for each of the events of interest.

3.2.1 Cohort definition

- Pregnant people (pre-term to late term) and with mother's age data

- Live births with delivery date available based on the best available obstetric estimator.

- Perinatal medical record with "COVID-19 vaccine module" implemented.

3.2.2 Case definition (Events of interest)

1. Premature. Preterm delivery: live birth at less than 37 weeks gestational age according to the best available obstetric estimator
2. Small for gestational age: live birth classified as small based on country standards.
3. Low birth weight: born alive weighing less than 2500 grams.

3.2.3 Definition of controls

Three (uneventful) controls will be randomly selected at the same delivery date of each case (index date) matched by hospital and mother's age (+/- 3 years).

3.2.4 Definition of exposure. The vaccines considered will be those mentioned in section 3.1.5.

Exposure shall be classified according to the following exposure windows**:**

1. **"Late immunization"**: pregnant people immunized on the index date or up to one trimester before the end of pregnancy (third trimester)**.**
2. **Second trimester immunization":** pregnant people immunized after completing the first trimester until the second trimester before the event.
3. **"First trimester immunization"** pregnant people immunized during the first trimester.
4. **"Past immunization"** immunized prior to pregnancy
5. **"Immunization at any time".** Immunization at any of the above times**.**

For peer review only - http://bmjopen.bmj.com/site/about/guidelines.xhtml

Moreover, gestational age (weeks) will be considered if available (Supplementary material 2)

The figure 1 summarizes the overall conceptual framework and procedures of this study.

4. Expected sample size and rationale for its determination.

4.1 General considerations

In estimating the sample size, the frequencies of events observed in a previous analysis in the SIP-PLUS database were considered to estimate the number of potential events (Supplemental material III). Given the lack of precise data on vaccination coverage in hospitals in the CLAP network, literature values from studies published in other countries (prevalences ranging from 20 to 50%) were used as a reference.[27,28] The design of the contrast tests as bilateral (or two-tailed) is because the hypothesis contemplates the scenario of a possible protection against adverse events associated with vaccines and also the opposite scenario, with an increased risk. The correlation coefficient (r) between (matched) cases and controls is not known and cannot be estimated from previous studies. Hence, a conservative value of "r=0.2" has been assumed for the calculation of the sample size, following the recommendation of some authors for these cases.[39] In this part we will consider the analysis of events for which, a priori, there would be sufficient statistical power, anticipating that the measures of association (Odds Ratio) may not be to large based on existing published studies.

4.2. Main analysis of vaccination (any vaccine) with respect to "non-use".

For the matched case-control study (matched with 3 controls) with a power of 80%, alpha error=0.05 (bilateral), a case-control correlation coefficient of 0.2 and a vaccine exposure of 20% would require at least 1009 cases (3,027 controls) to detect an increased probability of vaccine-associated event risk of 30% (OR=1.3) and at least 650 cases (1,950 controls) to detect 30% protection (OR=0.7) (Figures 2 and 3).

4.3. Estimation of the expected number of cases in the database

Based on previous estimates in the SIP-PLUS database and the annual number of deliveries in each hospital of the network, enough cases could be expected annually: 5,731 cases of small for gestational age, 10,932 preterm, and 5,995 low birth weight babies.

For peer review only - http://bmjopen.bmj.com/site/about/guidelines.xhtml

5. Data analysis

5.1 Retrospective description (Cumulative Incidence) of the following maternal and neonatal events in the pregnant cohort: preterm, small for gestational age, low birth weight, stillbirth, neonatal death, congenital malformations, maternal near miss (MNM) and maternal death.

The cumulative incidence will be estimated together with the 95% confidence interval using the exact method. When calculating the cumulative incidence, the number of events of interest will be used in the numerator and different denominators according to the type of event.

1. "Live births" denominator: small for gestational age, premature, low birth weight, stillbirth, MNM, and maternal death.

2. "Total pregnancies" denominator: stillbirth, congenital malformations.

5.2 Description of the proportion of pregnant people who have received a dose of COVID-19 vaccine (total and by type of vaccine) and complete vaccination schedule.

The proportion of vaccinated pregnant people out of the total number of pregnant people in the database will be estimated. The proportions (prevalences) of immunization will be estimated by type of vaccine, vaccine platform (ARNm, Adenoviral vector-based), schedule (complete/incomplete) and time of administration (pre-pregnancy, first trimester, second trimester, third trimester). The total prevalence of maternal immunization (combination of all vaccines administered at any time) will also be estimated. The 95% confidence interval of the proportion will be calculated using the exact method.

5.3 Nested case-control analysis

Logistic regression models will be built for each event of interest, considering COVID-19 vaccine exposure as the main independent variable adjusting for possible confounders (covariates) and estimating the Odds Ratio together with its 95% confidence intervals (CI) as a measure of association (OR). The use of a "standard" (non-conditional conditional) logistic regression analysis will consider adjustment for potential

For peer review only - http://bmjopen.bmj.com/site/about/guidelines.xhtml

For peer review only

confounders and in addition to adjustment variables, but in addition matched variables will be included in the adjusted models, as recommended for the minimization of bias in the analysis of matched case-control studies.[40] Two models will be presented: (1) an unadjusted model that includes only the matching variables (mother's age, date of delivery, and hospital) and (2) a fully adjusted model that also includes all possible confounders described above. As a rule, missing variable data will be treated as a separate (unknown) category. If necessary, missing data will be handled by multiple imputation methods. Analyses by individual vaccine type or platform will be explored. Sensitivity analysis will be performed for different exposure time windows considering the different types of exposure considered in the paper, and their classification according to proximity to the event (late vaccination, second trimester, early), completeness of the schedule (complete or incomplete schedule) and country. Sample size estimation were made using PS: Power and Sample Size Calculation (v. 3.1.6).[41] Statistical analyses will be performed using Stata 17 statistical software (Stata Corp., College Station, TX).

6. It is estimated that a retrospective analysis of all the data will be possible around July 2023 and will be performed centrally by the team that maintains the database. In addition to the cohort description, the risk for adverse events of interest for which the planned sample size has been achieved will be explored. Controls will be obtained from a random selection of pregnant people in the SIP-PLUS database. Three controls will be included for each case matched by maternal age, hospital and delivery date. Patient and public involvement

This protocol presents a retrospective register-based, non-experimental study, and will not have measures of intervention that could burden patients in any way assessable not constituting any risk for patients' participation, and it neither implies the realization of diagnostic tests or additional therapeutic decision besides of what the professional considers timely necessary as part of their clinical practice. Besides, the research question formulation was based upon our own priorities for patient benefit and result interpretation. The nested case–control study described by the protocol is based on clinical records from a network of hospital sites and thus will not include a recruitment process for patients. There was no patient or public involvement in the design of the

For peer review only - http://bmjopen.bmj.com/site/about/guidelines.xhtml

current study protocol nor conduct of the study. However results will be distributed to pregnant people groups, as well as peer-reviewed journals and scientific conferences.

Strengths and Limitations

Risk of indication bias. It is possible that women at higher baseline risk for severe COVID-19 may be more likely to have been vaccinated than those not presenting these factors. Adjusted models will consider inclusion of risk factors (e.g., diabetes, hypertension, BMI≥30 kg/m2) as adjustment variables. [4,42]

Availability of controls. The number of controls available for case-matching may be small. In field studies, obtaining controls is very expensive and getting the desired number may turn out unfeasible. However, in database studies controls are more readily available. Nevertheless, in the exceptional case of lack of sufficient controls. A contingency plan will be carried out considering the following scenarios. Scenario 1. Widening the controls' maternal age to +/- 5 years (keeping the same hospital and delivery date); 2) scenario, delivery date to one week (maintaining maternal age +/- 3 and same hospital).

Possible lack of "non-exposure" to COVID-19 vaccines in pregnant people (case of massive recommendation for maternal immunization and full coverage). We understand that current recommendations are aimed at promoting maternal immunization with COVID-19 vaccines, but full coverage is not anticipated at present. However, an interim analysis is planned at the end of the year to assess the prevalence of exposure by that time, and to readjust the initial estimates of sample size and study potential before the start of the study.

Missing data. As in any study with secondary data, there are always limitations due to incomplete data records, inaccuracies (dates), unmeasured confounding factors, and missing data, etc. In case of missing data for a variable, these will be treated as a separate category in the analysis, and multiple imputation techniques will be considered whenever necessary.

Moreover, the description of abortions will be considered exploratory/hypothetical since: 1) it is not possible to differentiate whether the abortions recorded are spontaneous or induced and, 2) a significant proportion of spontaneous abortions

For peer review only - http://bmjopen.bmj.com/site/about/guidelines.xhtml

occurs on the first weeks of pregnancy and they may not require hospital care nor attended in maternal hospital settings.

Finally, Randomized Clinical Trials (RCTs) are the gold-standard for causal inference. However comparative observational studies may be appropriate when RCTs are not feasible. With the new recommendations for maternal immunization issued by the CDC and WHO RCTs comparing COVID-19 vaccines vs placebo would be difficult to carry out since clinical equipoise will not be reached and administering placebo would be considered unethical. In this scenery retrospective observational studies can provide comparative evidence on COVID-19 vaccine effects versus unexposed pregnant people. Furthermore, information on COVID-19 vaccine safety in pregnant people might mainly be coming, as already mentioned, from high-income countries (HICs). This study represents an opportunity to generate complementary evidence of maternal, foetal and neonatal adverse events from hospitals in middle- and low-income settings but also to gather information for vaccines whose safety profile in pregnancy is less known such as vaccines non authorized nor utilized in HICs.

Ethics and dissemination

The present proposal will be implemented in compliance with the principles of the Declaration of Helsinki.[43] and the international standards for the conduct of epidemiological studies, contained in the International Guidelines for Ethical Review of Epidemiological Studies.[44] The reporting of results will conform to the guidelines for reporting routinely collected and observational pharmacoepidemiology health data (RECORD-PE).[45]The study protocol was reviewed by the Ethical Review Committee on Research of the Panamerican Health Organization. Patient informed consent was waived due to the retrospective design of the study and the utilization of anonymized data (Ref. No: PAHOERC.0546.01). Study results will be disseminated in Open Access journals.

This is an open access article distributed in accordance with the Creative Commons Attribution 4.0 International (CC BY 4.0) license, which permits others to copy and redistribute, remix, adapt, build upon this work for any purpose, even commercially, giving appropriate credit.

For peer review only

Benefit-risk assessment for participants

This retrospective study based on secondary data does not entail any additional risk for the patients because of their participation. Nor does it involve additional diagnostic tests, evaluation or therapeutic decisions beyond those deemed appropriate as part of routine clinical practice. However, the results of the proposal are expected to inform the understanding of the benefit-risk balance of maternal immunization against COVID-19 in the future.

Confidential treatment of data.

As the study will be carried out on an anonymized database without personal identifiers, an informed consent waiver was granted.

Authors' contributions

Diego Macías Saint-Gerons, Alba Maria Ropero, Bremen de Mucio, Claudio Sosa, José Luis Castro, Suzanne Serruya , and Mercedes Colomar wrote the proposal. Desiré Pastor, Monica Chiu, Martha Velandia, Robin Rojas, Edgardo Abalos, Pablo Durán, and Rodolfo Gómez Ponce de León reviewed the proposal and made remarkable contributions. Giselle Tomasso, Luis Mainero and Marcelo Rubino designed the clinical records. All the authors reviewed and agree with the protocol.

Funding statement

This study is funded by Health Canada: Grant Number: 452029: "Access to COVID-19 Vaccines and Therapeutics for Populations in Situations of Vulnerability in the Americas and to support maternal immunization safety related activities"; U.S. Food and Drug Administration grant to support maternal immunization safety related activities, and by the US government (USG) funds through the American Rescue Plan Act.

For peer review only

Competing interest statement.

The authors declare that they have no known competing financial interests or personal relationships that could have appeared to influence the work reported in this paper. The opinions expressed in this article are those of the authors and do not necessarily reflect the views of the Pan American Health Organization (PAHO), its Board of Directors, or the countries they represent.

Figure Legends

Figure 1. Overview of the study procedure for data collection and outcomes of interest

Figure 2. Power of the analysis as a function of the number of cases for different probabilities of exposure to vaccines from 20% to 50% for the detection of an OR=1.3 (increased risk).

Figure 3. Power as a function of the number of cases for different probabilities of exposure to vaccines from 20% to 50% for the detection of an OR=0.7 (protection).

For peer review only

References

1. WHO. WHO SAGE Roadmap For Prioritizing Uses Of COVID-19 Vaccines In The Context Of Limited Supply - An approach to inform planning and subsequent recommendations based on epidemiological setting and vaccine supply scenarios (Version 16 July 2021) [Internet]. 19 July. 2021. Available from: https://reliefweb.int/report/world/who-sage-roadmap-prioritizing-uses-covid-19-vaccines-context-limited-supply-approach
2. Wei SQ, Bilodeau-Bertrand M, Liu S, Auger N. The impact of COVID-19 on pregnancy outcomes: a systematic review and meta-analysis. C Can Med Assoc J = J l'Association medicale Can. 2021 Apr;193(16):E540–8.
3. Adhikari EH, Spong CY. COVID-19 Vaccination in Pregnant and Lactating Women. JAMA [Internet]. 2021 Mar 16 [cited 2022 Jun 6];325(11):1039–40. Available from: https://pubmed.ncbi.nlm.nih.gov/33555297/
4. Allotey J, Stallings E, Bonet M, Yap M, Chatterjee S, Kew T, et al. Clinical manifestations, risk factors, and maternal and perinatal outcomes of coronavirus disease 2019 in pregnancy: living systematic review and meta-analysis. BMJ. 2020 Sep;370.
5. Di Mascio D, Sen C, Saccone G, Galindo A, Grünebaum A, Yoshimatsu J, et al. Risk factors associated with adverse fetal outcomes in pregnancies affected by Coronavirus disease 2019 (COVID-19): a secondary analysis of the WAPM study on COVID-19. J Perinat Med [Internet]. 2020 Jan 1 [cited 2022 Sep 21];49(1):111–5. Available from: https://pubmed.ncbi.nlm.nih.gov/33470966/
6. Ciapponi A, Bardach A, Comandé D, Berrueta M, Argento FJ, Cairoli FR, et al. COVID-19 and pregnancy: An umbrella review of clinical presentation, vertical transmission, and maternal and perinatal outcomes. PLoS One [Internet]. 2021 Jun 1 [cited 2022 Jun 6];16(6). Available from: https://pubmed.ncbi.nlm.nih.gov/34185807/
7. Akhtar H, Patel C, Abuelgasim E, Harky A. COVID-19 (SARS-CoV-2) Infection in Pregnancy: A Systematic Review. Gynecol Obstet Invest [Internet]. 2020 Oct 1 [cited 2022 Jun 6];85(4):295–306. Available from:

For peer review only

https://pubmed.ncbi.nlm.nih.gov/32728006/

8. Beigi RH, Krubiner C, Jamieson DJ, Lyerly AD, Hughes B, Riley L, et al. The need for inclusion of pregnant women in COVID-19 vaccine trials. Vaccine [Internet]. 2021 Feb 5 [cited 2022 Jun 6];39(6):868–70. Available from: https://pubmed.ncbi.nlm.nih.gov/33446385/

9. Macias Saint-Gerons D, Solà Arnau I, De Mucio B, Arévalo-Rodríguez I, Alemán A, Castro JL, et al. Adverse events associated with the use of recommended vaccines during pregnancy: An overview of systematic reviews. Vaccine. 2021 Jul;39 Suppl 2:B12–26.

10. Collier ARY, McMahan K, Yu J, Tostanoski LH, Aguayo R, Ansel J, et al. Immunogenicity of COVID-19 mRNA Vaccines in Pregnant and Lactating Women. JAMA [Internet]. 2021 Jun 15 [cited 2022 Jun 6];325(23):2370–80. Available from: https://pubmed.ncbi.nlm.nih.gov/33983379/

11. Beharier O, Mayo RP, Raz T, Sacks KN, Schreiber L, Suissa-Cohen Y, et al. Efficient maternal to neonatal transfer of antibodies against SARS-CoV-2 and BNT162b2 mRNA COVID-19 vaccine. J Clin Invest [Internet]. 2021 Oct 1 [cited 2022 Jun 6];131(19). Available from: https://pubmed.ncbi.nlm.nih.gov/34596052/

12. Bowman CJ, Bouressam M, Campion SN, Cappon GD, Catlin NR, Cutler MW, et al. Lack of effects on female fertility and prenatal and postnatal offspring development in rats with BNT162b2, a mRNA-based COVID-19 vaccine. Reprod Toxicol [Internet]. 2021 Aug 1 [cited 2022 Jun 6];103:28–35. Available from: https://pubmed.ncbi.nlm.nih.gov/34058573/

13. Shimabukuro TT, Kim SY, Myers TR, Moro PL, Oduyebo T, Panagiotakopoulos L, et al. Preliminary Findings of mRNA Covid-19 Vaccine Safety in Pregnant Persons. N Engl J Med [Internet]. 2021 Jun 17 [cited 2022 Jun 1];384(24):2273–82. Available from: https://www.nejm.org/doi/full/10.1056/NEJMoa2104983

14. Covas DT, de Jesus Lopes de Abreu A, Zampirolli Dias C, Vansan Ferreira R, Gonçalves Pereira R, Silva Julian G. Adverse events of COVID-19 vaccines in pregnant and postpartum women in Brazil: A cross-sectional study. PLoS One. 2023;18(1):e0280284.

15. DeSilva M, Haapala J, Vazquez-Benitez G, Vesco KK, Daley MF, Getahun D, et al. Evaluation of Acute Adverse Events after Covid-19 Vaccination during

For peer review only

Pregnancy. Vol. 387, The New England journal of medicine. United States; 2022. p. 187–9.

16. Goldshtein I, Steinberg DM, Kuint J, Chodick G, Segal Y, Shapiro Ben David S, et al. Association of BNT162b2 COVID-19 Vaccination During Pregnancy With Neonatal and Early Infant Outcomes. JAMA Pediatr [Internet]. 2022 [cited 2022 May 4];176(5). Available from: https://pubmed.ncbi.nlm.nih.gov/35142809/

17. Kachikis A, Englund JA, Singleton M, Covelli I, Drake AL, Eckert LO. Short-term Reactions Among Pregnant and Lactating Individuals in the First Wave of the COVID-19 Vaccine Rollout. JAMA Netw open [Internet]. 2021 [cited 2022 Jun 1];4(8). Available from: https://pubmed.ncbi.nlm.nih.gov/34402893/

18. Magnus MC, Örtqvist AK, Dahlqwist E, Ljung R, Skår F, Oakley L, et al. Association of SARS-CoV-2 Vaccination During Pregnancy With Pregnancy Outcomes. JAMA [Internet]. 2022 Apr 19 [cited 2022 Jun 6];327(15):1469. Available from: https://pubmed.ncbi.nlm.nih.gov/35323851/

19. Zauche LH, Wallace B, Smoots AN, Olson CK, Oduyebo T, Kim SY, et al. Receipt of mRNA Covid-19 Vaccines and Risk of Spontaneous Abortion. N Engl J Med [Internet]. 2021 Oct 14 [cited 2022 Sep 22];385(16):1533–5. Available from: https://pubmed.ncbi.nlm.nih.gov/34496196/

20. Falsaperla R, Leone G, Familiari M, Ruggieri M. COVID-19 vaccination in pregnant and lactating women: a systematic review. Expert Rev Vaccines [Internet]. 2021 [cited 2022 Sep 22];20(12):1619–28. Available from: https://pubmed.ncbi.nlm.nih.gov/34592123/

21. Lipkind HS, Vazquez-Benitez G, DeSilva M, Vesco KK, Ackerman-Banks C, Zhu J, et al. Receipt of COVID-19 Vaccine During Pregnancy and Preterm or Small-for-Gestational-Age at Birth - Eight Integrated Health Care Organizations, United States, December 15, 2020-July 22, 2021. MMWR Morb Mortal Wkly Rep. 2022 Jan;71(1):26–30.

22. Magnus MC, Gjessing HK, Eide HN, Wilcox AJ, Fell DB, Håberg SE. Covid-19 Vaccination during Pregnancy and First-Trimester Miscarriage. N Engl J Med [Internet]. 2021 Nov 18 [cited 2022 Sep 22];385(21):2008–10. Available from: https://pubmed.ncbi.nlm.nih.gov/34670062/

23. Ibroci E, Liu X, Lieb W, Jessel R, Gigase FAJ, Chung K, et al. Impact of prenatal

For peer review only

COVID-19 vaccination on delivery and neonatal outcomes: Results from a New York City cohort. Vaccine. 2023 Jan;41(3):649–56.

24. Prasad S, Kalafat E, Blakeway H, Townsend R, O'Brien P, Morris E, et al. Systematic review and meta-analysis of the effectiveness and perinatal outcomes of COVID-19 vaccination in pregnancy. Nat Commun [Internet]. 2022 Dec 1 [cited 2022 Jul 5];13(1). Available from: https://pubmed.ncbi.nlm.nih.gov/35538060/

25. Magee LA, von Dadelszen P, Kalafat E, Duncan EL, O'Brien P, Morris E, et al. COVID-19 vaccination in pregnancy-number needed to vaccinate to avoid harm. Lancet Infect Dis [Internet]. 2021 Dec 1 [cited 2022 Jun 6];21(12):1627. Available from: https://pubmed.ncbi.nlm.nih.gov/34739865/

26. Kalafat E, Magee LA, von Dadelszen P, Heath P, Khalil A. COVID-19 booster doses in pregnancy and global vaccine equity. Lancet (London, England) [Internet]. 2022 Mar 5 [cited 2022 Jun 6];399(10328):907–8. Available from: https://pubmed.ncbi.nlm.nih.gov/35189078/

27. Stock SJ, Carruthers J, Calvert C, Denny C, Donaghy J, Goulding A, et al. SARS-CoV-2 infection and COVID-19 vaccination rates in pregnant women in Scotland. Nat Med [Internet]. 2022 Mar 1 [cited 2022 Jun 6];28(3):504–12. Available from: https://pubmed.ncbi.nlm.nih.gov/35027756/

28. Engjom H, van den Akker T, Aabakke A, Ayras O, Bloemenkamp K, Donati S, et al. Severe COVID-19 in pregnancy is almost exclusively limited to unvaccinated women - time for policies to change. Lancet Reg Heal Eur [Internet]. 2022 Feb 1 [cited 2022 Sep 22];13. Available from: https://pubmed.ncbi.nlm.nih.gov/35098198/

29. Kharbanda EO, Haapala J, Desilva M, Vazquez-Benitez G, Vesco KK, Naleway AL, et al. Spontaneous Abortion Following COVID-19 Vaccination During Pregnancy. JAMA [Internet]. 2021 Oct 26 [cited 2022 Jun 6];326(16):1629–31. Available from: https://pubmed.ncbi.nlm.nih.gov/34495304/

30. Centers for disease control and prevention (CDC). COVID-19 Vaccines While Pregnant or Breastfeeding [Internet]. 2022 [cited 2022 Nov 23]. Available from: https://www.cdc.gov/coronavirus/2019-ncov/vaccines/recommendations/pregnancy.html#:~:text=COVID-19

For peer review only

vaccination is recommended,become pregnant in the future.

31. WHO. Questions and Answers: COVID-19 vaccines and pregnancy [Internet]. WHO/2019-nCoV/FAQ/Pregnancy/Vaccines/2022.1. 2022 [cited 2022 Sep 22]. p. 1–5. Available from: https://www.who.int/publications/i/item/WHO-2019-nCoV-FAQ-Pregnancy-Vaccines-2022.1
32. DeSilva MB, Haapala J, Vazquez-Benitez G, Boyce TG, Fuller CC, Daley MF, et al. Medically Attended Acute Adverse Events in Pregnant People After Coronavirus Disease 2019 (COVID-19) Booster Vaccination. Obstet Gynecol. 2023 Jul;142(1):125–9.
33. PAHO. Pharmacovigilance for COVID-19 vaccines [Internet]. 2023 [cited 2023 Jul 7]. Available from: https://covid-19pharmacovigilance.paho.org/
34. Kalafat E, O'Brien P, Heath PT, Le Doare K, von Dadelszen P, Magee L, et al. Benefits and potential harms of COVID-19 vaccination during pregnancy: evidence summary for patient counseling. Ultrasound Obstet Gynecol [Internet]. 2021 May 1 [cited 2022 Jun 6];57(5):681–6. Available from: https://pubmed.ncbi.nlm.nih.gov/33734524/
35. Stafford IA, Parchem JG, Sibai BM. The coronavirus disease 2019 vaccine in pregnancy: risks, benefits, and recommendations. Am J Obstet Gynecol [Internet]. 2021 May 1 [cited 2022 Jun 6];224(5):484–95. Available from: https://pubmed.ncbi.nlm.nih.gov/33529575/
36. Blakeway H, Prasad S, Kalafat E, Heath PT, Ladhani SN, Le Doare K, et al. COVID-19 vaccination during pregnancy: coverage and safety. Am J Obstet Gynecol [Internet]. 2022 Feb 1 [cited 2022 Jun 6];226(2):236.e1-236.e14. Available from: https://pubmed.ncbi.nlm.nih.gov/34389291/
37. Gomez Ponce de Leon R, Baccaro LF, Rubio Schweizer G, Bahamondes V, Messina A, de Francisco LA, et al. Building a network of sentinel centres for the care of women in an abortion situation: advances in Latin America and the Caribbean. BMJ Glob Heal. 2022 Oct;7(10).
38. Organization WH. Evaluating the quality of care for severe pregnancy complications: the WHO near-miss approach for maternal health. Geneva: WHO Press; 2011.
39. Dupont WD. Power Calculations for Matched Case-Control Studies. Biometrics.

For peer review only

1988 Dec;44(4):1157.

40. Pearce N. Analysis of matched case-control studies. BMJ [Internet]. 2016 Feb 25 [cited 2022 Sep 22];352. Available from: https://www.bmj.com/content/352/bmj.i969

41. Dupont WD, Plummer WDJ. Power and sample size calculations. A review and computer program. Control Clin Trials. 1990 Apr;11(2):116–28.

42. Zambrano LD, Ellington S, Strid P, Galang RR, Oduyebo T, Tong VT, et al. Update: Characteristics of Symptomatic Women of Reproductive Age with Laboratory-Confirmed SARS-CoV-2 Infection by Pregnancy Status — United States, January 22–October 3, 2020. MMWR Morb Mortal Wkly Rep. 2020 Nov;69(44):1641–7.

43. World Medical Association Declaration of Helsinki: ethical principles for medical research involving human subjects. JAMA [Internet]. 2013 Nov 27 [cited 2022 Jun 7];310(20):2191–4. Available from: https://pubmed.ncbi.nlm.nih.gov/24141714/

44. CIOMS. International Ethical Guidelines for Epidemiological Studies. Geneva; 2009. 130 p.

45. Langan SM, Schmidt SA, Wing K, Ehrenstein V, Nicholls SG, Filion KB, et al. The reporting of studies conducted using observational routinely collected health data statement for pharmacoepidemiology (RECORD-PE). BMJ [Internet]. 2018 [cited 2022 Jun 7];363. Available from: https://pubmed.ncbi.nlm.nih.gov/30429167/

For peer review only

For peer review only - http://bmjopen.bmj.com/site/about/guidelines.xhtml

TABLES

Table 1. Characteristics of the hospitals that use the SIP-PLUS database

Country	Income Level	Name of Hospital	Level	Deliveries attended per year
Argentina	UMIC	Martin Maternity Hospital, Rosario	Tertiary	3600
		Roque Sáenz Peña, Rosario		2500
		Eva Perón, Rosario		1500
Bolivia	LMIC	Bolivian-Japanese Hospital	Tertiary	2719
		La Paz Maternity Hospital	Tertiary	1500
Colombia	UMIC	Rafael Calvo Maternity Clinic	Secondary	7000
		Hospital Universitario del Valle, Cali	Tertiary	7200
Ecuador	UMIC	Isidro Ayora Maternity Hospital	Tertiary	7000
Honduras	LMIC	Roberto Suazo Cordova Hospital	Secondary	3201
		San Felipe Hospital	Secondary	6128
		Leonardo Martínez Valenzuela	Tertiary	18000
Nicaragua	LMIC	España Hospital	Secondary	5250
		Berta Calderón Roque Hospital	Secondary	8993
Dominican Republic	UMIC	San Lorenzo de Los Mina Hospital	Tertiary	13000
Uruguay	UMIC	Pereira Rosell Hospital Center	Tertiary	5000
		Hospital de Clinicas Manual Quintela	Tertiary	800

LMIC: low- and middle-income country; UMIC: upper-middle-income country.

For peer review only

For peer review only

For peer review only - http://bmjopen.bmj.com/site/about/guidelines.xhtml

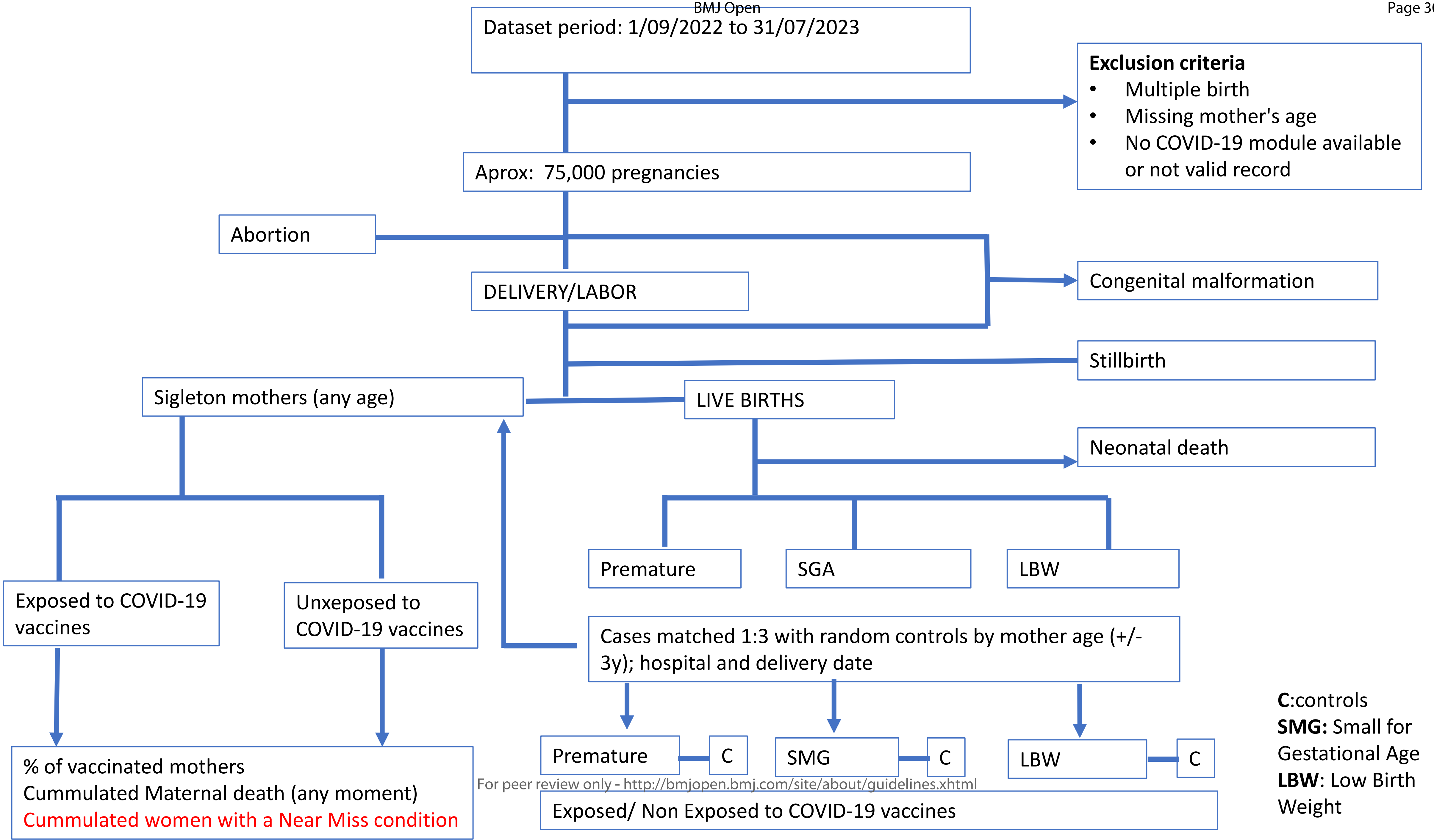
Dataset period: 1/09/2022 to 31/07/2023
Exclusion criteria
• Multiple birth
• Missing mother's age
• No COVID-19 module available or not valid record
Aprox: 75,000 pregnancies
Abortion
DELIVERY/LABOR
Congenital malformation
Stillbirth
Sigleton mothers (any age)
LIVE BIRTHS
Neonatal death
Premature
SGA
LBW
Exposed to COVID-19 vaccines
Unexposed to COVID-19 vaccines
Cases matched 1:3 with random controls by mother age (+/-3y); hospital and delivery date
% of vaccinated mothers
Cummulated Maternal death (any moment)
Cummulated women with a Near Miss condition
Premature
C
SMG
C
LBW
C
Exposed/ Non Exposed to COVID-19 vaccines
C:controls
SMG: Small for Gestational Age
LBW: Low Birth Weight

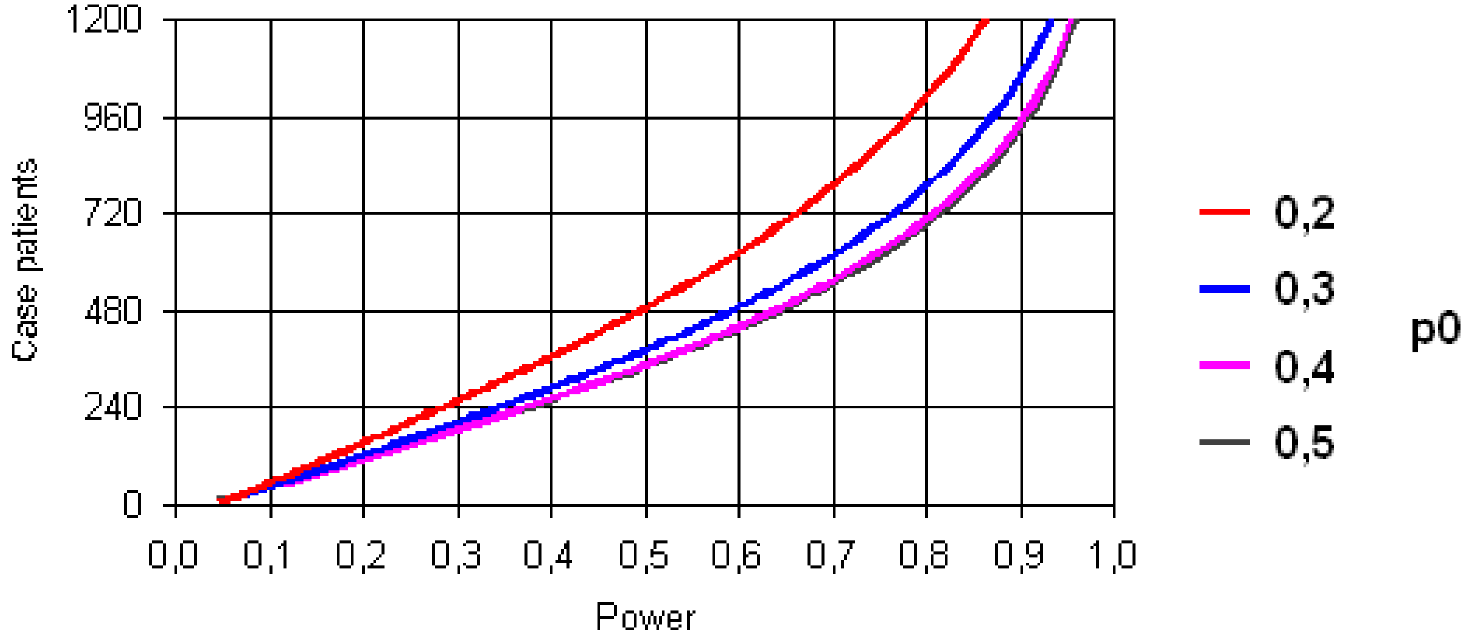
1200
960
720
480
240
0
Case patients
0,0
0,1
0,2
0,3
0,4
0,5
0,6
0,7
0,8
0,9
1,0
Power
0,2
0,3
0,4
0,5
p0

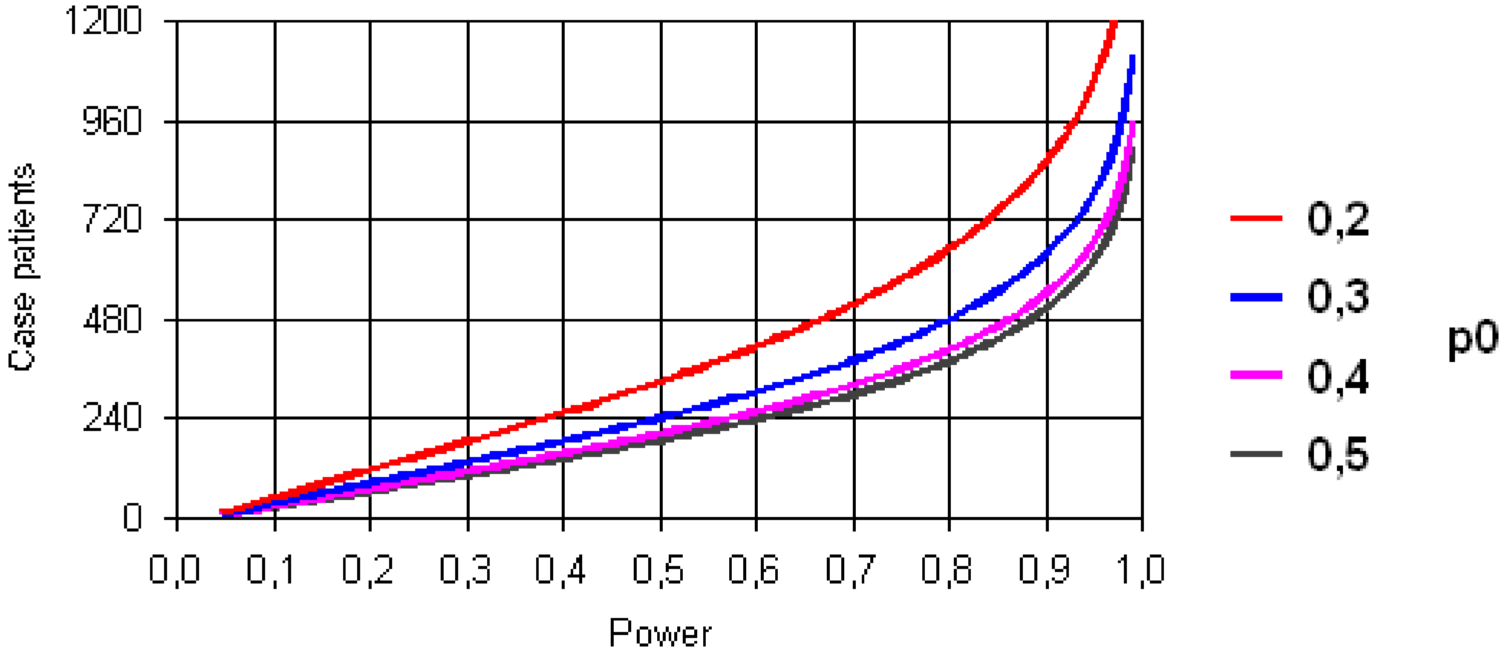
1200
960
720
480
240
0
Case patients
0,0
0,1
0,2
0,3
0,4
0,5
0,6
0,7
0,8
0,9
1,0
Power
0,2
0,3
0,4
0,5
p0

PERINATAL CLINICAL RECORD - CLAP/WR-PAHO/WHO

FIRST NAME | LAST NAME

ADDRESS

DISTRICT | PHONE.

DATE OF BIRTH: day, month, year

AGE (years): < 15, > 35

ETHNICITY: white, indigenous, mixed, black, other

LITERACY: no, yes

EDUCATION: none, primary, secondary, university; years in highest level

CIVIL STATUS: married, common mar., single, other; lives alone: no, yes

Place of antenatal visits | Place of birth | ID #

This color means WATCH (does not necessarily indicate risk or inadequate practices)

HISTORY

FAMILY (no, yes) | PERSONAL (no, yes)

- TBC
- diabetes (I, II, G)
- hypertension
- pre-eclampsia
- eclampsia
- other severe med. cond.

surgery of UT-UG, infertility, heart dis., kidney dis., violence, HIV+ (no, yes)

OBSTETRIC: prev. gest; abortions; vaginal; livebirths; alive

LAST PREVIOUS: n/a, < 2500g, normal, ≥ 4000g

History of twins: no, yes

3 consec. spont. ab.; tubal pregnancy; deliveries; cesar. sections; stillbirths; died 1st week; died after 1st week

END PREVIOUS PREGNANCY: day, month, year; < 1 year

PLANNED PREGNANCY: no, yes

CONTRACEP. METHOD FAILURE: no method, barrier, IUD, horm., emerg., natural

CURRENT PREGNANCY

PREVIOUS WEIGHT (Kg) | HEIGHT (cm)

DLM: day, month, year | EDD: day, month, year

GA BASED on: DLM, Us <20 w.; no, yes

ACT SMO, PAS SMO, DRUGS, ALCOHOL, VIOLENCE (no, yes): 1st trim, 2nd trim, 3rd trim

VACCINES: no; yes before pregnancy; yes during pregnancy; yes after delivery/abortion; day, month, year; number of doses; gest. age weeks

- tetanus diphtheria
- tdap
- influenza
- rubella
- hepatitis B
- hepatitis A

screening hepatitis B no, yes

NORMAL EX. (no, yes): DENTAL, BREAST

CERVIX (normal, abnormal, not done): Visual insp, PAP, COLP

GROUP; Rh: −, +; Inmun: no, yes; γglobulina anti D: no, yes, n/a

TOXOPLASMOSIS (−, +, not done): <20 week IgG; ≥20 week IgG; 1 visit IgM

Hb <20 wk: g, <11.0 g/dl | Presciption Fe/Folates: Fe, Folates; no, yes | Hb ≥20 wk: g, <11.0 g/dl

CHAGAS: −, +, not done

MALARIA: −, +, not done

BACTERIURIA (normal, abnormal, not done): wk <20, ≥20

FASTING BLOOD SUGAR: <20 wk mg/dl; ≥30 wk mg/dl

G.B.S: 35-37 weeks; −, +, not done

PREPARING FOR CHILDBIRTH: no, yes

ADVICE ON BREAST-FEEDING: no, yes

HIV - Diag - Treatment: <20 weeks request (yes, no, n/a), test result (+, −, n/d); ART in preg. (yes, no, n/a); ≥20 weeks request (Si, No, n/a), test result (+, −, n/d); ART in preg. (yes, no, n/a)

SYPHILIS Diagnosis and treatment: Test no treponemic (−, +, n/d), treponemic (−, +, n/d, n/a), <20 wk, weeks; Treatment (no, yes, n/d, n/a), weeks; Treat. partner (no, yes, n/d, n/a); Test no treponemic (−, +, n/d), treponemic (−, +, n/d, n/a), ≥20 wk, weeks; Treatment (no, yes, n/d, n/a), weeks; Treat. partner (no, yes, n/d, n/a)

PRENATAL VISITS

day / month / year	gest age	weight	BP	uterine height	presentation	FHR (bpm)	fetal movements	proteinuria	place of control	alarm signs, test, treatments	Technician's initials	next visit
		,										
		,										
		,										
		,										
		,										

CHILDBIRTH / ABORTION

DATE OF ADMISSION: day, month, year

CARD: no, yes

PRENATAL VISITS total

AGE GEST. 1st CONSULT. weeks

HOSPITAL during PREGNANCY: no, yes; days

ANTENATAL STEROIDS: complete, incompl., no, n/a; week started

ONSET: spontan., induced, elect. ces.

ANTEPARTUM RUPT. OF MEMBRANES: no, yes; day, month, year; hour, min; <37 wk; ≥18 hs.; temp. ≥38ºC

GEST AGE AT CHILDBIRTH: weeks, days; by LMP, by US

PRESENTATION SITUATION: cephalic, breech, transverse

FETAL SIZE ADEQUATE: no, yes

COMPANION (labor, del): couple, relative, other, no one

LABOR

details in partograph: yes, no

hour	min	mother position	BP	pulse	contr./10'	dilatation	height present.	variety posit.	mecon	FHR/dips

DISEASES: Labor Test (−, +, n/d, n/a): Syphilis, HIV; ART: yes, no, n/a

BIRTH

ALIVE; DEAD: antepartum, intrapartum, not known when

hour, min; day, month, year

MULTIPLE: no, yes; order

TERMINATION: spont., forceps, c-sect, vacuum, other

MAIN INDICATION FOR INDUCTION OR SURGERY: code; INDUC.; SURG

POSITION LABOR: sitting, recumb, squat

EPISIOTOMY: no, yes

TEARS: Grade (1 to 4); no

OXYTOCIC: before expul. plac. (no, yes); after (no, yes)

PLACENTA: complete, retained (no, yes)

EARLY CORD CLAMPING: < 1 min., 1 to 3 min.

MEDICATION RECEIVED (no, yes): oxytocic at labor, ATB, analges, local anesth, region anesth, gral anesth, transfusion, Mg sulfate (preeclam., eclam.), others (code): medic 1, medic 2

NEONATE

SEX: f, m, not defined

BIRTH WEIGHT: g; <2500 g, ≥4000 g

HEAD CIRC cm | LENGTH cm

GESTATIONAL AGE: weeks, days; LMP, US, ESTIMATED

WEIGHT G.A.: adeq., small, large

APGAR (min): 1st, 5th

Early intitiation of breast feeding: no, yes

RESUSCITATION (no, yes): stimul., suction, mask, oxygen, massage, tube, medication

ATTENDED (doctor, obst., nuerse, assist., stud., TBA, other, Name): CHILDBIRTH, NEONATE

CONGENITAL DEFECTS: no, minor, major; code

DISEASES: 1 o more, none; code

NEONATAL SCREENING: HIV in NN (Exposed: no, yes, s/d; Treated: no, yes, s/d, n/a); VDRL (−, +, not done); Tx (no, yes, n/a, s/d); Hearing (−, +, not done); Chagas disease; Bili rrub; Toxo IgM; Hb pathy; Cardio-vascular disease; Metabolic; not done; done

Death at site of birth (no, yes): Mother, NN

REFERRAL: room in, neona tol, another hosp.

POSTPARTUM

time	Temperature ºC	B.P.	pulse	uterine involution	Bleeding	Responsible
	,					
	,					
	,					
	,					
	,					
	,					

γglobulin anti D: no, yes, n/a

NEONATAL DISCHARGE

alive, dead; day, month, year; hour, min

transfer; place

dies during or at transfer: no, yes, s/d

AGE AT DISCHARGE complete day: <1 day

FEEDING AT DISCH: excl. breastf, partial, artificial

Supine: no, yes | BCG: no, yes | INMUN. HEPATITIS B: no, yes

Meconium 1st day: no, yes

WEIGHT AT DISCHARGE

Id. NN | Name NN | Responsabile

HCP NEAR MISS ANV_ENG 02-08-201087

For peer review only

M O R B I L I T Y

HYPERTENSIVE DISORDERS (no / yes)

	no	yes
Chronic hypertension	○	●
Mild preeclampsia	○	●
Severe preeclampsia	○	●
Eclampsia	○	●
HELLP	○	●
Gestational Hypertension	○	●
Chronic hypertension + preeclampsia	○	●

INFECTIONS (no / yes)

	no	yes
Sepsis	○	●
Pyelonephritis	○	●
Endometritis	○	●
Pneumonia	○	●
Caesarean section wound infection	○	●
Chorioamnionitis	○	●
Episiorrhaphy infection	○	●
Asymptomatic bacteriuria	○	●
Other infections	○	●

HEMORRHAGE

1st TRIMESTER (no / yes) 2nd TRIMESTER (no / yes) 3rd TRIMESTER (no / yes)

	no	yes
Post abortion	○	●
Hydatidiform Mole	○	●
Ectopic pregnancy	○	●
Previous placenta	○	●
Accretism	○	●
Abruptio placentae	○	●
Uterine rupture	○	●
Postpartum hemorrhage	○	●
Uterine atony	○	●
Lacerations	○	●
Ovular remains	○	●
Coagulation defect	○	●

METABOLIC DISORDERS (no / yes)

MELLITUS DIABETES (no / yes)

	no	yes	not performed
Abnormal oral tolerance to glucose load	○	●	○
Previous M. D. (insulin dependent)	○	●	
Previous M. D. (non insulin dependent)	○	●	
Gestational DM	○	●	
Hyperosmolar state	○	●	
Cetoacidosis	○	●	
Hyperglycemic state	○	●	

THYROID DISORDERS (no / yes)

	no	yes
Hypothyroidism	○	●
Hyperthyroidism	○	●
Thyroid crisis	○	●
Another metabolic disorder	○	●

OTHER DISORDERS (no / yes)

	no	yes
Hyperemesis gravidarum	○	●
Deep vein thrombosis	○	●
Pulmonary embolism	○	●
Amniotic fluid embolism	○	●
Heart disease	○	●
Valvulopathy	○	●
Seizures	○	●
Altered state of consciousness	○	●
Oliguria	○	●
Anemia	○	●
Sickle cell anemia	○	●
Renal disease	○	●
Malignant neoplasia	○	●
Psychiatric disorder	○	●
Cholestasis	○	●
Others	○	●

OBSTETRIC COMPLICATIONS (no / yes)

	no	yes
Obstructed labor	○	●
Prolonged rupture of membranes	○	●
Oligoamnios	○	●
Other	○	●
Polyhydramnios	○	●
Acute fetal distress	○	●
Intrauterine growth restriction	○	●

INTERVENTIONS

	no	yes	s/d
Manual removal	○	●	○
Uterotonic to treatment of hemorrhag	○	●	○
Central venous catheter	○	●	○
Administration of blood products	○	●	○
Laparotomy (excluding caesarean section)	○	●	○
ICU admission <7 days	○	●	○
Using ATB iv to treatment of infectious complication	○	●	○

Which ones?

Nº of vol.

days

Which ones?

	no	yes
No pneumatic shock-suit	○	●
Hydrostatic balls	○	●
B-Lynch suture or similar	○	●
Hemostatic ligature of uterine / hypogastric arteries	○	●
Embolizations	○	●

VARIABLES TO IDENTIFY NEAR MISS CASES (no / yes)

CLINICAL CRITERIA / BODY-SYSTEM DYSFUNCTION

	no	yes
Cardiovascular		
Shock	○	●
Heart attack	○	●
Hepatic		
Jaundice in preeclampsia	○	●
Respiratory		
Acute cyanosis	○	●
Wheezing (gasping)	○	●
Severe tachypnea RF> 40 rpm	○	●
Severe bradypnea RF <6 rpm	○	●
Renal		
Oliguria non responsive to fluids or diuretics	○	●
Hematological/coagulation		
Failure to form clots	○	●
Neurological		
Coma	○	●
Prolonged unconsciousness (lasting >12 hours	○	●
Stroke	○	●
Uncontrollable fits/status epileptics	○	●
Total paralysis	○	●

LAB

Laboratory	no	yes
Platelets/ml < 50.000	○	●
Creatinine ≥ 300 µmol/l or ≥ 3,5 mg/dl	○	●
Bilirubin > 100 µmol/l or > 6,0 mg/dl	○	●
pH < 7,1	○	●
Sat Hb < 90% ≥ 1 hour	○	●
PaO_2/FiO_2 < 200 mmHg	○	●
Lactato > 5 µmol/L o 45 mg/dl	○	●

INTERVENTIONS

	no	yes
Use of continuous vasoactive drug	○	●
Intubation and ventilation not related to anesthesia	○	●
Massive transfusion of blood or red cells (≥ 3 units)	○	●
Admission to UCI ≥ 7 days	○	●
Histerectomy	○	●
Dialysis for acute renal failure	○	●
Cardiopulmonary resucitation	○	●

Which ones?

days

days

Criteria Near Miss: FLASOG ≥ 3 vol. WHO ≥ 5 vol.

PUERPERIUM

day	Temp ºC	blood prasure	pulse	uter. invol.	lochia	perine	breast feeding	observations	Responsabile
1st	,								
2nd	,								
3rd	,								
5th to 10th	,								

MATERNAL DISCHARGE

DATE: day / month / year — hour / min

TYPE OF DISCHARGE: dead ○ — against medical advise ● — discharge ○

CONDITION AT DISCHARGE: healthy ○ — NA ● — with pathology ● — dead ●

Place — Transfer

passes away during or after transfer: no ○ yes ●

Autopsy: no ○ yes ●

Responsabile

CONTRACEPTION

Contraception councelling: oral ○ — written ○ — none ●

Responsabile

Start contraception	no ●	yes ○

	pref.	acced.
IUD	○	○
injectable	○	○
barrier	○	○
tube ligation	○	○
hormonal contraception	○	○
Other hormonal (vaginal ring, patch)	○	○
male surgical sterilization	○	○
hormonal implant	○	○
condom	○	○
abstinence	○	○

HCP NEAR MISS REVERSO (Regional) INGLES 06-08-2018

This color means WATCH (does not necessarily indicate risk or inadequate practices)

For peer review only

BMJ Open

NAME

LAST NAME

Institution

Country code

Type of doc.

ID number

Had COVID-19 in the 12 months before the pregnancy termination?

	yes	no	Date (month / day / year)	Diagnostic test: PCR	Rapid	doesn't know
Before pregnancy			month / day / year			
During pregnancy 1st. contagion			month / day / year			
During pregnancy 2nd contagion			month / day / year			

Indicate the greater severity of the contagion(s) during pregnancy

	Asymptomatic - home	Symptomatic - home	Inpatient – intermediate	Inpatient – intensive
1st. contagion				
2nd contagion				

Did she receive any dose of the COVID-19 vaccine in the last year? yes no **(If yes, indicate from the most recent to the oldest)**

Date (month / day / year)		Gestational age	Did she present certificate/card? yes	no	AstraZeneca (AZD1222, ChAdOx1 nCoV-19, ChAdOx1-S, Vaxzevria, Covishield)	Janssen (JNJ-78436735; Ad26.COV2-S)	Pfizer/BioNtech (BNT162b2; Tozinameran; Comirnaty)	Sputnik V (Gamaleya; Gam-COVID-Vac; Adeno-based (rAd26-S+rAd5-S))	Sinopharm/ BIBP (Sinopharm/BIB)	Sinovac (CoronaVac; VeroCell)	Moderna (mRNA-1273; Elasomeran)	doesn't know	Other
month / day / year	or												Which one?:
month / day / year	or												Which one?:
month / day / year													Which one?:

For peer review only

For peer review only - http://bmjopen.bmj.com/site/about/guidelines.xhtml

Supplemental material III. Births attended and estimated number of potential cases per year.

Country	Hospital Name	Deliveries per year	Number of potential events per year		
			Small for gestational age	Preterm	Low birth weight
Bolivia	Hospital Boliviano Japonés	2719	177	337	185
	Maternidad de la Paz	1500	98	186	102
Colombia	Clínica Maternidad Rafael Calvo	7000	455	868	476
	Hospital Universitario del Valle, Cali	7200	468	893	497
Honduras	Hospital Roberto Suazo Cordova	27300	1775	3385	1856
	Hospital de San Felipe				
	Leonardo Martinez Valenzuela				
	Hospital Berta Calderón Roque				
República Dominicana	Hospital San Lorenzo de Los Mina	13000	845	1612	884
Argentina	Maternidad Martin	4200	273	349	291
	Roque Saenz Peña	2500	163	310	170
	Eva Peron	1500	98	186	102
Ecuador	Isidro Ayora	7000	455	868	476
Uruguay	Centro hosp Pereira Rosell	5000	325	620	340
	Hospital de Clinicas	800	52	99	54
Total SIP PLUS	-	93962	5731	10932	5995

BMJ Open

Description of maternal and neonatal adverse events in pregnant people immunized with COVID-19 vaccines during pregnancy in the CLAP NETWORK of sentinel sites. Nested case-control analysis of the immunization-associated risk: A study protocol

Journal:	*BMJ Open*
Manuscript ID	bmjopen-2023-073095.R2
Article Type:	Protocol
Date Submitted by the Author:	04-Oct-2023
Complete List of Authors:	Macías Saint-Gerons, Diego ; Department of Medicine, University of Valencia, INCLIVA Health Research Institute and CIBERSAM, Department of Medicine; PAHO<br>Castro, José ; Fundación para la innovación, la formación, la investigación y el desarrollo comunitario (FUNDEC); PAHO<br>Colomar, Mercedes; Unidad de Investigación Clínica y Epidemiológica Montevideo; PAHO, Latin American Center of Perinatology, Women and Reproductive Health (CLAP/WR)<br>Rojas, Robin; PAHO<br>Sosa, Claudio; Universidad de la Republica Uruguay, Department of Obstetrics and Gynecology, Pereira Rossell Hospital, School of Medicine; PAHO, Latin American Center of Perinatology, Women and Reproductive Health (CLAP/WR)<br>Ropero, Alba ; PAHO<br>Serruya, Suzanne; PAHO, Latin American Center of Perinatology, Women and Reproductive Health (CLAP/WR)<br>Pastor, Desiré; PAHO<br>Chiu, Monica; PAHO<br>Velandia-Gonzalez, Martha; PAHO<br>Abalos, Edgardo; Centro de Estudios de Estado y Sociedad (CEDES)<br>Durán, Pablo; PAHO, Latin American Center of Perinatology, Women and Reproductive Health (CLAP/WR)<br>Gomez Ponce de León, Rodolfo; PAHO, Latin American Center of Perinatology, Women and Reproductive Health (CLAP/WR)<br>Tomasso, Giselle; Unidad de Investigación Clínica y Epidemiológica Montevideo; PAHO, Latin American Center of Perinatology, Women and Reproductive Health (CLAP/WR)<br>Mainero, Luis; PAHO, Latin American Center of Perinatology, Women and Reproductive Health (CLAP/WR)<br>Rubino, Marcelo; PAHO, Latin American Center of Perinatology, Women and Reproductive Health (CLAP/WR)<br>De Mucio, Bremen; PAHO, Latin American Center of Perinatology, Women and Reproductive Health (CLAP/WR)
**Primary Subject Heading**:	Infectious diseases

Secondary Subject Heading:	Obstetrics and gynaecology, Public health
Keywords:	PERINATOLOGY, COVID-19, Maternal medicine < OBSTETRICS

SCHOLARONE™
Manuscripts

Description of maternal and neonatal adverse events in pregnant people immunized with COVID-19 vaccines during pregnancy in the CLAP NETWORK of sentinel sites. Nested case-control analysis of the immunization-associated risk: A study protocol.

Diego Macías Saint-Gerons (0000-0002-2572-2160), José Luis Castro (0000-0002-9179-113X), Mercedes Colomar (0000-0001-7424-5551), Robin Rojas (0000-0002-4046-9298), Claudio Sosa (0000-0002-2539-0848), Alba Maria Ropero (0000-0001-9960-7491), Suzanne Serruya (0000-0003-1371-4558), Desiré Pastor, Monica Chiu (0000-0002-2730-8625), Martha Velandia (0000-0002-7636-3604), Edgardo Abalos (0000-0001-6653-429X), Pablo Durán (0000-0003-1032-487X), Rodolfo Gómez Ponce de León (0000-0001-9914-7130), Giselle Tomasso (0000-0002-4424-1581), Luis Mainero, Marcelo Rubino, Bremen de Mucio (0000-0003-0662-9742)

Department of Medicine, University of Valencia, INCLIVA Health Research Institute and CIBERSAM, Department of Medicine, Valencia, Comunitat Valenciana Diego Macías Saint-Gerons International PAHO consultant, FÜNDEC: Fundación para la innovación, la formación, la investigación y el desarrollo comunitario, Santa Cruz de Tenerife José Luis Castro International PAHO consultant, Montevideo Clinical and Research Unit. Hospital de Clinicas, 11300 Montevideo, Uruguay Mercedes Colomar International PAHO consultant,

Pan-American Health Organization (PAHO), 525 23rd St NW, Washington DC 20037, United States Robin Rojas Technical Officer, Universidad de la República Uruguay, Department of Obstetrics and Gynecology, Pereira Rossell Hospital, School of Medicine, 11300 Montevideo, Uruguay Claudio Sosa International PAHO consultant, Pan-American Health Organization (PAHO), 525 23rd St NW, Washington DC 20037, United States Alba Maria Ropero Regional Advisor on Immunization, Latin American Center of Perinatology, Women and Reproductive Health (CLAP/WR), Av. Brasil 2697, 11300 Montevideo, Uruguay Suzanne Serruya CLAP Director, Pan-American Health Organization (PAHO), 525 23rd St NW, Washington DC 20037, United States Desiré

For peer review only - http://bmjopen.bmj.com/site/about/guidelines.xhtml

Pastor Regional Immunization Advisor, Pan-American Health Organization (PAHO), 525 23rd St NW, Washington DC 20037, United States Monica Chiu International PAHO consultant, Pan-American Health Organization (PAHO), 525 23rd St NW, Washington DC 20037, United States Martha Velandia Advisor Immunization, Centro de Estudios de Estado y Sociedad (CEDES), Sánchez de Bustamante 27, Ciudad Autónoma de Buenos Aires, Argentina, Edgardo Abalos CLAP Associate researcher, Latin American Center of Perinatology, Women and Reproductive Health (CLAP/WR), Av. Brasil 2697, 11300 Montevideo, Uruguay Pablo Durán advisor, Latin American Center of Perinatology, Women and Reproductive Health (CLAP/WR), Av. Brasil 2697, 11300 Montevideo, Uruguay Rodolfo Gómez Ponce de León advisor, Montevideo Clinical and Research Unit. Hospital de Clinicas, 11300 Montevideo, Uruguay Giselle Tomasso International PAHO consultant, Latin American Center of Perinatology, Women and Reproductive Health (CLAP/WR), Av. Brasil 2697, 11300 Montevideo, Uruguay Luis Mainero IT expert, Latin American Center of Perinatology, Women and Reproductive Health (CLAP/WR), Av. Brasil 2697, 11300 Montevideo, Uruguay Marcelo Rubino data analyst, Latin American Center of Perinatology, Women and Reproductive Health (CLAP/WR), Av. Brasil 2697, 11300 Montevideo, Uruguay Bremen de Mucio Advisor.

Correspondence to: Mercedes Colomar; +59899114204; mcolomar@unicem-web.org; Julio Maria Sosa 2281, CP 11300, Montevideo, Uruguay.

Word count: 4824

Keywords:

Perinatology; Maternal Health; Pharmacovigilance; Infant, Premature Birth; Infant, Small for Gestational Age; Low Birth Weight; Pregnancy Outcome; COVID-19 vaccines.

Abstract

Introduction

Coronavirus disease is associated with higher morbi-mortality in pregnant people compared to non-pregnant people. At present, the benefits of maternal immunization

For peer review only

are considered to outweigh the risks and therefore vaccination is recommended during pregnancy. However, additional information is needed on the safety of the vaccines in this population.

Methods and analysis

This a retrospective cohort nested case–control study in pregnant people who attended maternity hospitals from 8 Latin American and Caribbean countries. A perinatal electronic clinical history database with neonatal and obstetric information will be used. The proportion of pregnant people immunized with COVID-19 vaccines of the following maternal and neonatal events will be described: preterm infant, small for gestational age, low birth weight, stillbirth, neonatal death, congenital malformations, maternal near miss, and maternal death. Moreover, the risk of prematurity, small for gestational age, and low birth weight associated with exposure to COVID-19 vaccines will be estimated. Each case will be matched with two groups of three randomly selected controls. Controls will be matched by hospital and mother's age (+/- 3 years) with an additional matching by delivery date and conception time in the first and second control groups, respectively. The estimated required sample size for the main analysis (exposure to any vaccine) concerning "non-use" is at least 1009 cases (3,027 controls) to detect an increased probability of vaccine-associated event risk of 30% and at least 650 cases (1,950 controls) to detect 30% protection. Sensitivity and secondary analyses considering country, type of vaccine, exposure windows, and completeness of immunization will be reported.

Ethics

The study protocol was reviewed by the Ethical Review Committee on Research of the Panamerican Health Organization. Patient informed consent was waived due to the retrospective design and the utilization of anonymized data (Ref. No: PAHOERC.0546.01. Results will be disseminated in Open Access journals.

Strengths and limitations of this study

- Pregnant people were excluded from the pivotal clinical trials of COVID-19 vaccines and most of the evidence from vaccine safety comes from high income country studies.

For peer review only - http://bmjopen.bmj.com/site/about/guidelines.xhtml

- This study represents an opportunity to generate complementary evidence of maternal, foetal and neonatal adverse events after administration of COVID-19 vaccines currently used in LAC countries for maternal immunization.
- The use of the SIP-PLUS database allows achieving a large sample size necessary to explore potential vaccine-associated discrete effects (beneficial or harmful) in a timely manner.
- As in any study using routinely collected data there are further potential limitations due to incomplete data records, inaccuracies (dates), unmeasured confounding factors, and missing data.

  The number of "non exposed" pregnant people could decrease over time and may introduce affect statistical power and interpretation.

Introduction

Pregnant people do not appear to have a higher susceptibility to SARS-CoV-2 coronavirus infection than nonpregnant people.(1) However, coronavirus disease 2019 (COVID-19) is associated with higher morbidity and mortality among pregnant people compared with non-pregnant people.(2,3) Mortality in pregnant people with COVID-19 could be 70% higher, and the risk of admission to intensive care units (ICU) or the need for mechanical ventilation would be three times higher compared to non-pregnant unvaccinated people of similar age.(3,4) Pregnant people with COVID-19 show an increased risk of occurrence of some adverse maternal-fetal outcomes such as increased risk of preterm delivery, preeclampsia, or miscarriage compared to pregnant people without COVID-19.(2) It has been observed that, in its most complicated or critical forms, COVID-19 infection is associated with prematurity, gestational diabetes, preeclampsia and low birth weight.(3) Neonates born to mothers with COVID-19 appear to have a higher probability of admission to a neonatal care unit (NCU), which could be attributed to a greater extent to the negative effect of COVID-19 on maternal health during gestation than to vertical transmission.(4–6) Besides other maternal, fetal, and neonatal complications associated with COVID-19 have also been reported. A systematic review described adverse events such as fetal distress, premature rupture of membranes, and other neonatal adverse events such as respiratory distress, gastrointestinal events, and fever. Maternal death reports have been more rare.(7)

For peer review only - http://bmjopen.bmj.com/site/about/guidelines.xhtml

After a global research effort to develop effective treatments and vaccines against COVID-19, vaccines became available on January 2021. It should be noted that a characteristic of the new COVID-19 vaccines is that they were developed from a range of different platforms: viral vector (adenoviral), mRNA platform, inactivated, attenuated viruses, being pregnancy an exclusion criterion in the main pivotal clinical trials undertaken for the authorization of these COVID-19 vaccines.(8) Besides, vaccines that are currently recommended for maternal immunization - and with which there is most experience - such as the influenza vaccine or the Tdap vaccine, do not use mRNA-based platforms as is the case with the Pfizer-BioNTech and Moderna vaccines.(9) However, despite being based on a newer platform, mRNA vaccines do not entail the risk of acquiring vaccination-induced infection, as might be the case with the use of attenuated/inactivated vaccines. On the other hand, they could be useful in pregnant people, as a robust transfer of the humoral response to the fetus has been observed with mRNA vaccines.(10,11) Furthermore, no remarkable adverse events have been detected in experimental genotoxicity studies conducted on rats.(12)

Although various clinical trials have been planned for the study of COVID-19 vaccines in pregnant people, most of the initiatives are based on the creation of registries of pregnant people exposed to the vaccines. In many cases, it is the adaptation of previous registries that had been created to study the effects of COVID-19 infection in pregnant people. In this respect, Moderna created a registry to monitor pregnancy outcomes in individuals who received their COVID-19 vaccine. PREGISTRY - for example - is a startup focused on improving maternal-fetal health, in collaboration with the Harvard School of Public Health (https://www.pregistry.com); aim to enroll 8,000 pregnant people in their registry to evaluate their obstetric, neonatal, and infant outcomes following COVID-19 immunization. In the UK, the UKOSS registry (https://www.npeu.ox.ac.uk/ukoss) is collecting data to allow the parallel nesting of cohort and case-control studies. The CDC's "v-safe" registry have reported preliminary results with observed frequencies of spontaneous abortion and fetal death in vaccinated pregnant people similar to the expected frequencies in the general population of pregnant people of the same age and without known comorbidities.(13)

For peer review only - http://bmjopen.bmj.com/site/about/guidelines.xhtml

Apart from these active registries that include pregnant people, there is little evidence from published studies with complete data on the effectiveness and safety of vaccination for COVID-19 in this population and information from surveillance systems, monitoring acute adverse events following immunization during pregnancy, did not find increased risk of clinically serious acute adverse in different populations groups.(14,15) One retrospective cohort study observed that, in pregnant people, vaccination with Pfizer-BioNTech was associated with a lower risk of SARS-CoV-2 infection compared to those who did not receive vaccination (HR:0.22; 95% CI: 0.11-0.43).(16) In another prospective cohort study, short-term adverse events associated with mRNA vaccines were similar among pregnant people to those occurring in nonpregnant people of the same childbearing age.(17) In another large comparative study involving 157,521 singleton pregnancies from the Swedish pregnancy and Norwegian childbirth electronic registries, no statistically significant differences were found in the risk for fetal death, small for gestational age rates, preterm, and low APGAR score value in mRNA and Pfizer-BioNTech vaccinated and unvaccinated subjects.(18) Also, the risk of miscarriage does not appear to be higher than expected among people immunized before or during pregnancy.(19,20) Moreover, in a retrospective cohort including more than 40.000 pregnant people, COVID-19 vaccines (mostly mRNA) were not associated with preterm birth or small-for-gestational-age at birth.(21) On the other hand, a case-control study using Norwegian registry data observed no statistically significant risk difference for first-trimester miscarriage in pregnant people exposed to COVID-19 vaccines (Pfizer-BioNTech, Moderna, Astra) and those not immunized.(22) A study investigating on the associations of COVID-19 vaccination throughout pregnancy with delivery and neonatal outcomes suggests that there is no associated risk between prenatal COVID-19 vaccination and adverse delivery and neonatal outcomes in a cohort sample from NYC.(23)A systematic review and meta-analysis included data from 23 studies and 117,552 pregnant people immunized mostly with mRNA vaccines. In this meta-analysis, which accumulates the most evidence on the subject to date, no differences were found in vaccinated compared to unvaccinated pregnant people with regards the risk of occurrence of miscarriage, earlier gestation at birth, placental abruption, pulmonary embolism, postpartum hemorrhage, maternal death, mothers' admission to the ICU, low birth weight, or newborns' admission to the ICU. The meta-analysis identified that

For peer review only

immunization conferred a 15% protective effect against fetal death, although the heterogeneity found was very high (Odds Ratio: 0.85; 95%CI: 0.73-0.99; I2 = 93.9%).(24) Some absolute estimates of the impact of vaccination with mRNA vaccines have been proposed to assess the number of maternal and neonatal adverse outcomes that could be avoided such as "Number Needed to Vaccinate (NNV)". The impact measures suggest a benefit in avoiding certain events such as COVID-19 infection (NNV:11), cesarean section delivery (NNV: 182) and prematurity (NNV: 200), while the NNV would be higher for "neonatal problems" (NNV: 463), severe cases of COVID-19 (NNV: 412-2,058), requirement for mechanical ventilation (NNV: 1,371-6,857) and small for gestational age (NNV: >2,000). In all cases far from the "number needed to harm" (NNH) estimated for some adverse events: myocarditis (NNH: 37,000) or thrombosis syndrome with thrombocytopenia (NNH: 50,000).(25) estimates carried out based on observational data in Israel to determine the "Number Needed to Boost" and assess the impact of the third booster dose of the Pfizer-BioNTech vaccine in preventing hospital admissions for COVID-19 and complicated COVID-19 would also suggest a greater potential benefit for pregnant people of advanced age or with comorbidities receiving booster doses.(26)

However, majority of evidence suggesting the possible beneficial effect of vaccination comes from descriptive observational studies. A large population-based descriptive study included more than 180,000 pregnant people in Scotland. The results suggested a possible benefit of vaccination. Thus, 77.4% of all COVID-19 infections, 90.9% of hospital admissions, 98% of ICU hospitalizations and 100% of neonatal deaths occurred in pregnant people diagnosed with COVID who had not been vaccinated.(27) In addition, descriptive data from six European countries from the International Network of Obstetric Survey Systems (INNOSS) have also reported that the highest proportion of ICU admissions of pregnant people corresponded to those who had not been vaccinated (80-100% of the total).(28) On the other hand, in a study in the USA from electronic medical record data in women who had miscarriages, no differences were found in the probability of having been vaccinated (mRNA vaccines) 28 days before the occurrence of the miscarriage.(29)

At present, the benefits of maternal immunization are considered to outweigh the risks and for that reason vaccination is recommended at any time during pregnancy.(1,30,31)

For peer review only - http://bmjopen.bmj.com/site/about/guidelines.xhtml

The recommendation is supported, on the one hand, by the robust evidence indicating a higher morbidity and mortality associated with COVID-19 among pregnant people compared to non-pregnant people; and on the other hand, by the protective effect of maternal immunization observed against complications of COVID-19. Moreover, no pharmacovigilance signals associated with maternal immunization have been detected (13,14,32,33)Finally, some studies suggest a decreased risk of occurrence for certain adverse fetal and neonatal outcomes associated with maternal immunization, although these are considerations based mostly on observational evidence, and in many cases with the caveat that further studies are needed. However, the great difference in the amount of evidence available according to the type of vaccine is striking. Most studies in pregnant people have been carried out with vaccines developed from the newest mRNA platform (Pfizer-BioNTech, Moderna), with very little evidence available for the other vaccines. Perhaps for this reason, some countries prioritize the use of mRNA vaccines in their recommendations, leaving the administration of other (non-mRNA) vaccines with a weaker recommendation based on clinical criteria, weighing the possible risk of infection of the mother considering certain circumstances (e.g.: health workers, comorbidities, etc.) with the theoretical risks that the uncertainties of immunization may entail.(34,35) In spite of the available evidence, most countries that have reported vaccination coverage in pregnant people had low vaccinations rates.(36) For example, in Scotland only 32.3% of pregnant people had received the full scheme (versus 77.4% of non-pregnant people of childbearing age).(27) Other countries have also reported low coverage rates among pregnant people such as Italy (20%) or England (22%) or 30-50% in the Netherlands.(28)

The Latin American Center of Perinatology (CLAP) coordinates a NETWORK of sentinel centers in Latin American and Caribbean countries for the surveillance of maternal health related issues (see table 1). All centers use SIP-PLUS as a common data collection system. SIP-PLUS database contains the perinatal electronic health record data (Supplemental material I). The perinatal medical record prospectively and systematically records individual data on the clinical follow-up of pregnant people seen from the date of the first antenatal visit to the date of discharge following delivery. It is a healthcare database of perinatal electronic medical records that centralizes the demographic,

For peer review only

diagnostic, obstetric and neonatal clinical data of mothers and their offspring. In addition, it incorporates the systematic collection of information on the use of vaccines (tetanus/diphtheria, Tdap, influenza, rubella, hepatitis B, hepatitis A), as well as information on dosage, date of vaccination and time of vaccination (before or during pregnancy, or after delivery).(37) In response to the outbreak of the COVID-19 pandemic, CLAP developed a specific SIP form to monitor COVID vaccination of pregnant people, along with other related variables, such as COVID-19 history, which have become part of the routine follow-up of pregnant people (Supplemental material II).

Therefore, this project aims to retrospectively describe the use of COVID-19 vaccines among pregnant people and explore the risk associated with vaccination and selected adverse events, adapting the SIP-PLUS database for surveillance of the use and safety of COVID-19 vaccines from data routinely collected during obstetric and neonatal monitoring of pregnant people in a network of Latin American centers.

GENERAL AND SPECIFIC OBJECTIVES.

General objective.

To describe the utilization of COVID-19 vaccines by pregnant people in hospitals using the SIP-PLUS database and to analyze the risk of vaccination associated with selected maternal and neonatal events.

Specific objectives.

1. To describe the proportion of vaccinated individuals among pregnant people by type of COVID-19 vaccine, number of doses administered, and country.

2. Describe the frequency (Cumulative Incidence) of the following neonatal and maternal events in the cohort of pregnant people: preterm delivery, small for gestational age, low birth.

3. To analyze the associated risk of vaccination for the following neonatal events of interest through an exploration of case-controls nested in a cohort for the following events: prematurity, small for gestational age, and low birth weight.

For peer review only - http://bmjopen.bmj.com/site/about/guidelines.xhtml

Methods and Analysis

1. Design and rationale

Retrospective observational descriptive cohort and analytical study with a nested case-control approach. All outcome and exposure data will be available before the study start date (retrospective analysis).

The dedicated module for capturing COVID-19 vaccine exposure information (including possible heterologous combinations) will be used to obtain the data required. This module complements the current data routinely collected for the vaccines recommended during pregnancy and is collected as part of the obstetric clinical monitoring (Supplemental material II).

2. Source of information and study period.

This study includes secondary data. All the necessary information, including socio-demographic data, comorbidities, exposure to vaccines and events will be retrieved from the SIP-PLUS perinatal electronic medical record database that electronically consolidates perinatal medical record data obtained during routine clinical practice over a retrospective 12-month period.

3. Population, outcome variables, exposure and others.

3.1 Retrospective description of vaccination and event frequency in the pregnant people cohort.

3.1.1 Inclusion criteria

All pregnant people admitted for delivery (singleton) or miscarriage from October 2022 to July 2023.

3.1.2 Exclusion criteria

Twin pregnancies will be excluded.

3.1.3 Adverse events of interest

1. Premature births (preterm delivery)
   Born alive at less than 37 weeks gestational age as registered in the SIP form Clinical record.

For peer review only

2. Small for gestational age. Live birth classified as small by country standards.
3. Low birth weight. Born alive weighing less than 2500 grams.
4. Stillbirth (at any time).
5. Non-extended neonatal death (max 48h postpartum).
6. Congenital malformations (major and minor).
7. Maternal death (at any time).
8. Maternal Near Miss (MNM). Pregnant people with a MNM condition according to WHO MNM approach.(38)
9. Abortion (spontaneous abortion and therapeutic abortion)

3.1.4 Covariates

Sociodemographic variables:

- Mother's age (years)
- Ethnicity (white, indigenous, mixed, other)
- Marital status (married, common-law marriage, single, other)
- Educational level (none, elementary, high school, college, university)
- Country
- Hospital

Obstetric variables and risk factors (definitions)

- Date of last menstrual period
- Gestational age (weeks)
- Smoking (yes/no)
- Drug use (yes/no)
- Alcoholism (yes/no)
- Induced labor (yes/no)
- Diabetes
    - Pre-existing (yes/no)
    - Gestational (yes/no)
- High blood pressure
    - Pre-existing (yes/no)
    - Gestational (yes/no)

For peer review only - http://bmjopen.bmj.com/site/about/guidelines.xhtml

- Eclampsia (yes/no)
- Preeclampsia (yes/no)
- Parity (nulliparous, multiparous)
- Inter-gestational period (months)
- COVID infection (describe diagnosis, prior to or during pregnancy)

Administration of other recommended vaccines during pregnancy

- Tetanus/diphtheria (yes/no)
- Tdap (yes/no)
- Hepatitis B (yes/no)
- Influenza (yes/no)
- Any of the other recommended vaccines (yes/no)

3.1.5 Exposure of interest (immunization with COVID-19 vaccines)

Type of vaccine, number of doses administered, and date.

The vaccines of interest will be(33)

-Astra-Zeneca (AZD1222; ChAdOx1 nCoV-19; ChAdOx1-S; Vaxzevria, Covishield)

-Janssen (NJ-78436735; Ad26.COV2-S)

-Moderna (mRNA-1273; Elasomeran)

-Pfizer-BioNTech (BNT162b2; Tozinameran; Comirnaty)

-Sinopharm (Sinopharm/BIB)

-Sinovac (CoronaVac; VeroCell)

-Sputnik- Gamaleya (Gamaleya; Gam-COVIDVac; Adeno-based (rAd26-S+rAd5-S)

The rest of vaccines not considered in the list will be recorded in free text as "other vaccines". When the administration of two different vaccines is included, the vaccination will be classified as "heterologous schedule". On the other hand, it will be considered a complete schedule if the pregnant person received 2 doses (or 1 dose in case of Janssen’s vaccine) and a booster schedule if she has received 3 doses (or 2 doses in case of Janssen’s vaccine).

For peer review only

3.2. Analysis of the risk associated with vaccination for the occurrence of the neonatal events of interest through an exploration of case-controls nested in a cohort.

A matched case-control approach nested in a cohort will be carried out for each of the events of interest.

3.2.1 Cohort definition

- Pregnant people (pre-term to late term) and with mother's age data

- Live births with delivery date available based on the best available obstetric estimator.

- Perinatal medical record with "COVID-19 vaccine module" implemented.

3.2.2 Case definition (Events of interest)

1. Premature. Preterm delivery: live birth at less than 37 weeks gestational age according to the best available obstetric estimator
2. Small for gestational age: live birth classified as small based on country standards.
3. Low birth weight: born alive weighing less than 2500 grams.

3.2.3 Definition of controls

Two groups of three (uneventful) controls will be randomly selected for each case. The first group of controls will be randomly selected at the same delivery date of each case matched by delivery hospital and mother's age (+/- 3 years). The second group of controls will be births randomly selected from the estimated conception time of the cases based on the estimated delivery date (EDD). EDD will be estimated according to the best available estimator considering the following characteristics: 1) Early ultrasound; 2) Clinical (last menstrual period –LMP-). If EDD is not available, gestational age considered during newborn examination should be considered. Controls will be also matched by delivery hospital and mother's age (+/- 3 years).

3.2.4 Definition of exposure. Vaccines considered will be those mentioned in section 3.1.5.

Exposure shall be classified according to the following exposure windows**:**

For peer review only - http://bmjopen.bmj.com/site/about/guidelines.xhtml

1. **"Late immunization**": pregnant people immunized on the index date or up to one trimester before the end of pregnancy (third trimester)**.**
2. **Second trimester immunization":** pregnant people immunized after completing the first trimester until the second trimester before the event.
3. **"First trimester immunization"** pregnant people immunized during the first trimester.
4. **"Past immunization"** immunized prior to pregnancy
5. **"Immunization at any time".** Immunization at any of the above times**.**

Moreover, gestational age (weeks) will be considered if available (Supplementary material 2)

The figure 1 summarizes the overall conceptual framework and procedures of this study.

4. Expected sample size and rationale for its determination.

4.1 General considerations

In estimating the sample size, the frequencies of events observed in a previous analysis in the SIP-PLUS database were considered to estimate the number of potential events (Supplemental material III). Given the lack of precise data on vaccination coverage in hospitals in the CLAP network, literature values from studies published in other countries (prevalences ranging from 20 to 50%) were used as a reference.(27,28) The design of the contrast tests as bilateral (or two-tailed) is because the hypothesis contemplates the scenario of a possible protection against adverse events associated with vaccines and also the opposite scenario, with an increased risk. The correlation coefficient (r) between (matched) cases and controls is not known and cannot be estimated from previous studies. Hence, a conservative value of "r=0.2" has been assumed for the calculation of the sample size, following the recommendation of some authors for these cases.(39) In this part we will consider the analysis of events for which, a priori, there would be sufficient statistical power, anticipating that the measures of association (Odds Ratio) may not be to large based on existing published studies.

4.2. Main analysis of vaccination (any vaccine) with respect to "non-use".

For peer review only - http://bmjopen.bmj.com/site/about/guidelines.xhtml

For the matched case-control study (matched with 3 controls) with a power of 80%, alpha error=0.05 (bilateral), a case-control correlation coefficient of 0.2 and a vaccine exposure of 20% would require at least 1009 cases (3,027 controls) to detect an increased probability of vaccine-associated event risk of 30% (OR=1.3) and at least 650 cases (1,950 controls) to detect 30% protection (OR=0.7) (Figures 2 and 3).

4.3. Estimation of the expected number of cases in the database

Based on previous estimates in the SIP-PLUS database and the annual number of deliveries in each hospital of the network, enough cases could be expected annually: 5,731 cases of small for gestational age, 10,932 preterm, and 5,995 low birth weight babies.

5. Data analysis

5.1 Retrospective description (Cumulative Incidence) of the following maternal and neonatal events in the pregnant cohort: preterm, small for gestational age, low birth weight, stillbirth, neonatal death, congenital malformations, maternal near miss (MNM) and maternal death.

The cumulative incidence will be estimated together with the 95% confidence interval using the exact method. When calculating the cumulative incidence, the number of events of interest will be used in the numerator and different denominators according to the type of event.

1. "Live births" denominator: small for gestational age, premature, low birth weight, stillbirth, MNM, and maternal death.

2. "Total pregnancies" denominator: stillbirth, congenital malformations.

5.2 Description of the proportion of pregnant people who have received a dose of COVID-19 vaccine (total and by type of vaccine) and complete vaccination schedule.

The proportion of vaccinated pregnant people out of the total number of pregnant people in the database will be estimated. The proportions (prevalences) of immunization will be estimated by type of vaccine, vaccine platform (ARNm, Adenoviral vector-based), schedule (complete/incomplete) and time of administration (pre-

For peer review only - http://bmjopen.bmj.com/site/about/guidelines.xhtml

pregnancy, first trimester, second trimester, third trimester). The total prevalence of maternal immunization (combination of all vaccines administered at any time) will also be estimated. The 95% confidence interval of the proportion will be calculated using the exact method.

5.3 Nested case-control analysis

Logistic regression models will be built for each event of interest, considering COVID-19 vaccine exposure as the main independent variable adjusting for possible confounders (covariates) and estimating the Odds Ratio together with its 95% confidence intervals (CI) as a measure of association (OR). The use of a "standard" (non-conditional conditional) logistic regression analysis will consider adjustment for potential confounders and in addition to adjustment variables, but in addition matched variables will be included in the adjusted models, as recommended for the minimization of bias in the analysis of matched case-control studies.(40) Models will include: (1): (1) unadjusted models with only the matching variables (mother's age, date of delivery or conception time, and hospital) and (2) a fully adjusted models that also includes all possible confounders described above. The case control analyses will follow two approaches depending on the control group: 1) Approach A will consider the randomly selected sample of controls from deliveries matched on the same birth date, maternal age and attended in the same hospital and 2) Approach B will consider the controls randomly selected based on the best estimate of the conception time (LMP or early ultrasound and the due date), same maternal age and attended in the same hospital. (Supplementary material IV) As a rule, missing variable data will be treated as a separate (unknown) category. If necessary, missing data will be handled by multiple imputation methods.

Analyses by individual vaccine type or platform will be explored. Sensitivity analysis will be performed for different exposure time windows considering the different types of exposure considered in the paper, and their classification according to proximity to the event (late vaccination, second trimester, early), completeness of the schedule (complete or incomplete schedule) and country. Sample size estimation were made using PS: Power and Sample Size Calculation (v. 3.1.6).(41) Statistical analyses will be performed using Stata 17 statistical software (Stata Corp., College Station, TX).

For peer review only - http://bmjopen.bmj.com/site/about/guidelines.xhtml

Patient and public involvement.

This protocol presents a retrospective register-based, non-experimental study, and will not have measures of intervention that could burden patients in any way assessable not constituting any risk for patients' participation, and it neither implies the realization of diagnostic tests or additional therapeutic decision besides of what the professional considers timely necessary as part of their clinical practice. Besides, the research question formulation was based upon our own priorities for patient benefit and result interpretation. The nested case–control study described by the protocol is based on clinical records from a network of hospital sites and thus will not include a recruitment process for patients. There was no patient or public involvement in the design of the current study protocol nor conduct of the study. However, results will be distributed to pregnant people groups, as well as peer-reviewed journals and scientific conferences.

Strengths and Limitations

Risk of indication bias. It is possible that women at higher baseline risk for severe COVID-19 may be more likely to have been vaccinated than those not presenting these factors. Adjusted models will consider inclusion of risk factors (e.g., diabetes, hypertension, BMI≥30 kg/m2) as adjustment variables. (4,42)

Availability of controls. The number of controls available for case-matching may be small. In field studies, obtaining controls is very expensive and getting the desired number may turn out unfeasible. However, in database studies controls are more readily available. Nevertheless, in the exceptional case of lack of sufficient controls. A contingency plan will be carried out considering the following scenarios. Scenario 1. Widening the controls' maternal age to +/- 5 years (keeping the same hospital and delivery date or due date); 2) scenario, delivery date or due date to one week (maintaining maternal age +/- 3 and same hospital). Since we will use two different approaches for control selection, the analysis from both perspectives will allow to test the consistency of the results.

Possible lack of "non-exposure" to COVID-19 vaccines in pregnant people (case of massive recommendation for maternal immunization and full coverage). We understand that current recommendations are aimed at promoting maternal immunization with COVID-19 vaccines, but full coverage is not anticipated at present. However, an interim analysis is planned at the end of the year to assess the prevalence

For peer review only

of exposure by that time, and to readjust the initial estimates of sample size and study potential before the start of the study.

Missing data. As in any study with secondary data, there are always limitations due to incomplete data records, inaccuracies (dates), unmeasured confounding factors, and missing data, etc. In case of missing data for a variable, these will be treated as a separate category in the analysis, and multiple imputation techniques will be considered whenever necessary.

Moreover, the description of abortions will be considered exploratory/hypothetical since: 1) it is not possible to differentiate whether the abortions recorded are spontaneous or induced and, 2) a significant proportion of spontaneous abortions occurs on the first weeks of pregnancy and they may not require hospital care nor attended in maternal hospital settings.

Finally, Randomized Clinical Trials (RCTs) are the gold-standard for causal inference. However comparative observational studies may be appropriate when RCTs are not feasible. With the new recommendations for maternal immunization issued by the CDC and WHO RCTs comparing COVID-19 vaccines vs placebo would be difficult to carry out since clinical equipoise will not be reached and administering placebo would be considered unethical. In this scenery retrospective observational studies can provide comparative evidence on COVID-19 vaccine effects versus unexposed pregnant people. Furthermore, information on COVID-19 vaccine safety in pregnant people might mainly be coming, as already mentioned, from high-income countries (HICs). This study has the strength of generating evidence of COVID 19 vaccines which are those currently being used in LAC, asides from mRNA. It represents an opportunity to produce complementary evidence of maternal, foetal and neonatal adverse events from hospitals in middle- and low-income settings but also to gather information for vaccines whose safety profile in pregnancy is less known such as vaccines non authorized nor utilized in HICs.

Ethics and dissemination

The present proposal will be implemented in compliance with the principles of the Declaration of Helsinki.(43) and the international standards for the conduct of epidemiological studies, contained in the International Guidelines for Ethical Review of Epidemiological Studies.(44) The reporting of results will conform to the guidelines for

For peer review only

reporting routinely collected and observational pharmacoepidemiology health data (RECORD-PE).(45)The study protocol was reviewed by the Ethical Review Committee on Research of the Panamerican Health Organization. Patient informed consent was waived due to the retrospective design of the study and the utilization of anonymized data (Ref. No: PAHOERC.0546.01). Study results will be disseminated in Open Access journals.

This is an open access article distributed in accordance with the Creative Commons Attribution 4.0 International (CC BY 4.0) license, which permits others to copy and redistribute, remix, adapt, build upon this work for any purpose, even commercially, giving appropriate credit.

Benefit-risk assessment for participants

This retrospective study based on secondary data does not entail any additional risk for the patients because of their participation. Nor does it involve additional diagnostic tests, evaluation or therapeutic decisions beyond those deemed appropriate as part of routine clinical practice. However, the results of the proposal are expected to inform the understanding of the benefit-risk balance of maternal immunization against COVID-19 in the future.

Confidential treatment of data.

As the study will be carried out on an anonymized database without personal identifiers, an informed consent waiver was granted.

Authors' contributions

Diego Macías Saint-Gerons, Alba Maria Ropero, Bremen de Mucio, Claudio Sosa, José Luis Castro, Suzanne Serruya, and Mercedes Colomar wrote the proposal. Desiré Pastor, Monica Chiu, Martha Velandia, Robin Rojas, Edgardo Abalos, Pablo Durán, and Rodolfo Gómez Ponce de León reviewed the proposal and made remarkable contributions. Giselle Tomasso, Luis Mainero and Marcelo Rubino designed the clinical records. All the authors reviewed and agree with the protocol.

For peer review only

Funding statement

This study is funded by Health Canada: Grant Number: 452029: "Access to COVID-19 Vaccines and Therapeutics for Populations in Situations of Vulnerability in the Americas and to support maternal immunization safety related activities"; U.S. Food and Drug Administration grant to support maternal immunization safety related activities, and by the US government (USG) funds through the American Rescue Plan Act.

Competing interest statement.

The authors declare that they have no known competing financial interests or personal relationships that could have appeared to influence the work reported in this paper. The opinions expressed in this article are those of the authors and do not necessarily reflect the views of the Pan American Health Organization (PAHO), its Board of Directors, or the countries they represent.

Figure Legends

Figure 1. Overview of the study procedure for data collection and outcomes of interest

Figure 2. Power of the analysis as a function of the number of cases for different probabilities of exposure to vaccines from 20% to 50% for the detection of an OR=1.3 (increased risk).

Figure 3. Power as a function of the number of cases for different probabilities of exposure to vaccines from 20% to 50% for the detection of an OR=0.7 (protection).

For peer review only - http://bmjopen.bmj.com/site/about/guidelines.xhtml

References

1. WHO SAGE Roadmap for Prioritizing Uses Of COVID-19 Vaccines in The Context of Limited Supply - An approach to inform planning and subsequent recommendations based on epidemiological setting and vaccine supply scenarios. [online]. 2021. https://reliefweb.int/report/world/who-sage-roadmap-prioritizing-uses-covid-19-vaccines-context-limited-supply-approach (Accessed 16 July 2021).

2. Wei SQ, Bilodeau-Bertrand M, Liu S, et al. The impact of COVID-19 on pregnancy outcomes: a systematic review and meta-analysis. CMAJ: Canadian Medical Association. 2021 Apr;193(16):E540–8. doi: 10.1503/cmaj.202604

3. Adhikari EH, Spong CY. COVID-19 Vaccination in Pregnant and Lactating Women. JAMA. 2021 Mar 16;325(11):1039–40. doi: 10.1001/jama.2021.1658

4. Allotey J, Stallings E, Bonet M, et al. Clinical manifestations, risk factors, and maternal and perinatal outcomes of coronavirus disease 2019 in pregnancy: living systematic review and meta-analysis. BMJ (Clinical research ed). 2020 Sep;370. doi: 10.1136/bmj.m3320

5. Di Mascio D, Sen C, Saccone G, et al. Risk factors associated with adverse fetal outcomes in pregnancies affected by Coronavirus disease 2019 (COVID-19): a secondary analysis of the WAPM study on COVID-19. Journal of perinatal medicine. 2020 Jan 1;49(1):111–5. doi: 10.1515/jpm-2020-0355

6. Ciapponi A, Bardach A, Comandé D, et al. COVID-19 and pregnancy: An umbrella review of clinical presentation, vertical transmission, and maternal and perinatal outcomes. PloS one. 2021 Jun 1;16(6). doi: 10.1371/journal.pone.0253974

7. Akhtar H, Patel C, Abuelgasim E, et al. COVID-19 (SARS-CoV-2) Infection in Pregnancy: A Systematic Review. Gynecologic and obstetric investigation. 2020 Oct 1;85(4):295–306. doi: 10.1159/000509290

For peer review only

8. Beigi RH, Krubiner C, Jamieson DJ, et al. The need for inclusion of pregnant women in COVID-19 vaccine trials. Vaccine. 2021 Feb 5;39(6):868–70. doi: 10.1016/j.vaccine.2020.12.074

9. Macias Saint-Gerons D, Solà Arnau I, De Mucio B, et al. Adverse events associated with the use of recommended vaccines during pregnancy: An overview of systematic reviews. Vaccine. 2021 Jul;39 Suppl 2:B12–26. doi: 10.1016/j.vaccine.2020.07.048

10. Collier ARY, McMahan K, Yu J, et al. Immunogenicity of COVID-19 mRNA Vaccines in Pregnant and Lactating Women. JAMA. 2021 Jun 15;325(23):2370–80. doi: 10.1001/jama.2021.7563

11. Beharier O, Mayo RP, Raz T, et al. Efficient maternal to neonatal transfer of antibodies against SARS-CoV-2 and BNT162b2 mRNA COVID-19 vaccine. The Journal of clinical investigation. 2021 Oct 1;131(19). doi: 10.1172/JCI154834

12. Bowman CJ, Bouressam M, Campion SN, et al. Lack of effects on female fertility and prenatal and postnatal offspring development in rats with BNT162b2, a mRNA-based COVID-19 vaccine. Reproductive toxicology (Elmsford, NY). 2021 Aug 1;103:28–35. doi: 10.1016/j.reprotox.2021.05.007

13. Shimabukuro TT, Kim SY, et al. Preliminary Findings of mRNA Covid-19 Vaccine Safety in Pregnant Persons. New England Journal of Medicine. 2021 Jun 17;384(24):2273–82. doi: 10.1056/NEJMoa2104983

14. Covas DT, de Jesus Lopes de Abreu A, Zampirolli Dias et al. Adverse events of COVID-19 vaccines in pregnant and postpartum women in Brazil: A cross-sectional study. PloS one. 2023;18(1):e0280284. doi: 10.1371/journal.pone.0280284

15. DeSilva M, Haapala J, Vazquez-Benitez G, et al. Evaluation of Acute Adverse Events after Covid-19 Vaccination during Pregnancy. Vol. 387, The New England journal of medicine. United States; 2022. p. 187–9. doi: 10.1056/NEJMc2205276

16. Goldshtein I, Steinberg DM, Kuint J, et al. Association of BNT162b2 COVID-19 Vaccination During Pregnancy With Neonatal and Early Infant

For peer review only

Outcomes. JAMA pediatrics. 2022;176(5). doi: 10.1001/jamapediatrics.2022.0001

17. Kachikis A, Englund JA, Singleton M, et al. Short-term Reactions Among Pregnant and Lactating Individuals in the First Wave of the COVID-19 Vaccine Rollout. JAMA network open. 2021;4(8). doi: 10.1001/jamanetworkopen.2021.21310

18. Magnus MC, Örtqvist AK, Dahlqwist E, et al. Association of SARS-CoV-2 Vaccination During Pregnancy With Pregnancy Outcomes. JAMA. 2022 Apr 19;327(15):1469. doi: 10.1001/jama.2022.3271

19. Zauche LH, Wallace B, Smoots AN, et al. Receipt of mRNA Covid-19 Vaccines and Risk of Spontaneous Abortion. The New England journal of medicine. 2021 Oct 14;385(16):1533–5. doi: 10.1056/NEJMc2113891

20. Falsaperla R, Leone G, Familiari M, et al. COVID-19 vaccination in pregnant and lactating women: a systematic review. Expert review of vaccines. 2021;20(12):1619–28. doi: 10.1080/14760584.2021.1986390

21. Lipkind HS, Vazquez-Benitez G, DeSilva M, et al. Receipt of COVID-19 Vaccine During Pregnancy and Preterm or Small-for-Gestational-Age at Birth - Eight Integrated Health Care Organizations, United States, December 15, 2020-July 22, 2021. MMWR. Morbidity and mortality weekly report. 2022 Jan;71(1):26–30. doi: 10.15585/mmwr.mm7101e1.

22. Magnus MC, Gjessing HK, Eide HN, et al. Covid-19 Vaccination during Pregnancy and First-Trimester Miscarriage. The New England journal of medicine. 2021 Nov 18;385(21):2008–10. doi: 10.1056/NEJMc2114466

23. Ibroci E, Liu X, Lieb W, et al. Impact of prenatal COVID-19 vaccination on delivery and neonatal outcomes: Results from a New York City cohort. Vaccine. 2023 Jan;41(3):649–56. doi: 10.1016/j.vaccine.2022.09.095

24. Prasad S, Kalafat E, Blakeway H, et al. Systematic review and meta-analysis of the effectiveness and perinatal outcomes of COVID-19 vaccination in pregnancy. Nature communications. 2022 Dec 1;13(1). doi: 10.1038/s41467-022-30052-w

For peer review only

25. Magee LA, von Dadelszen P, Kalafat E, et al. COVID-19 vaccination in pregnancy-number needed to vaccinate to avoid harm. The Lancet Infectious diseases. 2021 Dec 1;21(12):1627. doi: 10.1016/S1473-3099(21)00691-5

26. Kalafat E, Magee LA, von Dadelszen P, et al. COVID-19 booster doses in pregnancy and global vaccine equity. Lancet (London, England). 2022 Mar 5;399(10328):907–8. doi: 10.1016/S0140-6736(22)00166-0

27. Stock SJ, Carruthers J, Calvert C, et al. SARS-CoV-2 infection and COVID-19 vaccination rates in pregnant women in Scotland. Nature medicine. 2022 Mar 1;28(3):504–12. doi: 10.1038/s41591-021-01666-2

28. Engjom H, van den Akker T, Aabakke A, et al. Severe COVID-19 in pregnancy is almost exclusively limited to unvaccinated women - time for policies to change. The Lancet regional health Europe. 2022 Feb 1;13. doi: 10.1016/j.lanepe.2022.100313

29. Kharbanda EO, Haapala J, Desilva M, et al. Spontaneous Abortion Following COVID-19 Vaccination During Pregnancy. JAMA. 2021 Oct 26;326(16):1629–31. doi: 10.1001/jama.2021.15494

30. Centers for disease control and prevention (CDC). COVID-19 Vaccines While Pregnant or Breastfeeding [online]. 2022. https://www.cdc.gov/coronavirus/2019-ncov/vaccines/recommendations/pregnancy.html (Accessed 23 Nov 2022)

31. WHO. Questions and Answers: COVID-19 vaccines and pregnancy. [online] 2022. https://www.who.int/publications/i/item/WHO-2019-nCoV-FAQ-Pregnancy-Vaccines-2022.1 (Accessed 22 Sep 2022)

32. DeSilva MB, Haapala J, Vazquez-Benitez G, et al. Medically Attended Acute Adverse Events in Pregnant People After Coronavirus Disease 2019 (COVID-19) Booster Vaccination. Obstetrics and gynecology. 2023 Jul;142(1):125–9. doi: 10.1097/AOG.0000000000005241

33. PAHO. Pharmacovigilance for COVID-19 vaccines [online]. 2023. https://covid-19pharmacovigilance.paho.org/ (Accessed 7 Jul 2023).

For peer review only - http://bmjopen.bmj.com/site/about/guidelines.xhtml

34. Kalafat E, O'Brien P, Heath PT, et al. Benefits and potential harms of COVID-19 vaccination during pregnancy: evidence summary for patient counseling. Ultrasound in obstetrics & gynecology: the official journal of the International Society of Ultrasound in Obstetrics and Gynecology. 2021 May 1;57(5):681–6. doi: 10.1002/uog.23631

35. Stafford IA, Parchem JG, Sibai BM. The coronavirus disease 2019 vaccine in pregnancy: risks, benefits, and recommendations. American journal of obstetrics and gynecology. 2021 May 1;224(5):484–95. doi: 10.1016/j.ajog.2021.01.022

36. Blakeway H, Prasad S, Kalafat E, et al. COVID-19 vaccination during pregnancy: coverage and safety. American journal of obstetrics and gynecology. 2022 Feb 1;226(2):236.e1-236.e14. doi: 10.1016/j.ajog.2021.08.007

37. Gomez Ponce de Leon R, Baccaro LF, Rubio Schweizer G, et al. Building a network of sentinel centres for the care of women in an abortion situation: advances in Latin America and the Caribbean. BMJ global health. 2022 Oct;7(10). doi: 10.1136/bmjgh-2022-010059

38. Organization WH. Evaluating the quality of care for severe pregnancy complications: the WHO near-miss approach for maternal health. Geneva: WHO Press; 2011.

39. Dupont WD. Power Calculations for Matched Case-Control Studies. Biometrics. 1988 Dec;44(4):1157.

40. Pearce N. Analysis of matched case-control studies. BMJ. 2016 Feb 25;352. doi: 10.1136/bmj.i969.

41. Dupont WD, Plummer WDJ. Power and sample size calculations. A review and computer program. Controlled clinical trials. 1990 Apr;11(2):116–28. doi: 10.1016/0197-2456(90)90005-m.

42. Zambrano LD, Ellington S, Strid P, et al. Update: Characteristics of Symptomatic Women of Reproductive Age with Laboratory-Confirmed SARS-CoV-2 Infection by Pregnancy Status — United States, January 22–October 3, 2020.

For peer review only

MMWR Morbidity and Mortality Weekly Report. 2020 Nov;69(44):1641–7. doi: 10.15585/mmwr.mm6944e3.

43. World Medical Association Declaration of Helsinki: ethical principles for medical research involving human subjects. JAMA. 2013 Nov 27;310(20):2191–4.

44. CIOMS. International Ethical Guidelines for Epidemiological Studies. Geneva; 2009. 130 p.

45. Langan SM, Schmidt SA, Wing K, et al. The reporting of studies conducted using observational routinely collected health data statement for pharmacoepidemiology (RECORD-PE). BMJ (Clinical research ed). 2018;363. doi: 10.1136/bmj.k3532.

For peer review only

TABLES

Table 1. Characteristics of the hospitals that use the SIP-PLUS database

Country	Income Level	Name of Hospital	Level	Deliveries attended per year
Argentina	UMIC	Martin Maternity Hospital, Rosario	Tertiary	3600
		Roque Sáenz Peña, Rosario		2500
		Eva Perón, Rosario		1500
Bolivia	LMIC	Bolivian-Japanese Hospital	Tertiary	2719
		La Paz Maternity Hospital	Tertiary	1500
Colombia	UMIC	Rafael Calvo Maternity Clinic	Secondary	7000
		Hospital Universitario del Valle, Cali	Tertiary	7200
Ecuador	UMIC	Isidro Ayora Maternity Hospital	Tertiary	7000
Honduras	LMIC	Roberto Suazo Cordova Hospital	Secondary	3201
		San Felipe Hospital	Secondary	6128
		Leonardo Martínez Valenzuela	Tertiary	18000
Nicaragua	LMIC	España Hospital	Secondary	5250
		Berta Calderón Roque Hospital	Secondary	8993
Dominican Republic	UMIC	San Lorenzo de Los Mina Hospital	Tertiary	13000
Uruguay	UMIC	Pereira Rosell Hospital Center	Tertiary	5000
		Hospital de Clinicas Manual Quintela	Tertiary	800

LMIC: low- and middle-income country; UMIC: upper-middle-income country.

For peer review only

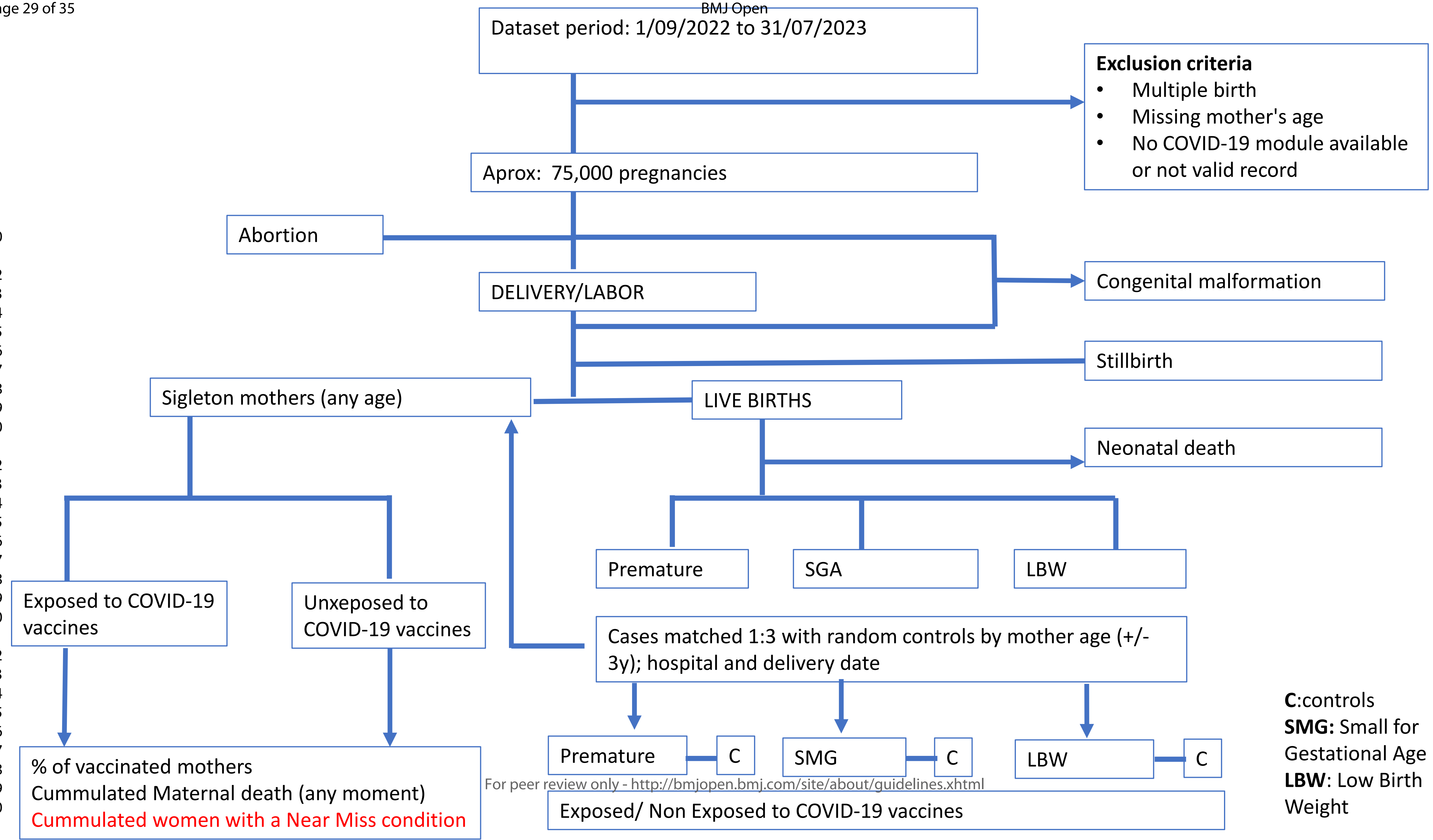
Dataset period: 1/09/2022 to 31/07/2023
Exclusion criteria
Multiple birth
Missing mother's age
No COVID-19 module available or not valid record
Aprox: 75,000 pregnancies
Abortion
DELIVERY/LABOR
Congenital malformation
Stillbirth
Sigleton mothers (any age)
LIVE BIRTHS
Neonatal death
Premature
SGA
LBW
Exposed to COVID-19 vaccines
Unexposed to COVID-19 vaccines
Cases matched 1:3 with random controls by mother age (+/- 3y); hospital and delivery date
Premature
C
SMG
C
LBW
C
% of vaccinated mothers
Cummulated Maternal death (any moment)
Cummulated women with a Near Miss condition
Exposed/ Non Exposed to COVID-19 vaccines
C:controls
SMG: Small for Gestational Age
LBW: Low Birth Weight

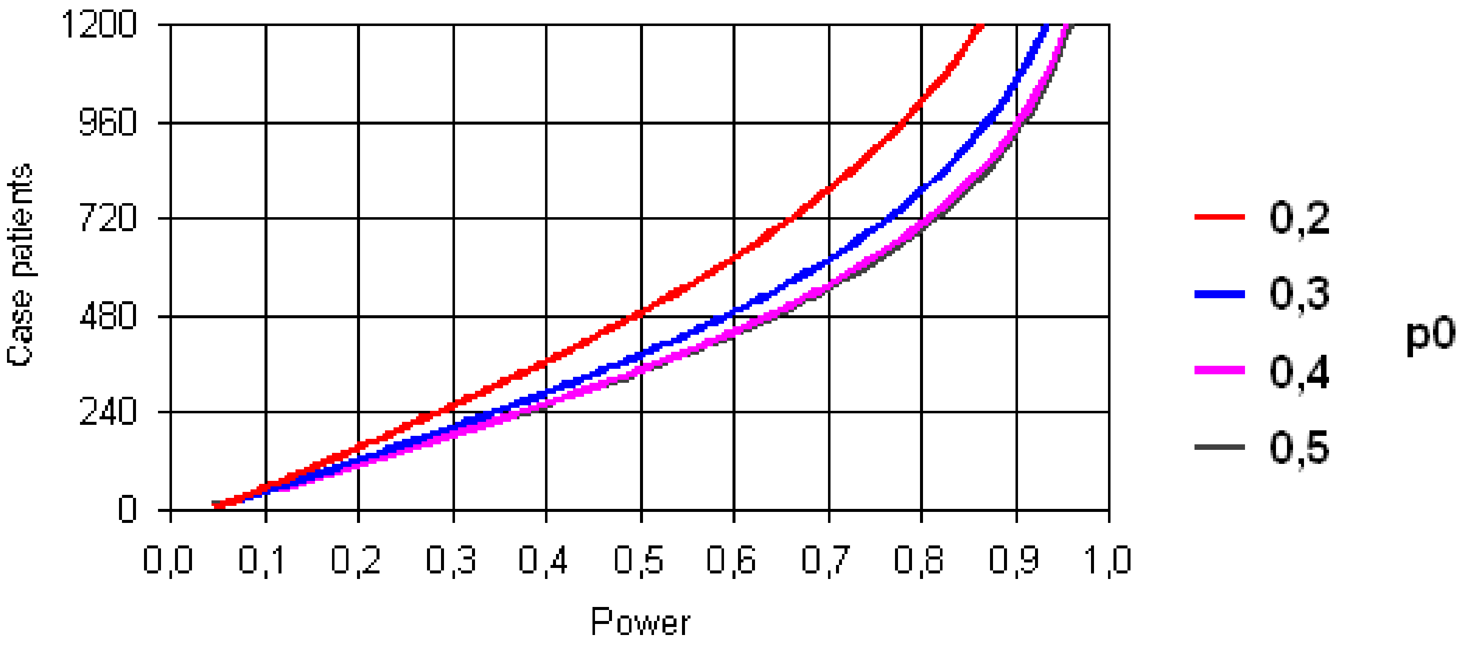
1200
960
720
480
240
0
Case patients
0,0
0,1
0,2
0,3
0,4
0,5
0,6
0,7
0,8
0,9
1,0
Power
0,2
0,3
0,4
0,5
p0

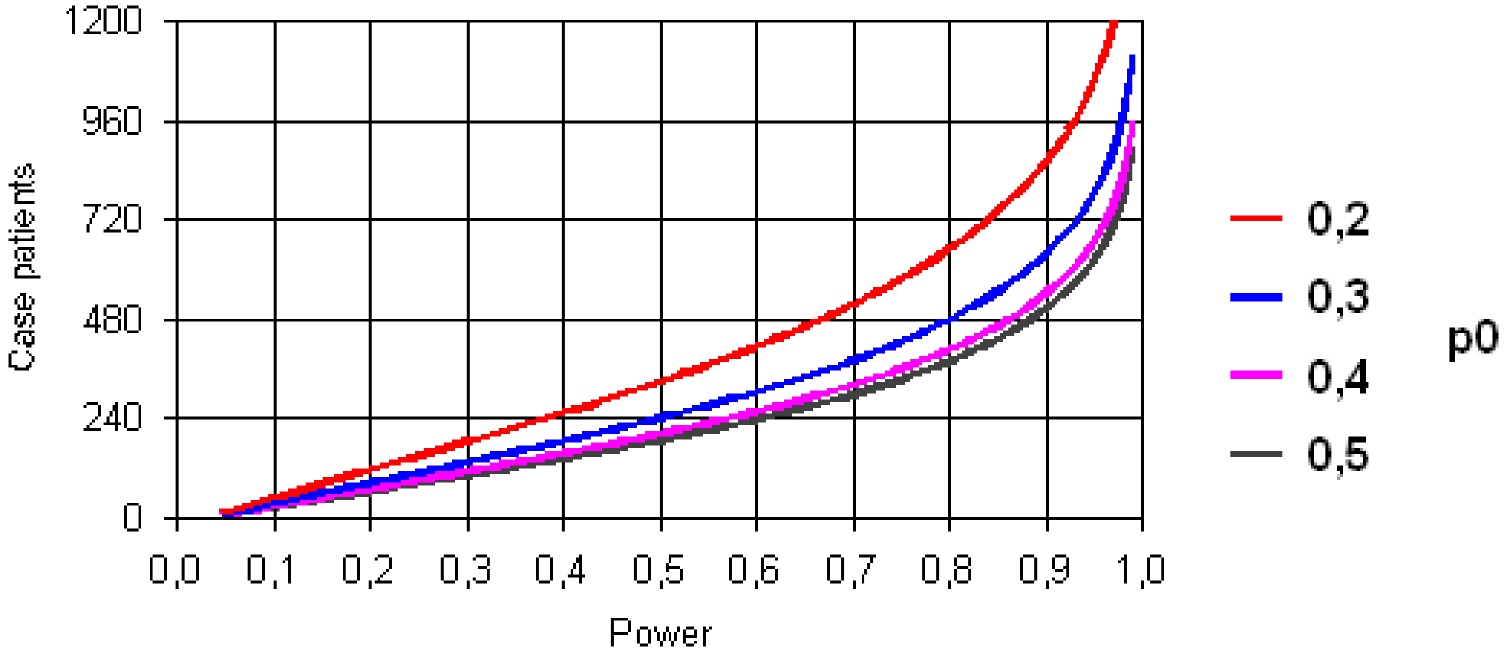
1200
960
720
480
240
0
Case patients
0,0
0,1
0,2
0,3
0,4
0,5
0,6
0,7
0,8
0,9
1,0
Power
0,2
0,3
0,4
0,5
p0

PERINATAL CLINICAL RECORD - CLAP/WR-PAHO/WHO

FIRST NAME | LAST NAME

ADDRESS

DISTRICT | PHONE.

DATE OF BIRTH: day month year

AGE (years): < 15, > 35

ETHNIC RACY: white, indigenous, mixed, black, other

LITERACY: no, yes

EDUCATION: none, primary, secondary, university; years in highest level

CIVIL STATUS: married, common mar., single, other; lives alone: no, yes

Place of antenatal visits

Place of birth

ID #

This color means WATCH (does not necessarily indicate risk or inadequate practices)

HISTORY

FAMILY (no, yes) | PERSONAL (no, yes): TBC, diabetes (I, II, G), hypertension, pre-eclampsia, eclampsia, other severe med. cond.

surgery of UT-UG, infertility, heart dis., kidney dis., violence, HIV+ (no, yes)

OBSTETRIC: prev. gest, abortions, vaginal, livebirths, alive

LAST PREVIOUS: n/a, < 2500g, normal, ≥ 4000g

History of twins: no, yes

3 consec. spont. ab.; tubal pregnancy; deliveries; cesar. sections; stillbirths; died 1st week; died after 1st week

END PREVIOUS PREGNANCY: day, month, year; < 1 year

PLANNED PREGNANCY: no, yes

CONTRACEP. METHOD FAILURE: no method, barrier, IUD, horm., emerg., natural

CURRENT PREGNANCY

PREVIOUS WEIGHT (Kg) | HEIGHT (cm)

DLM: day month year | EDD: day month year

GA BASED on: DLM, Us <20 w.; no, yes

ACT SMO, PAS SMO, DRUGS, ALCOHOL, VIOLENCE (no, yes): 1st trim, 2nd trim, 3rd trim

VACCINES: no, yes before pregnancy, yes during pregnancy, yes after delivery/abortion; day, month, year; number of doses; gest. age weeks

tetanus diphtheria, tdap, influenza, rubella, hepatitis B, hepatitis A

screening hepatitis B: no, yes

NORMAL EX.: DENTAL, BREAST (no, yes)

CERVIX (normal, abnormal, not done): Visual insp, PAP, COLP

GROUP | Rh: −, +; Inmun: no, yes; γglobulina anti D: no, yes, n/a

TOXOPLASMOSIS (−, +, not done): <20 week IgG, ≥20 week IgG, 1 visit IgM

Hb <20 wk: g, <11.0 g/dl

Presciption Fe/Folates: Fe, Folates; no, yes

Hb ≥20 wk: g, <11.0 g/dl

CHAGAS: −, +, not done

MALARIA: −, +, not done

BACTERIURIA (normal, abnormal, not done): wk <20, ≥20

FASTING BLOOD SUGAR: <20 wk mg/dl; ≥30 wk mg/dl

G.B.S: 35-37 weeks; −, +, not done

PREPARING FOR CHILDBIRTH: no, yes

ADVICE ON BREAST-FEEDING: no, yes

HIV - Diag - Treatment: <20 weeks request (yes, no, n/a), test result (+, −, n/d); ART in preg. (yes, no, n/a); ≥20 weeks request (Si, No, n/a), test result (+, −, n/d); ART in preg. (yes, no, n/a)

SYPHILIS Diagnosis and treatment: Test – no treponemic (−, +, n/d), <20 wk; treponemic (−, +, n/d, n/a), weeks; Treatment (no, yes, n/d, n/a), weeks; Treat. partner (no, yes, n/d, n/a); Test – no treponemic (−, +, n/d), ≥20 wk; treponemic (−, +, n/d, n/a), weeks; Treatment (no, yes, n/d, n/a), weeks; Treat. partner (no, yes, n/d, n/a)

PRENATAL VISITS

day	month	year	gest age	weight	BP	uterine height	presentation	FHR (bpm)	fetal movements	proteinuria	place of control	alarm signs, test, treatments	Technician's initials	next visit
				,										
				,										
				,										
				,										
				,										

CHILDBIRTH / ABORTION

DATE OF ADMISSION: day month year

CARD: no, yes

PRENATAL VISITS total

AGE GEST. 1st CONSULT. weeks

HOSPITAL during PREGNANCY: no, yes; days

ANTENATAL STEROIDS: complete, incompl., no, n/a; week started

ONSET: spontan., induced, elect. ces.

ANTEPARTUM RUPT. OF MEMBRANES: no, yes; day month year; hour min; <37 wk, ≥18 hs., temp. ≥38°C

GEST AGE AT CHILDBIRTH: weeks, days; by LMP, by US

PRESENTATION SITUATION: cephalic, breech, transverse

FETAL SIZE ADEQUATE: no, yes

COMPANION: labor, del; couple, relative, other, no one

LABOR

details in partograph: yes, no

hour	min	mother position	BP	pulse	contr./10'	dilatation	height present.	variety posit.	mecon	FHR/dips

DISEASES – Labor Test (−, +, n/d, n/a): Syphilis, HIV; ART: yes, no, n/a

BIRTH

ALIVE; DEAD: antepartum, intrapartum, not known when

hour min; day month year

MULTIPLE: no, yes; order

TERMINATION: spont., forceps, c-sect, vacuum, other

MAIN INDICATION FOR INDUCTION OR SURGERY: code; INDUC., SURG

POSITION LABOR: sitting, recumb, squat

EPISIOTOMY: no, yes

TEARS Grade (1 to 4): no

OXYTOCIC: before expul. plac. (no, yes), after (no, yes)

PLACENTA: complete, retained (no, yes)

EARLY CORD CLAMPING: < 1 min., 1 to 3 min.

MEDICATION RECEIVED: oxytocic at labor, ATB, analges, local anesth, region anesth, gral anesth, transfusion, Mg sulfate preeclam. eclam., others (code) medic 1, medic 2 (no, yes)

NEONATE

SEX: f, m, not defined

BIRTH WEIGHT: g; <2500 g, ≥4000 g

HEAD CIRC cm; LENGTH cm

GESTATIONAL AGE: weeks, days; LMP, US, ESTIMATED

WEIGHT G.A.: adeq., small, large

APGAR (min): 1st, 5th

Early intitiation of breast feeding: no, yes

RESUSCITATION (no, yes): stimul., suction, mask, oxygen, massage, tube, medication

ATTENDED: doctor, obst., nuerse, assist., stud., TBA, other; Name — CHILDBIRTH, NEONATE

CONGENITAL DEFECTS: no, minor, major; code

DISEASES: 1 o more, none; code

NEONATAL SCREENING: HIV in NN – Exposed (no, yes, s/d), Treated (no, yes, s/d, n/a); VDRL (−, +, not done), Tx (no, yes, n/a, s/d); Hearing, Chagas disease, Bili rrub, Toxo IgM, Hb pathy, Cardio-vascular disease (−, +, not done); Metabolic: not done, done

Death at site of birth: Mother, NN (no, yes)

REFERRAL: room in, neonatol, another hosp.

POSTPARTUM

time	Temperature °C	B.P.	pulse	uterine involution	Bleeding	Responsible
	,					
	,					
	,					
	,					
	,					
	,					

NEONATAL DISCHARGE

alive, dead; day month year; hour min

transfer: place

dies during or at transfer: no, yes, s/d

AGE AT DISCHARGE complete day: <1 day

FEEDING AT DISCH: excl. breastf, partial, artificial

Supine: no, yes

BCG: no, yes

INMUN. HEPATITIS B: no, yes

Meconium 1st day: no, yes

WEIGHT AT DISCHARGE

γglobulin anti D: no, yes, n/a

Id. NN | Name NN | Responsabile

HCP NEAR MISS ANV_ENG 02-08-201087

For peer review only

M O R B I L I T Y

HYPERTENSIVE DISORDERS (no / yes)

	no	yes
Chronic hypertension	○	●
Mild preeclampsia	○	●
Severe preeclampsia	○	●
Eclampsia	○	●
HELLP	○	●
Gestational Hypertension	○	●
Chronic hypertension + preeclampsia	○	●

INFECTIONS (no / yes)

	no	yes
Sepsis	○	●
Pyelonephritis	○	●
Pneumonia	○	●
Endometritis	○	●
Caesarean section wound infection	○	●
Chorioamnionitis	○	●
Episiorrhaphy infection	○	●
Asymptomatic bacteriuria	○	●
Other infections	○	●

HEMORRHAGE

1st TRIMESTER (no / yes) 2nd TRIMESTER (no / yes) 3rd TRIMESTER (no / yes)

	no	yes
Post abortion	○	●
Hydatidiform Mole	○	●
Ectopic pregnancy	○	●
Previous placenta	○	●
Accretism	○	●
Abruptio placentae	○	●
Uterine rupture	○	●
Postpartum hemorrhage	○	●
Uterine atony	○	●
Lacerations	○	●
Ovular remains	○	●
Coagulation defect	○	●

METABOLIC DISORDERS (no / yes)

MELLITUS DIABETES (no / yes)

	no	yes	not performed
Abnormal oral tolerance to glucose load	○	●	○
Previous M. D. (insulin dependent)	○	●	
Previous M. D. (non insulin dependent)	○	●	
Gestational DM	○	●	
Hyperosmolar state	○	●	
Cetoacidosis	○	●	
Hyperglycemic state	○	●	

THYROID DISORDERS (no / yes)

	no	yes
Hypothyroidism	○	●
Hyperthyroidism	○	●
Thyroid crisis	○	●
Another metabolic disorder	○	●

OTHER DISORDERS (no / yes)

	no	yes
Hyperemesis gravidarum	○	●
Deep vein thrombosis	○	●
Pulmonary embolism	○	●
Amniotic fluid embolism	○	●
Heart disease	○	●
Valvulopathy	○	●
Seizures	○	●
Altered state of consciousness	○	●
Oliguria	○	●
Anemia	○	●
Sickle cell anemia	○	●
Renal disease	○	●
Malignant neoplasia	○	●
Psychiatric disorder	○	●
Cholestasis	○	●
Others	○	●

OBSTETRIC COMPLICATIONS (no / yes)

	no	yes
Obstructed labor	○	●
Prolonged rupture of membranes	○	●
Oligoamnios	○	●
Other	○	●
Polyhydramnios	○	●
Acute fetal distress	○	●
Intrauterine growth restriction	○	●

INTERVENTIONS

	no	yes	s/d
Manual removal	○	●	○
Uterotonic to treatment of hemorrhag	○	●	○
Central venous catheter	○	●	○
Administration of blood products	○	●	○
Laparotomy (excluding caesarean section)	○	●	○
ICU admission <7 days	○	●	○
Using ATB iv to treatment of infectious complication	○	●	○

Which ones?______ Nº of vol. days Which ones?______

	no	yes
No pneumatic shock-suit	○	●
Hydrostatic balls	○	●
B-Lynch suture or similar	○	●
Hemostatic ligature of uterine / hypogastric arteries	○	●
Embolizations	○	●

VARIABLES TO IDENTIFY NEAR MISS CASES (no / yes)

CLINICAL CRITERIA / BODY-SYSTEM DYSFUNCTION

	no	yes
Cardiovascular		
Shock	○	●
Heart attack	○	●
Hepatic		
Jaundice in preeclampsia	○	●
Respiratory		
Acute cyanosis	○	●
Wheezing (gasping)	○	●
Severe tachypnea RF> 40 rpm	○	●
Severe bradypnea RF <6 rpm	○	●
Renal		
Oliguria non responsive to fluids or diuretics	○	●
Hematological/coagulation		
Failure to form clots	○	●
Neurological		
Coma	○	●
Prolonged unconsciousness (lasting >12 hours	○	●
Stroke	○	●
Uncontrollable fits/status epileptics	○	●
Total paralysis	○	●

LAB

Laboratory	no	yes
Platelets/ml < 50.000	○	●
Creatinine ≥ 300 µmol/l or ≥ 3,5 mg/dl	○	●
Bilirubin > 100 µmol/l or > 6,0 mg/dl	○	●
pH < 7,1	○	●
Sat Hb < 90% ≥ 1 hour	○	●
PaO_2/FiO_2 < 200 mmHg	○	●
Lactato > 5 µmol/L o 45 mg/dl	○	●

INTERVENTIONS

	no	yes
Use of continuous vasoactive drug	○	●
Intubation and ventilation not related to anesthesia	○	●
Massive transfusion of blood or red cells (≥ 3 units)	○	●
Admission to UCI ≥ 7 days	○	●
Histerectomy	○	●
Dialysis for acute renal failure	○	●
Cardiopulmonary resuscitation	○	●

Which ones?______ days days

Criteria Near Miss: FLASOG ≥ 3 vol. WHO ≥ 5 vol.

PUERPERIUM

day	Temp ºC	blood prasure	pulse	uter. invol.	lochia	perine	breast feeding	observations	Responsabile
1st	,								
2nd	,								
3rd	,								
5th to 10th	,								

MATERNAL DISCHARGE

DATE: day month year hour min

TYPE OF DISCHARGE: dead ● against medical advise ● discharge ○

CONDITION AT DISCHARGE: healthy ○ NA ● with pathology ● dead ●

Place Transfer | passes away during or after transfer: no ○ yes ● | Autopsy: no ○ yes ●

Responsabile

CONTRACEPTION

Contraception councelling: oral ○ written ○ none ●

Responsabile

	no	yes
Start contraception	●	○

	pref.	acced.
IUD	○	○
injectable	○	○
barrier	○	○
tube ligation	○	○
hormonal contraception	○	○
Other hormonal (vaginal ring, patch)	○	○
male surgical sterilization	○	○
hormonal implant	○	○
condom	○	○
abstinence	○	○

This color means WATCH (does not necessarily indicate risk or inadequate practices)

HCP NEAR MISS REVERSO (Regional) INGLES 06-08-2018

For peer review only

NAME | Institution
LAST NAME | Country code | Type of doc. | ID number

Had COVID-19 in the 12 months before the pregnancy termination?

	yes	no	Date (month / day / year)	Diagnostic test: PCR	Rapid	doesn't know	Indicate the greater severity of the contagion(s) during pregnancy: Asymptomatic - home	Symptomatic - home	Inpatient – intermediate	Inpatient – intensive
Before pregnancy			month / day / year							
During pregnancy 1st. contagion			month / day / year							
During pregnancy 2nd contagion			month / day / year							

Did she receive any dose of the COVID-19 vaccine in the last year? yes no **(If yes, indicate from the most recent to the oldest)**

Date (month / day / year)		Gestational age	Did she present certificate/card? yes	no	AstraZeneca (AZD1222, ChAdOx1 nCoV-19, ChAdOx1-S, Vaxzevria, Covishield)	Janssen (JNJ-78436735; Ad26.COV2-S)	Pfizer/BioNtech (BNT162b2; Tozinameran; Comirnaty)	Sputnik V (Gamaleya; Gam-COVID-Vac; Adeno-based (rAd26-S+rAd5-S))	Sinopharm/ BIBP (Sinopharm/BIB)	Sinovac (CoronaVac; VeroCell)	Moderna (mRNA-1273; Elasomeran)	doesn't know	Other
month / day / year	or												Which one?:
month / day / year	or												Which one?:
month / day / year													Which one?:

For peer review only
For peer review only - http://bmjopen.bmj.com/site/about/guidelines.xhtml

Supplemental material III. Births attended and estimated number of potential cases per year.

Country	Hospital Name	Deliveries per year	Number of potential events per year		
			Small for gestational age	Preterm	Low birth weight
Bolivia	Hospital Boliviano Japonés	2719	177	337	185
	Maternidad de la Paz	1500	98	186	102
Colombia	Clínica Maternidad Rafael Calvo	7000	455	868	476
	Hospital Universitario del Valle, Cali	7200	468	893	497
Honduras	Hospital Roberto Suazo Cordova; Hospital de San Felipe; Leonardo Martinez Valenzuela; Hospital Berta Calderón Roque	27300	1775	3385	1856
República Dominicana	Hospital San Lorenzo de Los Mina	13000	845	1612	884
Argentina	Maternidad Martin	4200	273	349	291
	Roque Saenz Peña	2500	163	310	170
	Eva Peron	1500	98	186	102
Ecuador	Isidro Ayora	7000	455	868	476
Uruguay	Centro hosp Pereira Rosell	5000	325	620	340
	Hospital de Clinicas	800	52	99	54
Total SIP PLUS	-	93962	5731	10932	5995

For peer review only

Figure. Schematic example of the matching strategies for PTB cases: Approach A "matched by date of birth" and Approach B " matched by conception time"

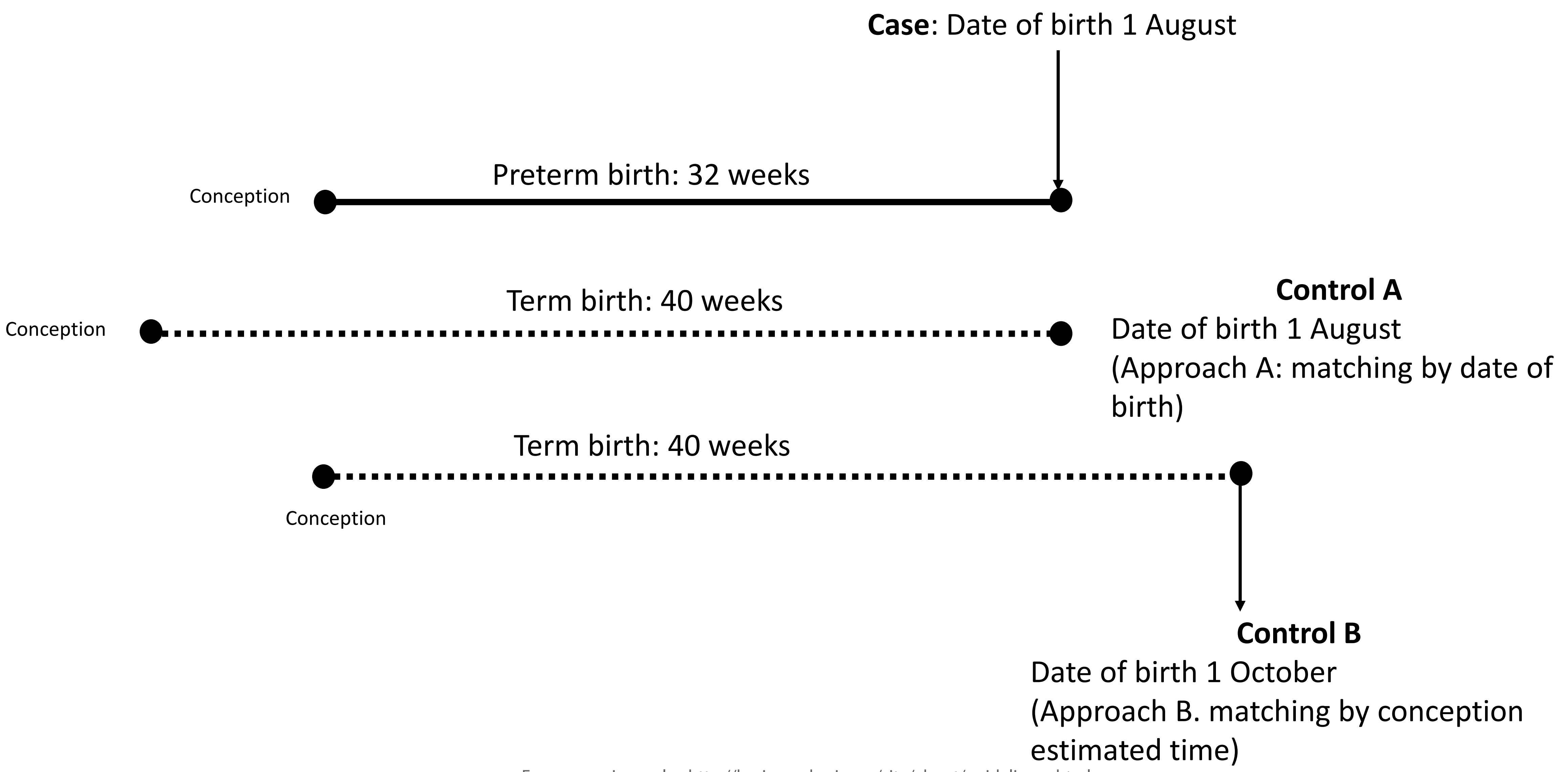

Supplement: Author's manuscript [file bmjopen-2023-073095.draft_revisions.pdf]
